# Proximity-Induced Nucleic Acid Degrader (PINAD) Approach to Targeted RNA Degradation Using Small Molecules

**DOI:** 10.1021/acscentsci.3c00015

**Published:** 2023-04-26

**Authors:** Sigitas Mikutis, Maria Rebelo, Eliza Yankova, Muxin Gu, Cong Tang, Ana R. Coelho, Mo Yang, Madoka E. Hazemi, Marta Pires de Miranda, Maria Eleftheriou, Max Robertson, George S. Vassiliou, David J. Adams, J. Pedro Simas, Francisco Corzana, John S. Schneekloth, Konstantinos Tzelepis, Gonçalo J. L. Bernardes

**Affiliations:** † Yusuf Hamied Department of Chemistry, University of Cambridge, Lensfield Road, Cambridge CB2 1EW, U.K.; ‡ Instituto de Medicina Molecular João Lobo Antunes, Faculdade de Medicina, Universidade de Lisboa, Avenida Professor Egas Moniz, 1649-028, Lisboa, Portugal; § Wellcome-MRC Cambridge Stem Cell Institute, University of Cambridge, Cambridge CB2 0AW, U.K.; ∥ Milner Therapeutics Institute, University of Cambridge, Puddicombe Way, Cambridge CB2 0AW, U.K.; ⊥ Chemical Biology Laboratory, Center for Cancer Research, National Cancer Institute, Frederick, Maryland 21702, United States; □ Experimental Cancer Genetics, Wellcome Trust Sanger Institute, Hinxton, Cambridge CB10 1SA, U.K.; ∇ Católica Biomedical Research and Católica Medical School, Universidade Católica Portuguesa, 1649-023 Lisboa, Portugal; ○ Departamento de Química, Centro de Investigación en Síntesis Química, Universidad de La Rioja, 26006 Logroño, Spain

## Abstract

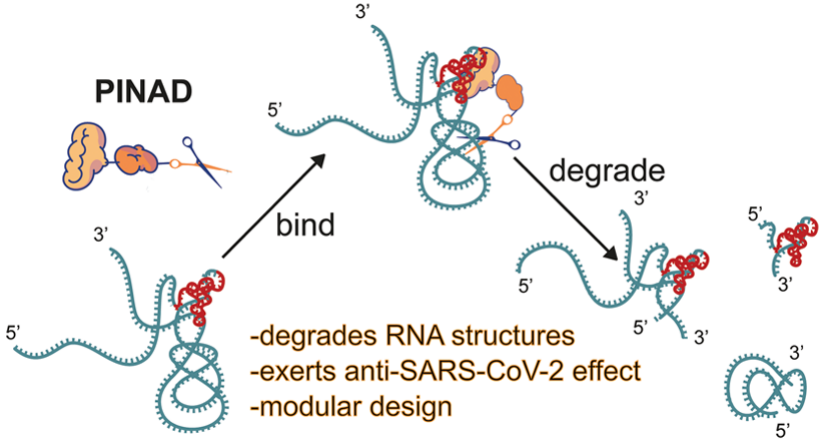

Nature has evolved intricate machinery to target and degrade RNA, and some of these molecular mechanisms can be adapted for therapeutic use. Small interfering RNAs and RNase H-inducing oligonucleotides have yielded therapeutic agents against diseases that cannot be tackled using protein-centered approaches. Because these therapeutic agents are nucleic acid-based, they have several inherent drawbacks which include poor cellular uptake and stability. Here we report a new approach to target and degrade RNA using small molecules, proximity-induced nucleic acid degrader (PINAD). We have utilized this strategy to design two families of RNA degraders which target two different RNA structures within the genome of SARS-CoV-2: G-quadruplexes and the betacoronaviral pseudoknot. We demonstrate that these novel molecules degrade their targets using *in vitro*, *in cellulo*, and *in vivo* SARS-CoV-2 infection models. Our strategy allows any RNA binding small molecule to be converted into a degrader, empowering RNA binders that are not potent enough to exert a phenotypic effect on their own. PINAD raises the possibility of targeting and destroying any disease-related RNA species, which can greatly expand the space of druggable targets and diseases.

## Abstract 2

An approach to convert RNA-binding molecules into RNA degraders is described. Reported molecules are found to affect the SARS-CoV-2 genome *in vitro*, in cells, and *in vivo*.

## Introduction

RNA is a structured biomolecule, and certain RNA structures can be attributed to pathologies. Moreover, the majority of the human genome is transcribed into RNA, whereas only ∼3% of RNA transcripts get translated into proteins, meaning that for each pathology, there are potentially many more disease-relevant RNA species than proteins.^1^ Not surprisingly, many natural products or natural product-derived therapeutics exert their mechanism of action by binding RNA, and it is not unlikely that small molecules thought to exert their activity through binding proteins also bind RNA.^2^ One of the most well explored classes of these molecules are rRNA binding antibiotics. For example, tetracyclines bind the 16S rRNA on the 30S subunit of prokaryotic ribosomes, thus blocking the tRNA docking onto the A-site and interfering with the translation of prokaryotic proteins, which is the underlying mechanism behind the bacteriostatic effect.^3^ Strikingly, mutation of a few key bases on 16S rRNA results in the loss of binding contacts against tetracyclines leading to resistance against this class of molecules. While tetracyclines exert their mechanism of action by sterically blocking access to the ribosome, for most RNA effectors a binding event might not be sufficient to modulate the functions of the targeted RNA. This issue may be circumvented by converting RNA binders into bifunctional molecules which can alter the functions of their target RNAs, e.g., by degrading or editing them.

**Figure 1:**
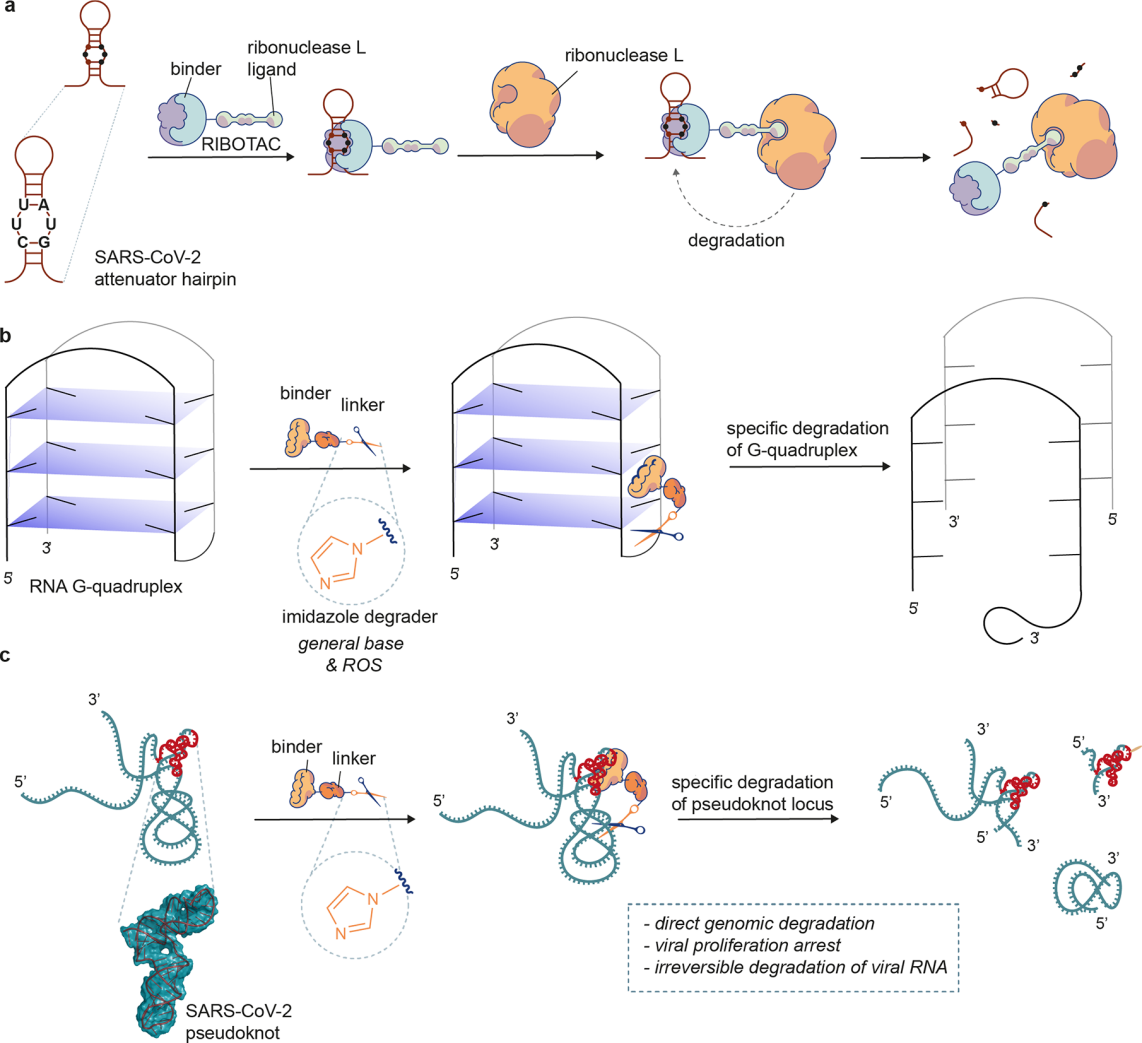
Schematic diagram of the mode of action of RNA degraders. (a) RIBOTAC approach to targeted RNA degradation. To achieve degradation, the RIBOTAC needs to form a ternary complex with the target RNA and ribonuclease L. (b) Outline of our RNA G-quadruplex degradation strategy. The G4-degrader binds and degrades the G-quadruplex. (c) Outline of our coronaviral pseudoknot degradation strategy. The pseudoknot-degrader binds and then directly degrades the coronaviral region that contains the pseudoknot without a need for other cellular factors. ROS, reactive oxygen species.

Degradation of RNA utilizing small molecule RNA binders has been previously attempted in the form of RIBOTACs (ribonuclease targeting chimeras, Figure 1a).^4^ In the RIBOTAC approach, a small molecule RNA binder is appended to a ligand of ribonuclease (all the examples in the literature to this point utilize ribonuclease L). The two distinct parts of this bifunctional molecule bring together the target RNA and a ribonuclease, which results in target RNA getting degraded. Thus, RIBOTACs act akin to siRNAs but have several caveats which in some cases can limit their therapeutic potential.^5^ First, activities of RIBOTACs described thus far depend on endogenous concentrations of ribonuclease L which is not evenly expressed across different tissues; thus, not all cell types would be compatible with this approach. Second, for RIBOTACs to function, the RNA and RNase L ligands need to have spatial orientation compatible with bringing the two biomacromolecules together, the RNA and an enzyme. Moreover, RIBOTACs conjoin two small molecule ligands having large molecular weights, which might negatively affect their physiochemical properties. It is also worth noting that RIBOTACs disclosed so far have a limited effect on their target RNA, with degradation usually capping at 50–60%. However, this might be a consequence of the fast turnover of RNA species targeted rather than an inherent feature of RIBOTACs. Altogether, this calls for alternative approaches to small molecule-induced targeted RNA degradation.

Inspired by the mechanisms of ribonucleases^6^ and with a desire to circumvent some of the aforementioned issues of RIBOTACs, we envisaged a small molecule which can degrade nucleic acids in a targeted manner by being in a close proximity of its target, which we have named proximity-induced nucleic acid degrader (PINAD). In our design, PINADs are composed of three components—a small molecule RNA binder, a long flexible linker which can reach multiple positions on the targeted RNA, and an imidazole—the RNA degrading moiety present on many ribonucleases. We have previously shown that it is sufficient to covalently attach imidazole moieties onto RNA to result in their degradation; we thus postulated that bringing an imidazole into proximity will have comparable results.^7^ The Duca group has recently reported a similar approach, in which they weaponized the aminoglycoside antibiotic neomycin with an amino acid histidine.^8^ For our studies, we developed two series of PINADs using imidazole warheads conjugated to flexible PEG chains, targeting two different structural elements of the genome of SARS-CoV-2, and demonstrated that these molecules are active using *in vitro*, *in cellulo*, and *in vivo* infection models of SARS-CoV-2 (Figure 1b,c).

## Results and Discussion

### Targetable Structural Elements in the SARS-CoV-2 Genome

As a proof of concept, we elected to test the PINAD approach against the genome of SARS-CoV-2 because it possesses genomic RNA with multiple characterized structural features and is disease-relevant, being the carrier of genetic information of the virus that causes COVID-19.^9^ Indeed, Disney and colleagues have also applied the RIBOTAC approach against the SARS-CoV-2 genome, developing a molecule that targets an attenuator hairpin and recruits ribonuclease L.^10^ Thus, we designed PINADs that degrade G-quadruplexes^11^ and betacoronaviral pseudoknots,^12^ two structural motifs found on the genome of SARS-CoV-2 (Figure 1b,c). The G-quadruplex is an RNA motif comprised of several stacks of G-quads (G4)—guanine tetramers held together through hydrogen bonding between Hoogsten and Watson–Crick-Franklin interfaces. The SARS-CoV-2 genome is populated by four putative G4 sequences—one each in *N* and *nsp10* genes (formation confirmed experimentally) and two in the *S* gene (not confirmed experimentally).^11^ It was demonstrated that the presence of the G4 structure in the *N* gene reduces the efficiency of the nucleocapsid phosphoprotein (N protein) translation, suggesting that this RNA structure is utilized to maintain the optimal ratio of viral proteins.^11^ The betacoronaviral pseudoknot is a more complex and unique structure, consisting of three stem-loops.^12^ This structure has a well-defined role in −1 frameshifting—a process in which the ribosome shifts by one nucleotide to the 5′ direction resulting in a shifted reading frame; this process is used by SARS-CoV-2 for translation of RNA-dependent RNA polymerase *Nsp12*.^13^ Since this enzyme is vital for replication of this virus, disruption of −1 frameshifting can severely hamper the rate of replication for SARS-CoV-2.^14^ This is a likely explanation for why the sequence corresponding to the pseudoknot is so well conserved—it is uniform throughout different SARS-CoV-2 variants of concern, and the homology between the pseudoknots in SARS-CoV and SARS-CoV-2 is 99% (difference of a single nucleotide), much higher than that of the overall genome. The rapid mutation rates of viral genomes make targeting of the conserved regions highly desirable.^15^

### Design of PINADs

**Figure 2:**
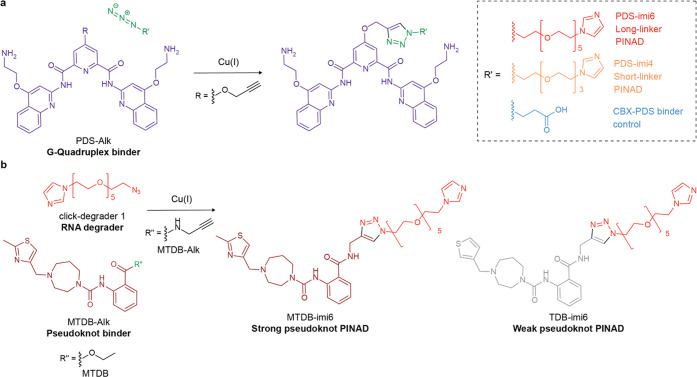
Chemical design of PINADs. (a) Synthetic design of **PDS** family of PINADs. (b) Synthetic design of **MTDB** family of PINADs. See Supporting Information for complete synthesis procedures; characterization **PDS** has been previously functionalized on its central pyridine core, and it was shown that this functionalization does not abolish its binding ability.^19^ As such, we chose to modify it on this position. **MTDB**, on the other hand, has not been further modified previously. However, as it was discovered through molecular docking, the docking pose provided insight into where the molecule could be functionalized without compromising its binding affinity. We thus chose to transform a terminal ethyl ester into a propargylic amide (more stable in cellular environments than an ester), which was in turn functionalized with the azide-degraders.

Both the aforementioned RNA structures have previously described binders, which we used to design two series of PINADs and appropriate control compounds. For G4s, we chose pyridostatin (**PDS**), a well-explored ligand with a good selectivity and *K*_D_ in the nanomolar range.^16^ For the pseudoknot, we chose **MTDB**, a pseudoknot ligand identified through an *in silico* screen and demonstrated to affect the −1 frameshifting,^17^ reported to bind the pseudoknot in the upper micromolar range.^18^ For both series of compounds, we functionalized the binders with alkynes so they could be readily exposed to a copper-catalyzed alkyne–azide cycloaddition (CuAAC) reaction. We made **PDS** derivatizes with degraders of two different lengths so we could investigate how the length of the linker affects the efficiency of degradation. We also functionalized **PDS** with a carboxy group as a control (**CBX-PDS**), shown previously to selectively bind RNA (Figure 2a).^19^ For the design of the **MTDB**-based degrader, we utilized a longer linker that we found to be more efficient for the **PDS** system as well as made a degrader from a weaker pseudoknot binder TDB, expecting to obtain a weaker analogue (Figure 2b).^17^

### *In Silico* Study of Pseudoknot-Targeting PINADs

To gain insight into how an attachment of a linker and a degrader could affect the ability of **MTDB** and **TDB** to bind the betacoronaviral pseudoknot, we carried out molecular docking studies. For this aim, putative 3D structures of the complexes between **MTDB-imi6** or **TDB-imi6** and the pseudoknot were generated using the cryo-EM structure of the pseudoknot (PDB id: 6XRZ). AUTODOCK 4.2^20^ was used for the calculations (see Methods for details). As inferred from these calculations, both ligands can interact with several regions of the pseudoknot (Figure S1). One of the best poses for **MTDB-imi6** (pose #3, Figure S1a,c) featured a hydrogen bond between the thiazole ring and U45 (the numbering of the nucleotides used for the cryo-EM structure^21^ was applied in this manuscript), which was absent in **TDB-imi6**. Additionally, the amide group of **MTDB-imi6**, the tertiary amine, and one of the oxygen atoms of the PEG linker were engaged in hydrogen bonding interactions with A76, C17, and C71, respectively (Figure S1c). The stabilizing interaction between the thiazole ring and the receptor has been identified as a key intermolecular interaction^21^ that could contribute to the efficiency of the degradation process. The binding energy calculated by AUTODOCK 4.2 for the best ranked poses of **MTDB-imi6** and **TDB-imi6** was −8.02 and −5.23 kcal/mol, respectively (Figure S1b,d). Interestingly, the best binding pose suggested that our calculations, which are based on the cryo-EM structure of the pseudoknot,^21^ differ from what was proposed by Park and co-workers,^17^ who used a model of the pseudoknot to predict the 3D structure of the complexes. However, overall findings of this study suggested that exchanging an ester into an amide will not abolish binding and that the **MTDB**-derived degrader has a higher affinity to the pseudoknot compared to the **TDB**-derived degrader.

The complex between **MTDB-imi6** (pose #3) and the pseudoknot (see Methods for details) was then subjected to extensive molecular dynamic (MD) simulations in the presence of explicit water molecules and ions (Figure S1e). According to these simulations, the complex is stable through the trajectory but explores several regions of the receptor. In addition, the PEG linker was quite flexible, allowing interactions between the imidazole degrader and different sites of the pseudoknot. The flexibility of the complex resulted in several structures that exhibited different transient hydrogen bonds, especially between the triazole moiety or the oxygen atoms of the linker and the RNA molecule, which could explain the higher affinity of **MTDB-imi6** compared to **MTDB**. The most important hydrogen bond (population ∼20%) engaged the carbonyl of the amide group and G46. The complex was also stabilized by transient π-staking interactions between the phenyl group of the ligand and several bases of the pseudoknot. These findings suggest that the long linker present on the **MTDB-imi6** enables the degrader moiety to reach many nucleotides on this structure.

### Binding Affinity of PINADs

**Figure 3:**
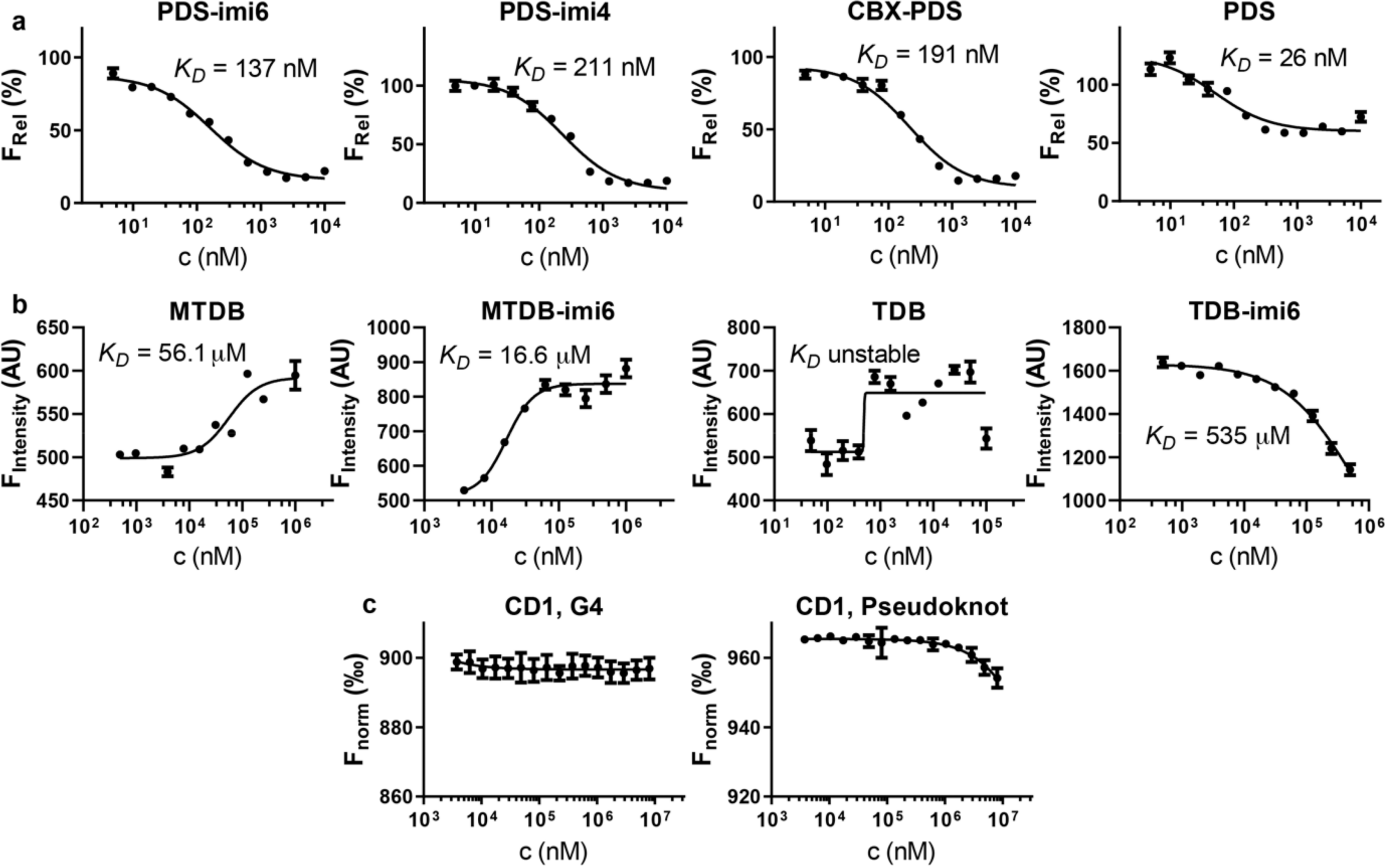
Binding affinities of PINADs and their parent molecules. (a) Affinity measurements of the **PDS** family toward the 5′ UTR NRAS oligonucleotide in a K^+^ buffer, as determined via a fluorescence quenching assay. (b) Affinity measurements of the **MTDB** family toward a betacoronaviral pseudoknot oligonucleotide, as measured via an MST initial fluorescence scan. (c) Affinity measurements of Click-Degrader 1 toward the G4 and pseudoknot constructs, as measured via MST thermophoresis curve analysis (*n* = 3).

Having prepared the two series of compounds, we experimentally tested how functionalization with degraders has affected their binding affinities and selectivity. **PDS** is a known fluorescence-quencher, and thus we utilized a fluorescence quenching assay to evaluate compounds derived from this scaffold.^22^ In brief, **PDS** and its derivatives were incubated with a Cy5-functionalized oligomer corresponding to a G-quadruplex in NRAS 5′UTR, a well-established G4 model. As fluorescence quenching is proximity-induced, the signal corresponding to Cy5 is quenched upon **PDS** binding. We found all the compounds functionalized on the central pyridine core to have a similar binding affinity, close to 200 nM, with the parent compound **PDS** having a stronger affinity with *K*_D_ of 26 nM (Figure 3a). This is expected as the parent compound has an additional amine group on the pyridine core which has been replaced with a triazole in the derived compounds. This positively charged amine group in the parent compound might be forming additional interactions with the negatively charged phosphate backbone. Overall, these results show that for the **PDS** family of G4 binders functionalization with a degrader leads to a slightly reduced binding affinity but otherwise similar binding profile to the parent compound.

To determine the binding affinities of **MTDB** family ligands, we utilized the capillary format of a microscale thermophoresis (MST) reader (initial fluorescence scan). In an MST workflow, a 5′-Cy5-labeled oligomer corresponding to the coronaviral pseudoknot is incubated with one of the binders. With the observation that the fluorescence was affected by bound ligand, the binding curve could be obtained in a concentration-dependent manner by an initial capillary scan. We found that **MTDB** exhibited a *K*_D_ value of 56.1 μM (Figure 3b), while in a prior study where surface plasmon resonance (SPR) was used, **MTDB** was determined to have a *K*_D_ of 210 μM.^18^ This difference might be a result of the format of the assays used, as SPR measures binding on a surface, whereas MST does so in a solution.

The two degrader-functionalized MTDB derivatives, **MTDB-imi6** and **TDB-imi6**, were found to have *K*_D_ values of 16.6 μM and 535 μM, respectively (Figure 3b). This result indicates that **MTDB-imi6** indeed binds the pseudoknot more tightly relative to **TDB-imi6**. This also suggests that in the case of **MTDB**, functionalization with a degrader enhances the binding affinity, which is consistent with our *in silico* observations (Figure S1c). However, it cannot be discarded that some degradation could be taking placing during the measurement, which would influence the observed *K*_D_ value. We also attempted to measure the binding affinity of **TDB** by using the same approach, but we were not able to establish a *K*_D_ due to limited solubility of the molecule.

As we observed enhanced binding affinity of **MTDB-imi6** relative to **MTDB**, we investigated whether the degrader alone could bind to the G4 or the pseudoknot. Because the degrader itself does not influence the fluorescence of the RNA constructs, we used the thermophoresis curves of the MST approach to estimate binding affinities. For both systems, binding was observed only at millimolar concentrations, and thus we could not establish *K*_D_ values (Figure 3c). These results show that for the PINADs we have made, the main determinant of binding is the binding moiety, with the degrader playing a very minor role.

### PINADs Can Degrade Their Targets *in Vitro*

Confident in the ability of our molecules to bind their target structures, we tested their capacity for degradation *in vitro*. First, we evaluated whether PINADs can degrade oligonucleotides which form a G4 or the betacoronaviral pseudoknot structure. For this aim, we utilized liquid chromatography–mass spectrometry (LC-MS), with the results observed with the pseudoknot further validated via gel electrophoresis. Briefly, for the LC-MS assays we ensured that the relationship between injected oligonucleotide concentration and mass signal was linear (Figure S2a,b), and then we used the method to quantify oligonucleotides after incubation with an appropriate PINAD (Figure S2c, detailed method in the Supporting Information). We also considered using UV–vis spectra for quantification, and although it showed good evidence for degradation, we elected not to use it due to high noise levels and the necessity in applying a noise subtraction algorithm, which biases numerical data (Figure S2d).

**Figure 4:**
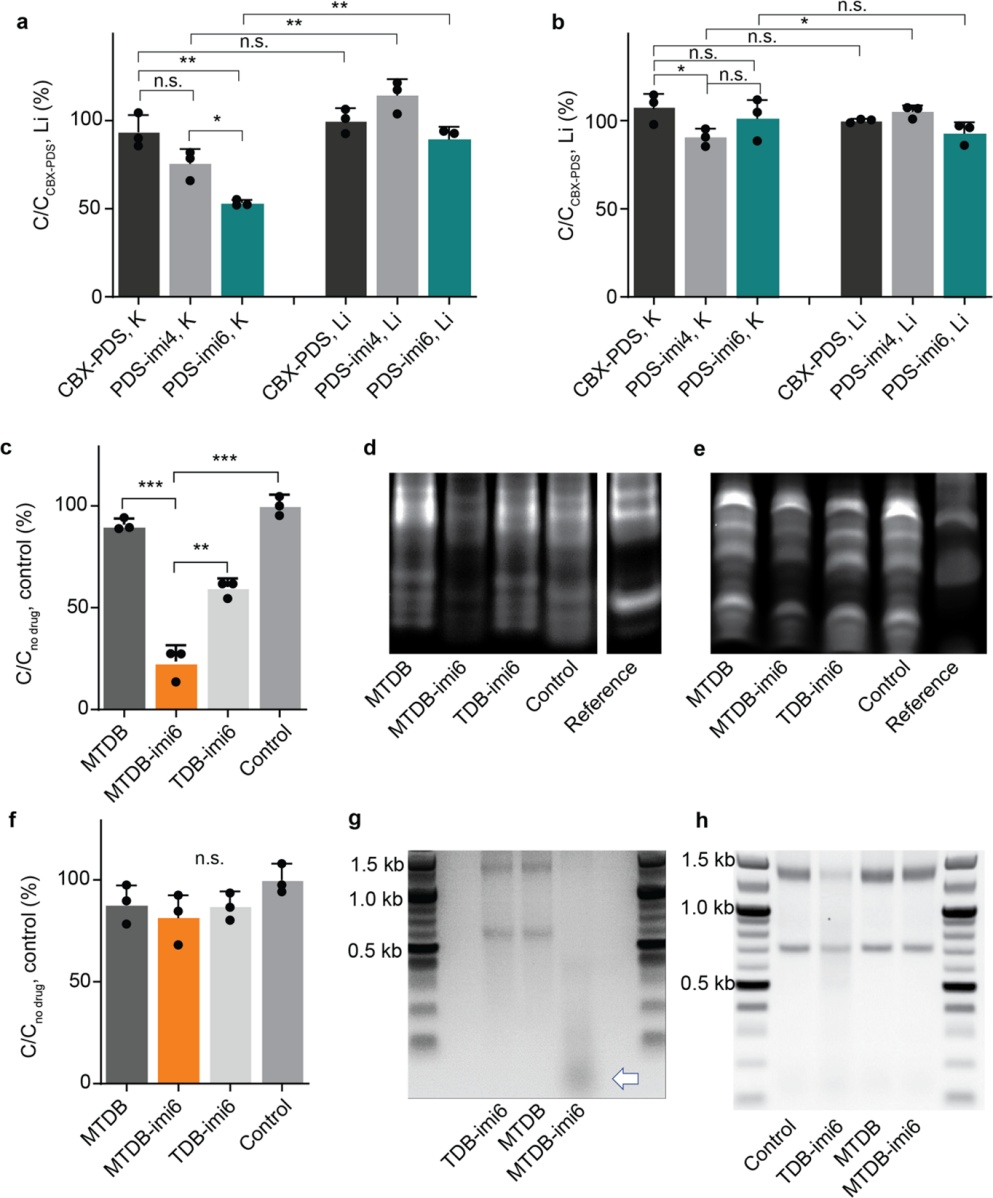
PINADs degrade their target RNAs *in vitro*. (a) LC-MS validation of the G4 degraders against a G4-forming oligonucleotide after a 4 h 37 °C incubation with a specified molecule (*n* = 3). (b) LC-MS validation of the absence of cutting against a non-G4-forming oligonucleotide after a 4 h 37 °C incubation with a specified molecule (*n* = 3). (c) LC-MS validation of the pseudoknot degraders after a 3 h 37 °C incubation with a specified molecule (*n* = 3). (d) Nondenaturing gel validation of pseudoknot degraders on a 5′FAM-tagged pseudoknot oligonucleotide after a 6 h 37 °C incubation with a specified molecule. Double band for the pseudoknot might correspond to a pseudoknot monomer and dimer.^24^ (e) Denaturing gel validation of pseudoknot degraders on a 5′FAM-tagged pseudoknot oligonucleotide after a 6 h 37 °C incubation with a specified molecule. Representative gel shown (*n* = 3). (f) LC-MS validation of pseudoknot degraders losing efficiency when one of the pseudoknot stems is mutated and perturbs the pseudoknot secondary structure (*n* = 3). (g) Agarose gel electrophoresis validation of the cutting (white arrow) of extracted native SARS-CoV-2 RNA. (h) Agarose gel electrophoresis validation of no cutting of RNA extracted from HEK293FT cells. n.s. = not significant, *p < 0.05, **p < 0.01, ***p < 0.001.

We tested degradation capability of the degraders and the control molecule derived from **PDS** by incubating them with the G4-forming NRAS oligonucleotide at 37 °C in either potassium-containing (promotes G4 formation by stabilizing them) or lithium-containing (does not stabilize G4 structures) buffer, followed by LC-MS analysis. In the potassium buffer, we observed a significant amount of degradation with both **PDS-imi6** and **PDS-imi4** relative to **CBX-PDS**, with the **PDS-imi6** being a more efficient degrader, which shows that an appended imidazole can indeed act as a degrader, and for this molecule, the longer linker performs the best, whereas in the lithium-containing buffer little to no degradation was observed, as would be expected in a system where the population of formed G4 structures is not promoted by present cations (Figure 4a).^23^ Degradation was no longer observed when the same workflow was carried out on a perturbed NRAS oligomer which cannot form a G4 structure, which shows that under these conditions the PDS-based PINADs affect only the G4 structures (Figure 4b).

Similar outcomes were observed with the **MTDB-PINADs**. Molecules in these series were incubated with the pseudoknot-oligomer for 3 or 6 h, resulting in extensive degradation with **MTDB-imi6** and less extensive degradation with **TDB-imi6**, as shown both using LC-MS and gel electrophoresis (Figure 4c–e). To gain further insight into degradation, we incubated FAM-tagged pseudoknot oligonucleotides containing 3′ overhangs with varying concentrations of PINADs and control molecules. Gel analysis has revealed a dose-dependent degradation with both **MTDB-imi6** and **TDB-imi6** but not the control molecules **MTDB** and **Click-Degrader 1** (Figure S3a–d). The 3′ overhangs allowed for a better visualization of the pseudoknot degradation compared to the non-extended oligonucleotide. No significant degradation was observed when these molecules were incubated with the perturbed pseudoknot, demonstrating their selectivity toward the folded betacoronaviral pseudoknot (Figure 4f). Additionally, and to show that MTDB-derived PINADs can also cut full-length RNA, we incubated **MTDB**, **TDB-imi6**, and **MTDB-imi6** with RNA extracted from SARS-CoV-2. In this experiment, RNA from SARS-CoV-2 was obtained by harvesting the supernatant from in vitro cultures of cells infected with SARS-CoV-2, which was ultracentrifuged to concentrate the viral particles before RNA extraction. Importantly, we observed degradation only in the lane corresponding to **MTDB-imi6** further confirming that **MTDB-imi6** does degrade the native coronaviral pseudoknot, whereas **TDB-imi6** might be too weak a pseudoknot binder under these conditions to affect the coronaviral RNA (Figure 4g). Interestingly, no degradation was observed with **MTDB-imi6** using total RNA extracted from the cell line HEK293FT (Figure 4h).

### Direct RNA Nanopore Sequencing and Genome-Fragment qPCR Confirm Target Engagement *in Vitro* and in a Cellular Model

**Figure 5:**
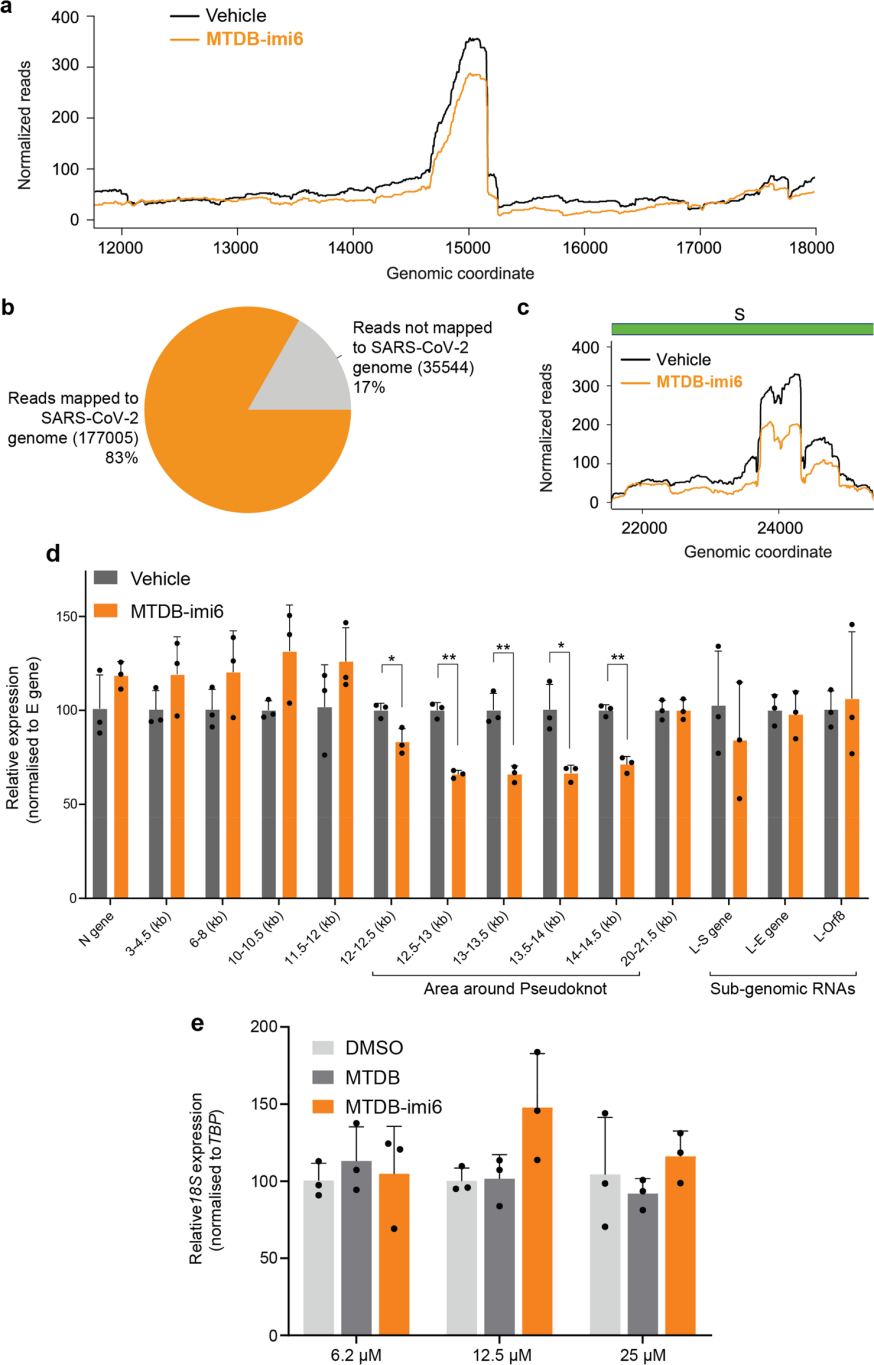
Evidence of target engagement for **MTDB-imi6**. (a) Distribution and abundance of aligned reads flanking the pseudoknot area for vehicle- or **MTDB-imi6**-treated SARS-CoV-2 RNA, based on alignments with minimap2. (b) Pie chart showing Nanopore reads mapped or unmapped to the SARS-CoV-2 genome. (c) Distribution and abundance of aligned reads mapped exclusively on the S subgenomic RNA region for vehicle- or **MTDB-imi6**-treated native SARS-CoV-2 RNA, based on alignments with minimap2. (d) RT-qPCR validation of degradation specificity in a cellular system using SARS-CoV-2 infected VERO-CCL-81 cells treated with either 6 μM **MTDB-imi6** or vehicle (DMSO) for 24 h (*n* = 3). (e) qPCR validation shows that **MTDB** and **MTDB-imi6** do not degrade or otherwise affect the abundance of 18S rRNA in the concentration range tested. Student’s *t* test. Mean + SD of three independent replicates is shown, *p < 0.05, **p < 0.01.

To clarify where **MTDB-imi6** cuts RNA, we analyzed the treated SARS-CoV-2 RNA via direct RNA Nanopore sequencing. As expected, the region around the pseudoknot was affected the most (Figure 5a). We observed that the pseudoknot flanks were more degraded than the pseudoknot itself, which is likely a result of a long linker and extensive short-range interactions within and around the frameshifting element. Also, QC analysis of our Nanopore data set showed that 83% of the reads mapped on SARS-CoV-2 genome in line with previously published studies;^25,26^ this shows that the pool of RNA we were analyzing contained predominantly SARS-CoV-2 RNA (Figure 5b). Interestingly, the only other structural element that was affected by the molecule was the S gene, which was shown to form long-range interactions with the ORF1b16 and therefore is expected to be within reach of the degrader (Figure 5c).^9^ Strikingly, none of the other subgenomic regions were affected, which provides strong evidence for the specificity of **MTDB-imi6** (Figure S4). To assess how well these observations translate to a cellular model, we treated VERO–CCL-81 cells infected with SARS-CoV-2 either with **MTDB-imi6** or a vehicle control, followed by RNA extraction and qPCR analysis of 15 loci of the SARS-CoV-2 genome to get a full genomic coverage and reveal which parts are affected the most. This validation was in broad agreement with Nanopore sequencing, demonstrating that **MTDB-imi6** affects the flanks of the pseudoknot area but not any other region of the tested subgenomic SARS-CoV-2 RNAs (Figure 5d). Furthermore, RT-qPCR validation confirmed that **MTDB-imi6** treatment of VERO-CCL-81 cells infected with SARS-CoV-2 has no effect on 18S rRNA levels (Figure 5e), in line with the results shown in Figure 4h. The above findings provide strong proof-of-principle that **MTDB-imi6** is a functional and selective degrader of the SARS-CoV-2 pseudoknot and its direct RNA–RNA interactome, which acts as a proof of concept for the PINAD mechanism.

### Antiviral Activity of PINADs

**Figure 6:**
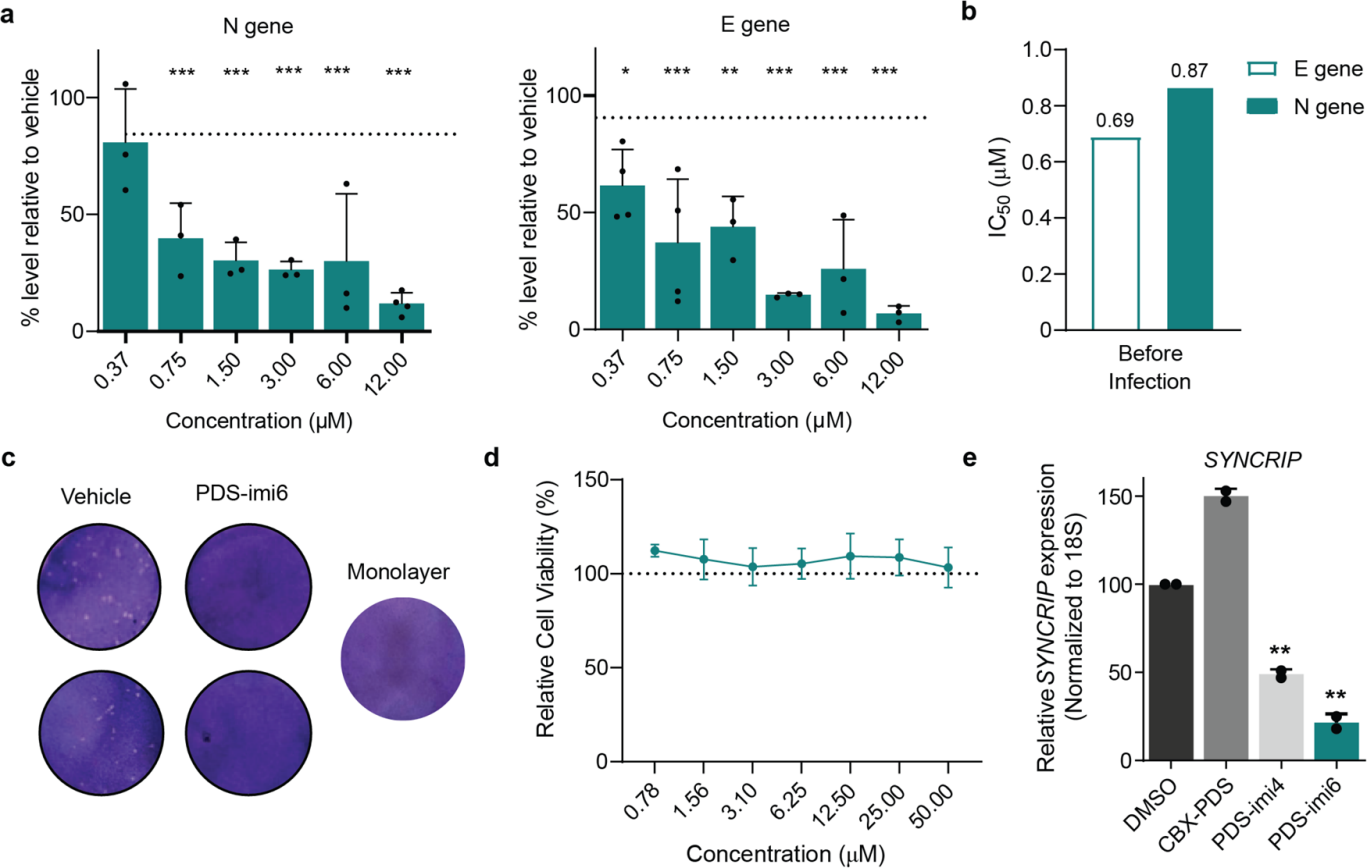
Antiviral effects of **PDS-imi6**. (a) The percentage inhibition of viral replication normalized to vehicle treated cells (dashed line) after incubation with increasing concentrations of **PDS-imi6**. Viral replication was assessed 24 h after infection [multiplicity of infection (MOI) of 0.05] based on E and N genes (*n* = 3). Mean ± SD of triplicates is shown, and differences between means with *p* < 0.01 are indicated. (b) Calculated IC_50_ values of **PDS-imi6** were determined by quantifying E or N gene. (c) 24-h treatment with **PDS-imi6** at 6 μM showed a decreased number of viral plaques in comparison to vehicle control. Images are representative of 4 independent experiments. (d) **PDS-imi6** did not show cytotoxicity in VERO-CCL-81 cells (*n* = 3). (e) RT-qPCR validation of degradation specificity in a cellular system using MOLM-13 cells treated with 5 μM of **CBX-PDS**, **PDS-imi4**, and **PDS-imi6** or vehicle for 24 h (*n* = 3). *p < 0.05; **p < 0.01; ***p < 0.001; two-tailed paired *t* tests versus control.

We tested the antiviral activity of the two families of PINADs by using SARS-CoV-2 infected VERO-CCL-81 cells as a model of SARS-CoV-2 infection. First, we tested the inhibition of viral replication in a dose-dependent manner. Viral loads were determined using qPCR on either the E (Envelope) or N (Nucleocapsid) gene. We found that **PDS-imi6** reduces viral replication in a dose-dependent manner with an IC_50_ of around 1 μM when cells were pretreated 1 h before infection (Figure 6a,b). Similar results were obtained by plaque assay, where treatment of cells with 6 μM of **PDS-imi6** led to a pronounced decrease in plaque-forming units (PFU) of the viral culture (Figure 6c, Figure S5a). We found that **PDS-imi6** had no effect on the viability of VERO-CCL-81 cells, and thus the observed effect cannot be explained by cell death (Figure 6d). However, **PDS-imi6** is likely to affect many G4 structures in the cell, and thus it cannot be discarded that the observed decrease of viral replication is a composite effect of degradation of the SARS-CoV-2 genome and modulation of infection-relevant endogenous transcripts. To test this hypothesis, we analyzed an mRNA *SYNCRIP*, reported in multiple studies to contain a G-Quadruplex.^27−29^ We found that treatment with the binder CBX-PDS led to increased levels of this transcript, whereas treatment with either of the two degraders led to a significant depletion (Figure 6e). This provides evidence that PINADs can target and degrade endogenous human RNA species. Additionally, we found that **PDS-imi6** has similar antiviral effects on the Alpha and Delta SARS-CoV-2 variants of concern (lineages B.1.1.7 and B.1.617.2, respectively), suggesting retention of G4 structures between these variants (Figure S5b,c).

**Figure 7:**
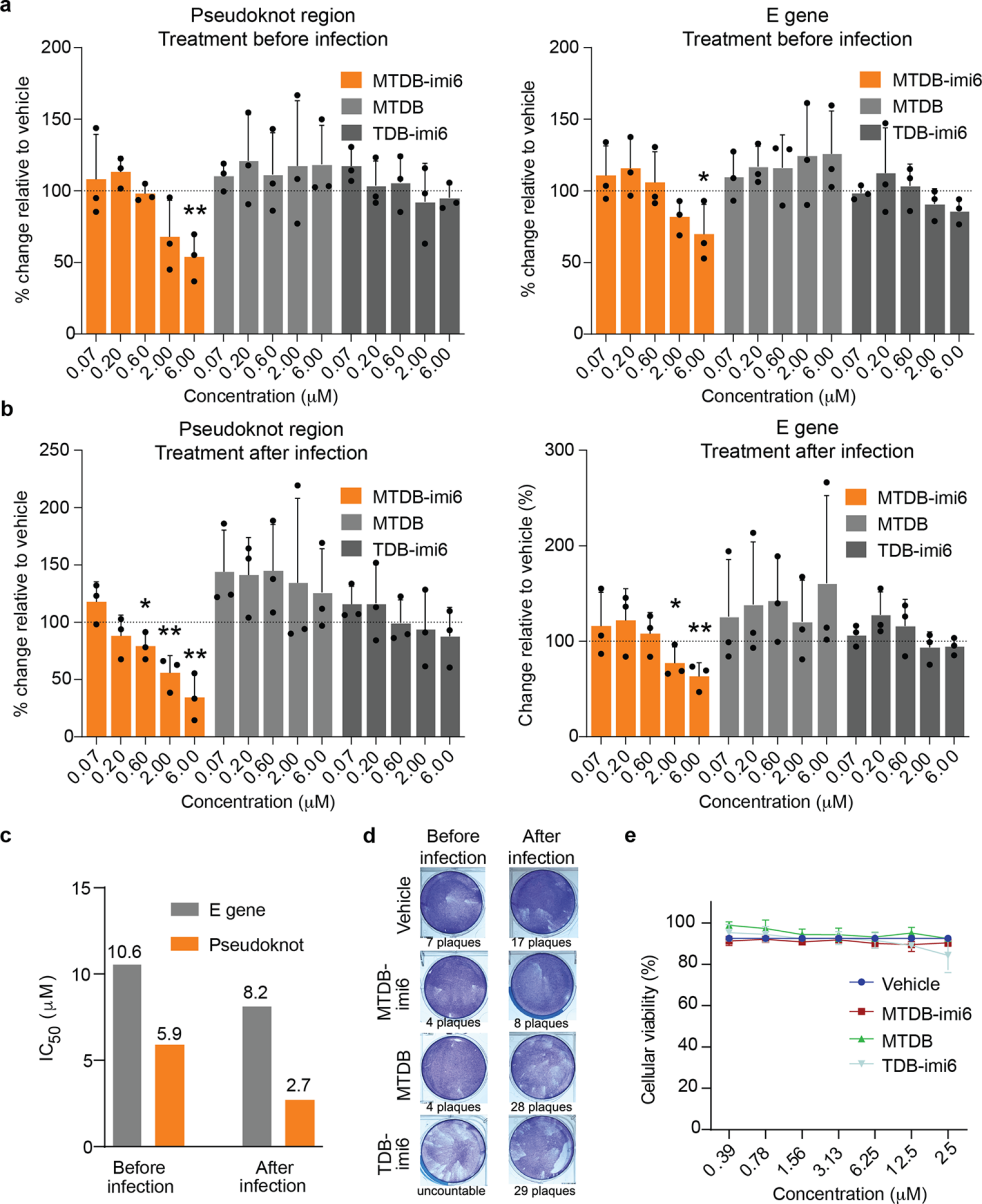
**MTDB-imi6** inhibits SARS-CoV-2 replication in cells. (a, b) The percentage inhibition of viral replication normalized to vehicle treated cells (dashed line) after incubation with increasing concentrations of the pseudoknot degrader (**MTDB-imi6**) and control molecules (**MTDB** and **TDB-imi6**). Viral replication was assessed 24 h after infection [multiplicity of infection (MOI) of 0.05] based on the E gene and pseudoknot region RNA levels (*n* = 3). Antiviral activity of the **MTDB-imi6** was observed when VERO-CCL-81 cells were treated before (a), or after infection (b), with SARS-CoV-2 at a 0.05 MOI. Mean ± SD of triplicates is shown, and differences between means with *p* < 0.01 are indicated. *p < 0.05; **p < 0.01; two-tailed paired *t* tests. (c) Calculated IC_50_ values of **MTDB-imi6** when determined by quantifying E gene or the pseudoknot locus. (d) 24-h treatment with **MTDB-imi6** at 6 μM showed a decreased number of viral plaques in comparison to vehicle treatment, both when added before or after infection. Control molecule **MTDB** showed only a decreased number of viral plaques when added before infection, and **TDB-imi6** treatment showed no decrease in plaque formation. (e) None of the compounds showed cytotoxicity in VERO-CCL-81 cells (*n* = 3). Mean ± SD of triplicates is shown.

**MTDB-imi6**, on the other hand, is only known to target the coronaviral pseudoknot and its RNA–RNA interactome and thus is a better PINAD for demonstration of specificity. As for the **PDS-imi6**, we tested the effect of the **MTDB** family of molecules on viral replication and evaluated it using qPCR, in this case probing the E gene and the pseudoknot region itself. We observed that low-micromolar concentrations of **MTDB-imi6** exhibited marked antiviral effects with a significant reduction of coronaviral RNA in cells treated before or after infection, having lower IC_50_ values when treating the cells postinfection (Figure 7a–c). This observation agrees with our proposed mechanism of action; as **MTDB-imi6** selectively targets the coronaviral pseudoknot it will only exert its effect postinfection. On the other hand, treatment preinfection would likely lead to **MTDB-imi6** being partially metabolized resulting in a lower effective concentration at the time of infection and thus a weaker effect. The other molecules in the series, **TDB-imi6** and **MTDB**, did not exhibit a significant antiviral effect, which demonstrates that PINADs function better when designed from a stronger binder, and degradation is indeed necessary for these molecules to exert their effect.

These findings were further supported by the results of the plaque assay—strongest effects on viral replication were observed with **MTDB-imi6** both when cells were treated before and after infection (Figures 7d and S5d). Moreover, to further demonstrate the anti-SARS-CoV-2 impact of **MTDB-imi6** treatment, we examined the status of two robust SARS-CoV-2 infection biomarkers. Indeed, treatment with **MTDB-imi6** significantly reduced the phosphorylation of MAPK2 (p-MK2 and T334) and led to a significant down-regulation of the mRNA of the key cytokine *IL6*, with both biomarkers known to be dictated by the p38/MAPK signaling pathway and elevated during SARS-CoV-2 infection (Figure S5e,f).^30^ We found that none of the compounds were cytotoxic to host cells, indicating that the observed effect on viral replication was not a result of cell death (Figure 7e).

We also observed that the ability of the virus to recover following a 24-h drug exposure was compromised in **MTDB-imi6** treated samples, but not in samples treated with the control molecules **MTDB** and **TDB-imi6** (Figure S5g). Additionally, we investigated whether the **MTDB** family of compounds could affect the SARS-CoV-2 virions alone by exposing them to these compounds in a cell-free environment (Figure S5h,i). No virucidal effects were observed when cell free virus was incubated with **MTDB-imi6**, **MTDB**, and **TDB-imi6**, providing evidence that **MTDB-imi6** indeed only affects the virus postinfection, when its genomic RNA is exposed to the cellular media and small molecules therein. Finally, we found that **MTDB-imi6** also inhibited the replication of the SARS-CoV-2 Alpha and Delta variants of concern (Figure S5j,k), which was expected given that the pseudoknot is conserved across all the SARS-CoV-2 variants. Overall, the antiviral drug assays show that **MTDB-imi6** exhibits antiviral activity against SARS-CoV-2, is specific against the betacoronaviral three-stemmed pseudoknot, and affects them with an irreversible impact, and thus the PINAD approach could be used in the development of therapeutics against SARS-CoV-2 infection.

### MTDB-Derived PINAD Reduces Viral Burden in Mice Infected with SARS-CoV-2

**Figure 8:**
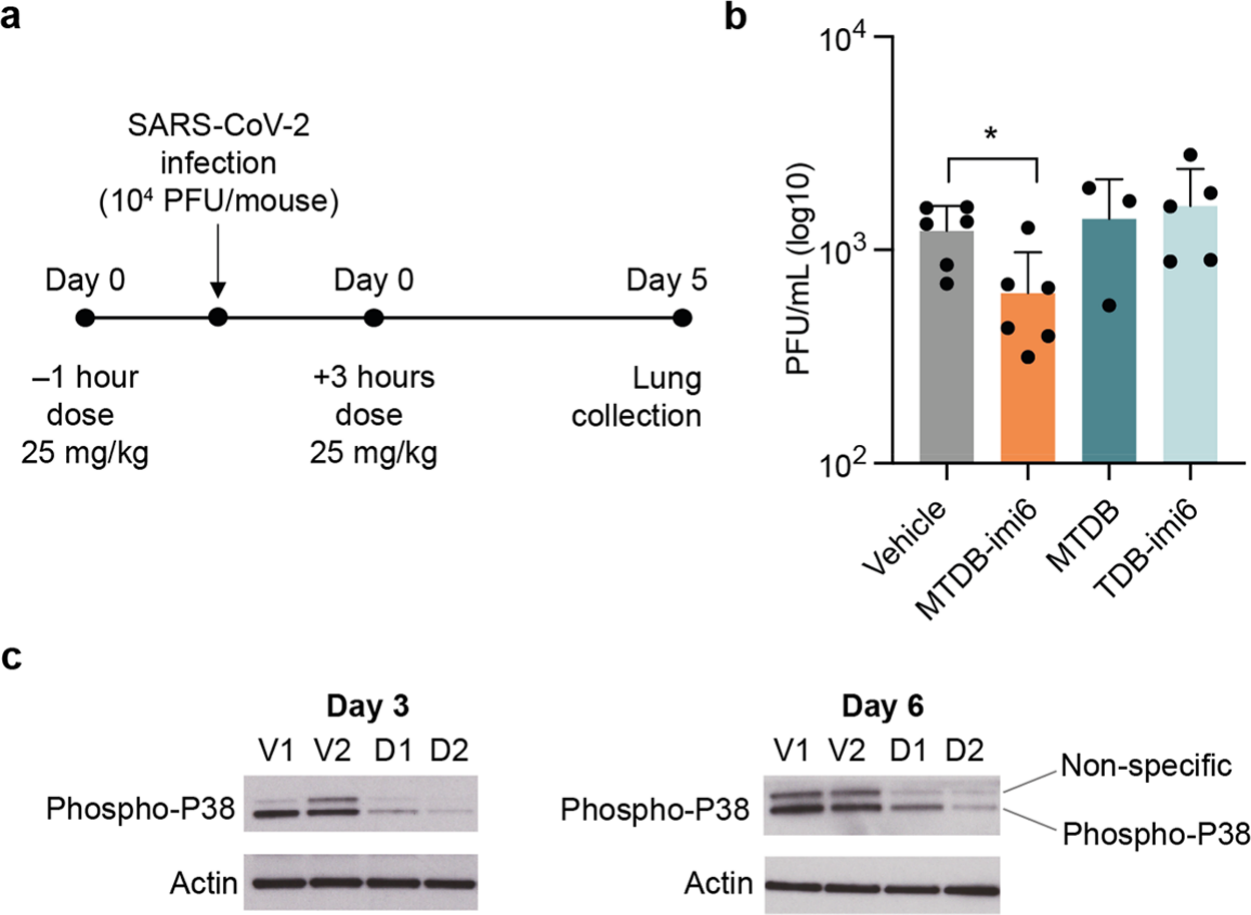
**MTDB-imi6** degrader *in vivo* activity against SARS-CoV-2 infection in K18-hACE2 mice. (a) Eight- to 12-week-old female K18-hACE2-transgenic mice were intranasally infected with 10^4^ plaque-forming units (PFU) of SARS-CoV-2 and treated intranasally 1 h preinfection and 3 h postinfection with **MTDB-imi6** (25 mg/kg) (*n* = 6), **MTDB** (10 mg/kg, maximal dose that could administered given limited solubility) (*n* = 3), **TDB-imi6** (25 mg/kg) (*n* = 5), and vehicle control (*n* = 6). (b) Administration of **MTDB-imi6** leads to a decrease in lung viral load of SARS-CoV-2 infected K18-hACE2 mice. No differences in lung viral load between vehicle control and **MTDB** and **TDB-imi6**-treated mice were observed. Mean ± SD is shown; *p < 0.05; unpaired *t* test. (c) Western blot analysis of phospho-p38 from lung extracts of transgenic K18-hACE2 mice treated with three doses of 10 mg/kg of vehicle (V1, V2) or **MTDB-imi6** (D1, D2) at 1 h before infection and 1 and 2 days postinfection (*n* = 2).

To further test the therapeutic application of the PINAD approach, we evaluated **MTDB-imi6***in vivo* in a SARS-CoV-2 mouse model of infection (transgenic K18-hACE2 mice which express humanized ACE2 receptor, necessary for SARS-CoV-2 to enter cells). Intranasally infected mice were treated 1 h before and 3 h after the infection with one of the molecules from the **MTDB** series (Figure 8a). Animals administered with **MTDB-imi6** (at 25 mg/kg) showed a significant reduction of lung viral load relative to the vehicle control group by plaque assay, unlike the control molecules (Figure 8b). Additionally, we investigated the *in vivo* antiviral potential of either **MTDB-imi6** or vehicle treatments using proteins extracted from the lungs of K18-hACE2 transgenic mice on day 3 or day 6 postinfection (Figure 8c). Reassuringly, we observed that at both time points of infection, the **MTDB-imi6** treated mice showed a strong reduction in the phosphorylated levels of p38, a biomarker of SARS-CoV-2 infection and replication.^30^ Altogether, these findings illustrate that PINADs are compatible with living organisms, with **MTDB-imi6** being able to exert an antiviral effect in mice.

## Conclusions

Here we described proximity-induced nucleic acid degraders (PINADs), a class of bifunctional small molecules which bind and then degrade nucleic acids in a proximity-induced manner. To exemplify, we have designed two series of PINADs against structural elements of the SARS-CoV-2 genome—PDS-degraders which degrade G-quadruplexes and MTDB-degraders which degrade betacoronaviral pseudoknots. We achieved this by functionalizing the parent binder molecules with imidazole moieties, which act as RNA-degrading warheads, attached via long and flexible PEG linkers. We have demonstrated that these molecules bind and degrade their target RNAs *in vitro*, found evidence for target engagement as well as antiviral effects in cellular systems, and have shown that MTDB-PINAD relieves the SARS-CoV-2 burden in mice, which acts as a proof of concept that PINADs are both tolerated and efficacious *in vivo*. All these findings suggest that PINADs have the potential for therapeutic development. Although we have demonstrated utility of this strategy against high abundance transcripts, further work needs to be done to investigate whether PINADs can deplete low abundance and/or short half-life transcripts.

Indeed, targeting RNAs with small molecules can greatly expand the druggable genome. For example, many viral genomes,^31^ mRNAs of disordered proteins,^32^ long noncoding,^33^ and micro-RNAs^34^ are all valid therapeutic targets that cannot be subjected to protein-centered approaches; they can instead be targeted as RNAs. In some cases the RNA binding event alone can result in the desired phenotype.^3^ However, using a PINAD rather than a binder provides a number of advantages: RNA degradation can result in a different, stronger phenotype, whereas the effect of the binder alone might be too weak to influence the phenotype; weak binders can be transformed into potent degraders as exemplified by **MTDB-imi6**; finally, taking advantage of the linker to modulate the solubility of small molecules is especially relevant since many binding pockets in RNA are hydrophobic^35^ and require a hydrophobic binder, which might exhibit poor solubility in aqueous media, as is the case with **MTDB**.

The recent advances in RNA binder screening hint that discovery rate of selective RNA binders is not that different to that of protein binders, which suggests that many more selective RNA binders will be within reach in the near future.^36,37^ Combining high-throughput screening with a PINAD or a different approach to targeted RNA degradation holds potential for a rapid drug discovery platform, the need for which was affirmed during the COVID-19 pandemic. Viruses are capable of rapid mutation as a part of their immune evasion strategy, which greatly complicates antiviral drug discovery campaigns.^15^ The ability to target and degrade highly conserved viral RNA structures, such as the betacoronaviral pseudoknot, can prevent these issues and result in efficient antiviral therapies. Another group of diseases which can be tackled by targeted RNA degraders are pathologies hallmarked by protein misfolding, such as Alzheimer’s disease, Huntington’s disease, or type II diabetes.^38^ These misfolded proteins do not have well-defined structures and thus are extremely difficult to target with small molecules. One way to circumvent this issue is to target mRNAs of corresponding proteins.^32^ Thus, the PINAD approach holds the promise for rapid development of therapeutic modalities against diseases impossible to tackle using well-established methods.

## Data Availability

All data and methods are available in the manuscript or the Supporting Information. All cell lines are available upon request through a material transfer agreement with the University of Cambridge or from the Instituto de Medicina Molecular João Lobo Antunes.

## Supporting Information Available

Supporting figures, methods, synthesis information, NMR spectra (PDF)
Transparent Peer Review report available (PDF)

## Accession Codes

The accession number for Nanopore sequencing data generated in this study is GSE182826.
