# Supplementary material for: Proximity-Induced Nucleic Acid Degrader (PINAD) Approach to Targeted RNA Degradation Using Small Molecules
Source: ACS Cent Sci. 2023 Apr 26;9(5):892–904. doi: 10.1021/acscentsci.3c00015 (PMC10214512; doi:10.1021/acscentsci.3c00015)
Supplement: Supplementary file 1 — oc3c00015_si_001.pdf [file oc3c00015_si_001.pdf]

SUPPORTING INFORMATION

Proximity-Induced Nucleic Acid Degrader (PINAD) Approach to Targeted RNA Degradation Using Small Molecules

Sigitas Mikutis,*#1 Maria Rebelo,#2 Eliza Yankova,#3,4 Muxin Gu,[3] Cong Tang,[2] Ana R. Coelho,[2] Mo Yang,[5] Madoka E. Hazemi,[1] Marta Pires de Miranda,[2] Maria Eleftheriou,[3,4] Max Robertson,[1] George S. Vassiliou,[3] David J. Adams,[6] J. Pedro Simas,[2,7] Francisco Corzana,[8] John S. Schneekloth Jr.,[5] Konstantinos Tzelepis*3,4 & Gonçalo J. L. Bernardes*1,2

[1]Yusuf Hamied Department of Chemistry, University of Cambridge, Lensfield Road, Cambridge CB2 1EW, UK

[2]Instituto de Medicina Molecular João Lobo Antunes, Faculdade de Medicina, Universidade de Lisboa, Avenida Professor Egas Moniz, 1649-028, Lisboa, Portugal

[3]Wellcome-MRC Cambridge Stem Cell Institute, University of Cambridge, Cambridge CB2 0AW, UK

[4]Milner Therapeutics Institute, University of Cambridge, Puddicombe Way, Cambridge CB2 0AW, UK

[5]Chemical Biology Laboratory, Center for Cancer Research, National Cancer Institute, Frederick, Maryland 21702, United States

[6]Experimental Cancer Genetics, Wellcome Trust Sanger Institute, Hinxton, Cambridge, CB10 1SA, UK

[7]Católica Biomedical Research and Católica Medical School, Universidade Católica Portuguesa, 1649-023 Lisboa, Portugal

[8]Departamento de Química, Centro de Investigación en Síntesis Química, Universidad de La Rioja, 26006 Logroño, Spain

#These authors contributed equally to this work.

Correspondence should be addressed to G.J.L.B. or S.M. or K.T.:

E-mail: gb453@cam.ac.uk
E-mail: sm2029@cam.ac.uk
E-mail: kt404@cam.ac.uk

Tables of contents

1. **Supporting Figures** **S3**
2. **Methods** **S8**
3. **Synthesis** **S18**
4. **NMR Spectra** **S30**
5. **References** **S36**

1. Supporting Figures

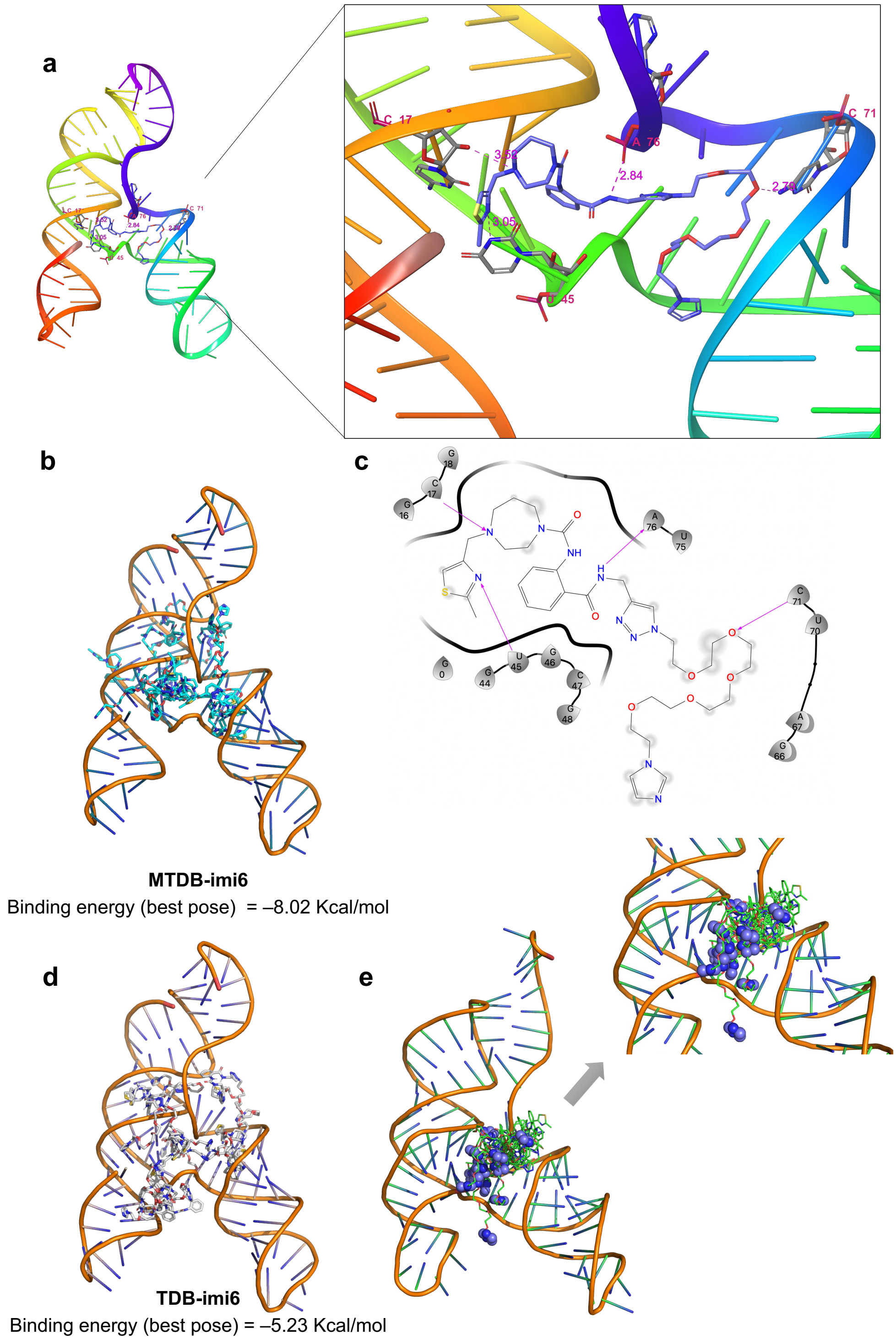

Fig. S1 │**Docking and MD simulations of degraders with pseudoknot. a,** Proposed binding pose (pose #3) of **MTDB-imi6** (stick) in the pseudoknot generated by AUTODOCK 4.2. Putative hydrogen bonds between the ligand and the receptor are also shown. **b**, 10 best scored poses, in terms of binding energy, of **MTDB-imi6** in the pseudoknot generated by AUTODOCK 4.2. **c**, **MTDB-imi6** interaction diagram (hydrogen bonds, pose #3) with the pseudoknot generated with free Maestro 3.0 (https://www.schrodinger.com/). **d**, 10 best scored poses, in terms of binding energy, of **TDB-imi6** in the pseudoknot

generated by AUTODOCK 4.2. **e**, Structural ensemble of 10 frames taken regularly along 0.5 μs MD simulations. Only the first structure of the pseudoknot is shown for clarity. The heavy atoms of the imidazole residue are shown as spheres.

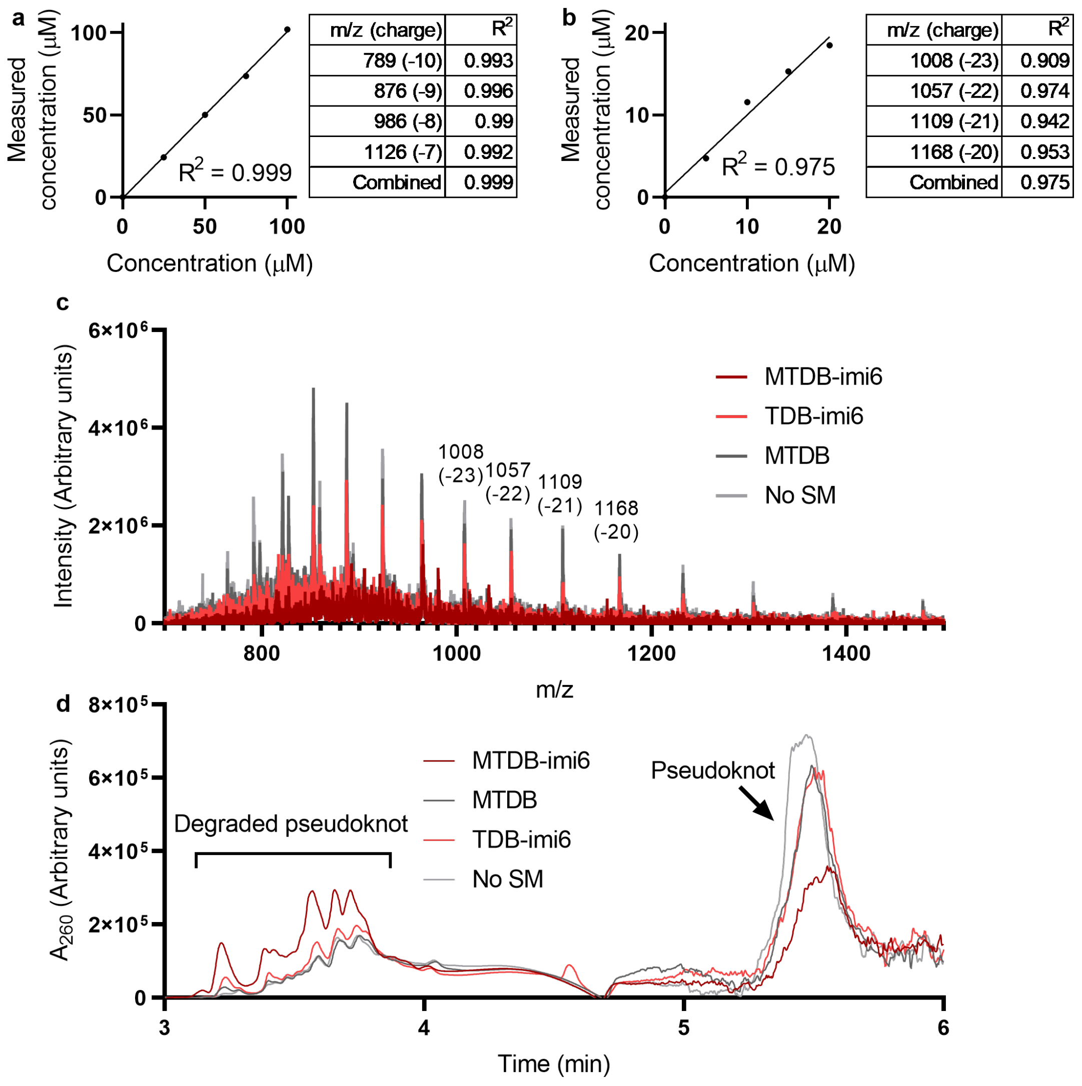

Fig. S2 | Degradation of RNA oligonucleotides can be analyzed using liquid chromatography-mass spectrometry (LC-MS). (a) Calibration curve based on four combined m/z values and R^2 values for individual and combined masses for the rG4-forming NRAS oligonucleotide. (b) Calibration curve based on four combined m/z values and R^2 values for individual and combined masses for the pseudoknot oligonucleotide. (c) An example of ion series corresponding to the pseudoknot after a three-hour incubation with MTDB-imi6 and control molecules. m/z values used to calculate the concentration of the nucleotide are shown. Degradation of the pseudoknot leads to depletion of m/z signals. (d) An example of noise-subtracted A_{260} chromatograms corresponding to the pseudoknot after a three-hour incubation with MTDB-imi66 and control molecules.

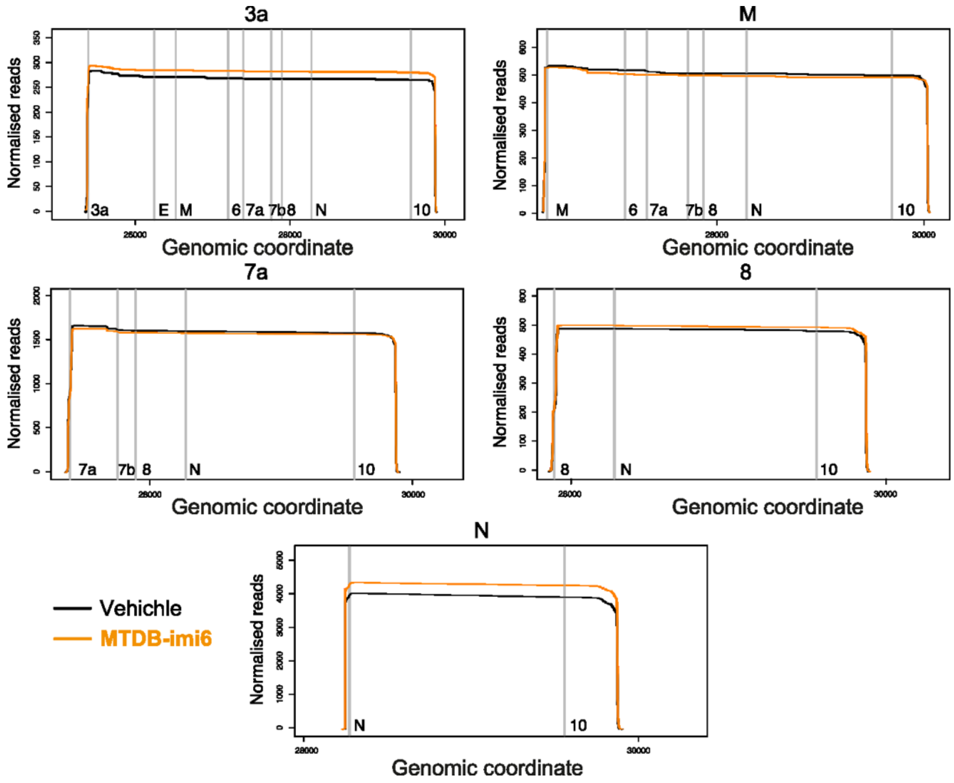

Fig. S3 ∣Treatment with MTDB-imi6 has no effect on the subgenomic SARS-CoV-2 RNAs. Distribution and abundance of aligned reads mapped exclusively on the indicated sub-genomic RNAs for control- or **MTDB-imi6**-treated SARS-CoV-2 RNA, based on alignments with minimap2.

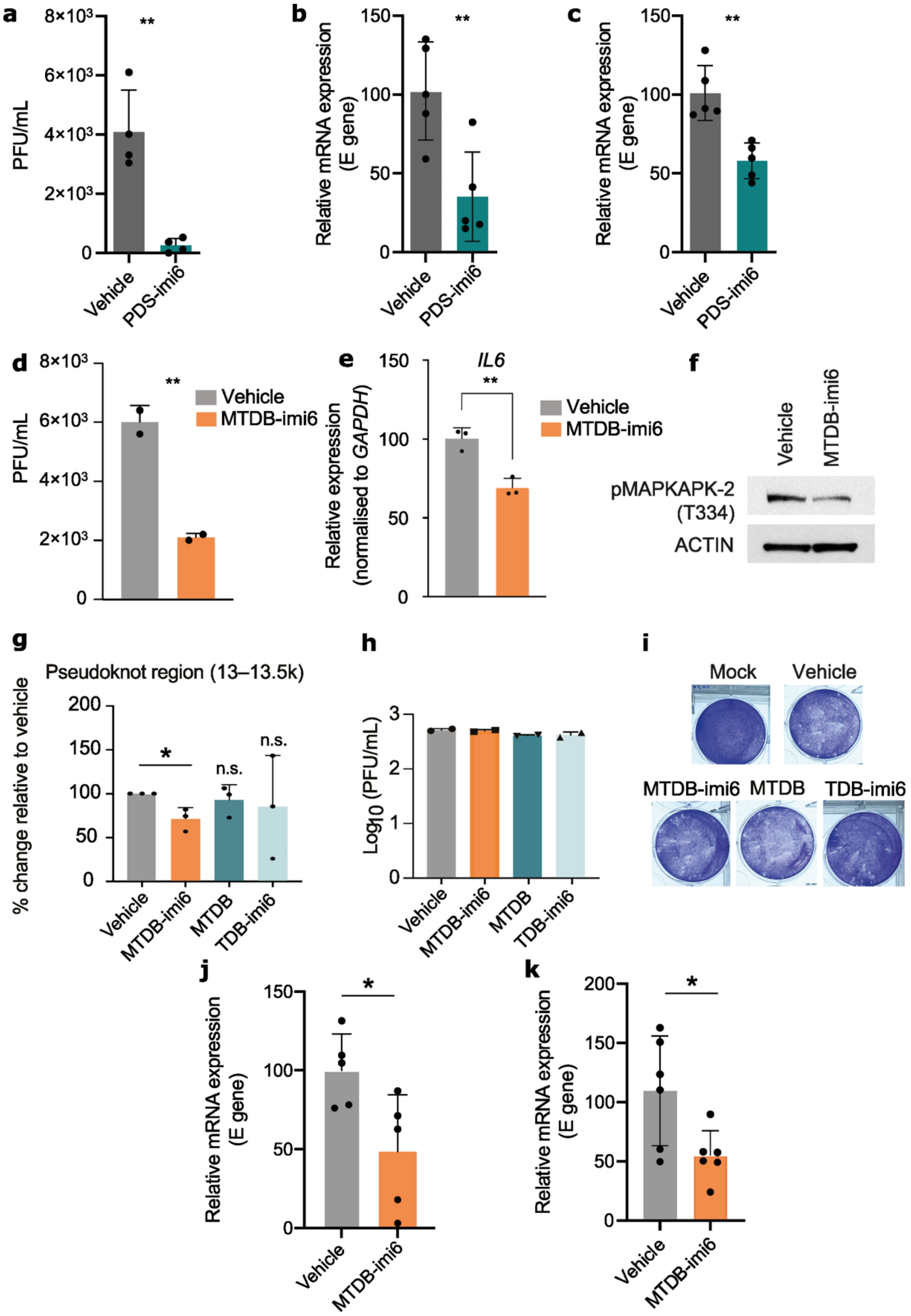

Fig. S4 │Antiviral effects of PINADs. a, Treatment after infection with **PDS-imi6** at 6 μM leads to a significant decrease in infectious virus (PFU/mL), as measured by plaque assay. **b, c,** 24-h treatment with **PDS-imi6** at 6 μM inhibited viral replication of SARS-CoV-2 B.1.1.7 (Alpha) and B.1.617.2 (Delta) variants, respectively. Cells were treated 1 h after infection (n=5). Mean ± SD is shown; * $p < 0.05$; unpaired t-test. **d,** Treatment after infection with **MTDB-imi6** at 6 μM leads to a significant decrease in infectious virus (PFU/mL), as measured by plaque assay. **e,** RT-qPCR validation of *IL6* in SARS-CoV-2 infected VERO CC-L81 cells treated with either 6

μM **MTDB-imi6** or vehicle for 24 h (n = 3). Student's t test. Mean + SD of three independent replicates. **f,** Western blot analysis of phospho-MAPAPK2 (T334) from SARS-CoV-2 infected VERO CCL-81 cells treated with either 6 μM **MTDB-imi6** or vehicle for 24 h. **, p <0.01. **g**, Virus ability to recover after 24 h incubation with **MTDB-imi6** and control molecules **MTDB** and **TDB-imi6**, as determined by qPCR targeting the pseudoknot region. Viral recovery was impaired in **MTDB-imi6** treated samples**,** but not in samples treated with the control molecules **MTDB** and **TDB-imi6**. *p < 0.05, unpaired t-test. **h**, **i**, Virucidal activity was assessed by incubating 1000 PFU of SARS-CoV-2 with compounds at 6 μM, for 1 h at 37 ºC after which residual viral infectivity was determined by plaque assay. **MTDB-imi6**, **MTDB** and **TDB-imi6** showed no virucidal effect on cell-free virions, suggesting that the **MTDB-imi6** antiviral activity is mediated by inhibiting virus replication in host cells and not by inactivation of cell free virions. **j, k,** 24-h treatment with **MTDB-imi6** at 6 μM inhibited viral replication of SARS-CoV-2 B.1.1.7 (Alpha) and B.1.617.2 (Delta) variants, respectively. Cells were treated 1 h after infection (n=5 or 6). Mean ± SD is shown; * p < 0.05; unpaired t-test.

2. Methods

Oligonucleotide preparation for binding studies. To prepare oligonucleotides for binding studies, they were dissolved in an appropriate buffer to a final concentration of 100 nM, then incubated at 95 ºC with shaking for 5 min. They were allowed to cool to room temperature for at least 1 h before further dilutions/treatments to anneal.

Fluorescence quenching assays. Cy5-tagged oligonucleotide corresponding to a G4 structure found on 5'UTR of *NRAS* mRNA (final concentration 50 nM) was dissolved in 20 mM HEPES pH 7.4 buffer supplemented with KCl (100 mM) and $MgCl_2$ (10 mM) and plated on a 96-well plate. The oligonucleotide was treated with various concentrations of PDS family ligands, ranging from 5 nM to 10 μM, or a water vehicle control, followed by 30-minute incubation at 4 ºC. Fluorescence corresponding to Cy5 fluorophore was then measured for each oligonucleotide-small molecule/vehicle control combination using a plate reader (BMG CLARIOstar) at 25.0 ºC. For all the molecules, serial dilutions with a dilution ratio 1:1 were used, with the higher concentration tested being 10 μM. Fluorescence was normalised to a vehicle control and binding curves for each molecule were obtained via a sigmoidal fit by setting the Hill Slope coefficient to 1.

RNA species	Sequence
Cy5 NRAS 5'UTR G4	Cy5-UGUGGGAGGGGCGGGUCUGGGUGC

Microscale Thermophoresis (MST). MST experiments were conducted using a Monolith NT.115 system (NanoTemper Technologies). The 5′-Cy5-labeled SARS-CoV-2 FSE RNA solution was prepared in the folding buffer (50 mM HEPES, pH 7.5, 100 mM KCl, 10 mM $MgCl_2$, 0.005% Tween 20) at 100 nM (2×) and annealed. The small molecules were prepared in the buffer with 2× of designed concentrations (with 10% DMSO). Then, RNA samples were mixed with corresponding small molecule solutions in 1:1 (v/v) ratio, resulting in 50 nM RNA and 5% DMSO. Finally, initial fluorescence scans were carried out with triplicate runs. The dissociation constant was then determined by fitting the concentration-dependent curve (MO.Affinity Analysis

v2.3). For measurements with Click-Degrader 1, a FAM-labelled pseudoknot construct was used instead of Cy5-labeled construct.

RNA species	Sequence
Cy5 pseudoknot	Cy5-UUUGCGGUGUAAGUGCAGCCCGUCUUACACCGUGCGGCACAGGCACUAGUACUGAUGUCGUAUACAGGGCUUUU
FAM pseudoknot	FAM-UUUGCGGUGUAAGUGCAGCCCGUCUUACACCGUGCGGCACAGGCACUAGUACUGAUGUCGUAUACAGGGCUUU

***In vitro* pseudoknot oligo degradation reactions.** RNA oligonucleotide (200 µM for G4, 20 µM for non-labelled pseudoknot or 1 µM for FAM-pseudoknot) was added to a pH 7.5 buffer (20 mM HEPES, 50 mM KCl/LiCl, 10 mM $MgCl_2$, 200 µM $CuSO_4$, 700 µM THPTA and 50 mM NaAsc, for G4 or 20 mM HEPES, 50 mM KCl and 10 mM EDTA for pseudoknot or 50 mM HEPES, 100 mM KCl, 10 mM $MgCl_2$, 1 mM $ZnCl_2$). The mixture was incubated at 37 ºC for 30 min. **PDS** (200 µM) or **MTDB** family degrader (1 mM) was then added. The reaction mixture was incubated at 37 ºC for 3, 4 or 6 h and then kept at 4 ºC, with G4 degradation reaction getting quenched with EDTA (12 mM). The reaction mixtures were analyzed by LC-MS or gel electrophoresis. The sequences of oligonucleotides are given in the table below. Oligomers were synthesized by Integrated DNA Technologies, Inc.

RNA species	Sequence
Pseudoknot	UGCGGUGUAAGUGCAGCCCGUCUUACACCGUGCGGCACAGGCACUAGUACUGAUGUCGUAUACAGGGCU
Pseudoknot control	UGCGGUGUAAGUGCAGCCCGUCUUACACCGUGAAUAUAUGGCACUAGUACUGAUGUCGUAUACAGGGCU
NRAS 5’UTR G4	UGUGGGAGGGGCGGGUCUGGGUGC
NRAS 5’UTR control	UGUAGAAAGAGCAGAUCUAGAUGC

LC-MS analysis of oligonucleotides. Oligonucleotides were analyzed by LC-MS as described previously[1]. Briefly, oligomers were analyzed using a Xevo G2-S TOF mass spectrometer coupled to an Acquity UPLC system using an Acquity UPLC BEH C18 1.7 µm column. Two mobile phases were used: 16.3 mM TEA, 400 mM HFIP in H_2O and 16.3 mM TEA, 400 mM HFIP in 80:20 v/v MeCN and H_2O, with a flow rate of 0.200 mL/min. Calibration curves for the RNA species were made based on intensities of negative m/z signals. Intensity of each peak in ion series was calculated and normalized to the concentration. A linear fit of injected concentration versus measured concentration was made. Four neighbouring peaks with the best R^2 values (e.g., the best fit to the linear model, see Fig. S2a, b) were used to calculate relative concentrations in oligonucleotide degradation reactions, by taking the average measured concentration of the four m/z values. Intensities of integrated peaks were calculated using native modules of KNIME software (version 3.6.1) platform. Deconvoluted mass spectra were reconstructed from the ion series using the MaxEnt algorithm on MassLynx software (v. 4.1 from Waters).

Polyacrylamide gel electrophoresis analysis. Gel polyacrylamide electrophoresis was carried out as described before[1]. Briefly, RNA oligomer mixture was mixed in 1:1 ratio with a loading buffer (95% formamide, 0.025% SDS, 0.025% bromophenol blue (BPB), 0.025% xylene cyanol FF, 0.025% ethidium bromide, 0.5 mM EDTA), heated at 70 °C for 5 min, and cooled to 0 °C. Polyacrylamide gel electrophoresis was performed on NovexTM TBE-Urea Gels (Thermo Fisher), containing 15% polyacrylamide or on NovexTM TBE Gels (Thermo Fisher), containing 20% polyacrylamide under 1× TBE buffer (89 mM Tris, 89 mM boric acid, 2 mM EDTA) at 180 V for 50 min. The fluorophore-labelled RNA was visualized with ChemiDoc MP (Bio-Rad, United Kingdom).

Docking studies with AUTODOCK 4.2. AUTODOCK 4.2 was used to predict the region where **MTDB-imi6** and **TDB-imi6** bind to the cryo-EM structure of the pseudoknot (PD ID: 6XRZ). Standard settings for *autogrid* (gridcenter: 71.068 69.058 67.17; number of grid points in xyz: 126, 126, 126; spacing (Å) = 0.375) and *autodock* (genetic algorithm, max. number of evaluations = 25000, output=Lamarckian GA(4.2)) were selected with *AutoDockTools* 1.5.6.

MD simulations. Simulations on **MTDB-imi6** bound to the pseudoknot (pose #3 obtained by docking calculations) were performed with AMBER 20 package[2], implemented with RNA.OL3[3] and GAFF2[4] force fields. The setup for the molecular dynamics was similar to that described previously[5], except for the time of the production step that was set to 500 ns instead of 200 ns.

Cell culture. VERO CCL-81 cells were maintained in Dulbecco's Modified Eagle Medium (DMEM) medium supplemented with 10% fetal bovine serum (FBS), 1% penicillin-streptomycin and 1% Glutamax at 37 °C and 5% CO_2. HEK293FT cells were cultured in DMEM with L-glutamine, supplemented with 10% FBS and 1% penicillin/streptomycin. MOLM13 cells were maintained in RPMI-1640 with L-glutamine (Gibco), supplemented with 1% penicillin/streptomycin at 37 °C and 5% CO_2.

Viral stocks. SARS-CoV-2 stocks used to infect VERO CCL-81 cells were established from passage 4 of SARS-CoV-2 isolated from a Portuguese patient (internal reference: 606_IMM ID_5452) at approximately $1.7x10^6$ PFU/mL, after 4 days in VERO CCL-81 culture. Stock titers were calculated by plaque assay, as described below.

Viral infection of VERO CCL-81. VERO CCL-81 cells at 80% confluency were incubated with SARS-CoV-2 inoculum for 1 h at 37 ºC. After incubation, the inoculum was removed and DMEM medium supplemented with 2.5% FBS, 1% penicillin-streptomycin and 1% glutamax was added for 24 h, or until samples were harvested.

Virus concentration and viral RNA extraction. VERO CCL-81 cells at 80% confluency were incubated with SARS-CoV-2 inoculum for 1 h at 37 ºC, then the inoculum was removed and DMEM medium supplemented with 2.5% FBS, 1% penicillin-streptomycin and 1% glutamax was added. After 4 days incubation, supernatant was collected and centrifuged at 6,000 g at 4 ºC for 10 h. Then the supernatant was removed and 600 µL RLT buffer was added to resuspend the virus particles. The viral RNA was then extracted using a RNeasy MINI Kit (QIAGEN), following the manufacturer's instructions.

RNA extraction

Cells from viral cultures were resuspended in RLT buffer and extracted using RNeasy Mini Kit (QIAGEN). For *in vitro* assays, SARS-CoV-2 supernatant was concentrated as described above and extracted using RNeasy Mini Kit (QIAGEN), following the manufacturer's instructions. Total RNA from HEK293FT cells for use in *in vitro* assays was extracted using QIAZOL Reagents (QIAGEN), following the manufacturer's protocol.

Agarose gel electrophoresis analysis. 500 ng of SARS-CoV-2 RNA, or total RNA from HEK293FT cells, was incubated with 50 μM of TDB-imi6, MTDB or **MTDB-imi6** in 1x HEPES buffer for 2 h at 37ºC with mild agitation. The samples were then analyzed on a 1.5% agarose gel.

Nanopore sequencing. 500 ng of SARS-CoV-2 RNA was incubated with or without 50 μM of **MTDB-imi6** in 1x HEPES buffer for 2 h at 37 ºC with mild agitation. The samples were then prepared for sequencing following the manufacturer's instructions for Direct RNA Sequencing [SQK-RNA002, Oxford Nanopore Technologies (ONT)]. The prepared libraries were loaded on FLO-MIN106D flow cells (ONT) and sequenced on a MinION Mk1C device (ONT).

Genomic sequence of the Wuhan-hu1 strain of SARS-CoV-2 (GenBank: MN908947.3) and the genomic annotation (NC_045512.2) were downloaded from the NCBI database. Sequence reads were aligned to the Wuhan-hu1 genome using minimap2[6] with parameters "-ax splice -N32 -un -k13". CIGAR strings of the alignments were processed by customized scripts. Reads were flagged as leader if the splice junction within the read starts between the first 60-120 bp of the genome. Reads were assigned to individual transcript if it covers more than 90% of the annotated transcript of more than 90% or the read sequence lies within the transcript.

Drug assay to determine 50% inhibitory concentration. Increasing concentrations of **MTDB-imi6** (ranging from 0.07 to 18 μM) were tested to determine the 50% inhibitory concentration (IC_{50}). Vehicle (H_2O) treatment and control molecules were included in parallel. Cells were seeded in 96 well-plates at approximately 40%

confluency 24 h before infection. **MTDB-imi6**, **MTDB** or **TDB-imi6** were added either 1 h before infection or 1 h after infection. SARS-CoV-2 cryopreserved stocks, were thawed at room temperature and used to infect cells at a 0.05 multiplicity of infection (MOI). Inhibition of viral growth was measured by harvesting cells at 24 h post-infection. Viral growth was assessed by measuring viral loads by PCR targeting the E gene and pseudoknot region.

Inhibitory effect against SARS-CoV-2 Alpha variant (lineage B.1.1.7) and Delta variant (lineage B.1.617.2)
SARS-CoV-2 (Alpha) variant (lineage B.1.1.7) was obtained through BEI Resources, NIAID, NIH: SARS-Related Coronavirus 2, Isolate hCoV-19/England/204820464/2020, NR-54000, contributed by Bassam Hallis. The delta variant (NR-55611; lineage B.1.617.2; Isolate hCoV-19/USA/PHC658/2021) was obtained through BEI Resources, NIAID, NIH, contributed by Dr. Richard Webby and Dr. Anami Patel. VERO CCL-81 were infected with SARS-CoV-2 variants, at a 0.05 MOI, 1 h before treatment with **MTDB-imi6** at 6 μM. 24 h post-treatment cells were harvested and inhibition of viral growth was measured by RT-qPCR targeting the E gene.

Detection of viral plaque-forming units by plaque assay. Approximately 8 x 10^5 VERO CCL-81 cells per well were seeded in 6-well plates and allowed to grow to 80% confluence for 24 h. Viral stocks or supernatant of viral cultures treated with the compounds were diluted in DMEM medium supplemented with 2.5% FBS, 1% penicillin-streptomycin and 1% Glutamax, and added to pre-seeded wells of a 6-well plate and incubated for 1 h at 37 ºC. Plates were rocked manually to redistribute inoculum every 15 min. After removal of the inoculum, cells were overlaid with 1.25% CMC in supplemented DMEM and incubated at 37 ºC for 4 days. After incubation, the CMC overlay was removed, and cells were fixed with 4% formaldehyde/PBS and stained with 0.1% toluidine blue. Viral plaques were counted to determine infectious titers [PFU (plaque forming units)/mL].

Quantification of viral load by RT-qPCR. 500 ng of RNA was converted to cDNA using SuperScript™ VILO™ Master Mix. The levels of genomic and sub-genomic

SARS-CoV-2 transcripts were analysed on a QuantStudio™ 5 real-time PCR machine (Applied Biosystems) using PowerUp™ SYBR™ Green Master Mix (Applied Biosciences) according to the manufacturer's instructions. All samples, including the template controls, were assayed in triplicates. The relative quantification of target gene expression was performed using the comparative cycle threshold (C_T) method. The primer sequences are listed in the table below.

Gene	Forward	Reverse	Ref.
SARS-CoV-2-E gene	ACAGGTACGTTAATAGTTAATAGCGT	ATATTGCAGCAGTACGCACACA	[7]
SARS-CoV-2-N gene	GACCCCAAAATCAGCGAAAT	TCTGGTTACTGCCAGTTGAATCTG	[8]
SARS-CoV-2 – 3k-4.5k	GGAGGAGGTGTTGCAGGAGCCT	TGGGCCGACAACATGAAGACAGT	
SARS-CoV-2 – 6k-8k	AGTCAGAGGACGCGCAGGGAAT	TCTGTGTGGCCAACCTCTTCTGT	
SARS-CoV-2 – 10k-10.5k	ACCTCAGCTGTTTTGCAGAGTGGT	ACGTCATCAAGCCAAAGACCGT	
SARS-CoV-2 – 11.5k-12k	TGGGTGTTGGTGGCAAACCTTGT	ACTGGACACATTGAGCCCACAA	
SARS-CoV-2 – 12k-12,5k	TGCTTTCCATGCAGGGTGCTGT	AGCAACAGCCTGCTCATAAGCT	
SARS-CoV-2 – 12,5k-13k	TGCATCAGCATTGTGGGAAATCCA	ACCGGCAGCACAAGACATCTGT	
SARS-CoV-2 – 13k-13.5k	CCGCGAACCCATGCTTCAGTCA	CACGGTGTAAGACGGGCTGCAC	
SARS-CoV-2 – 13.5k-14k	ACACAATGGCAGACCTCGTCTATGC	AGCTTGGCGTACACGTTCACCT	
SARS-CoV-2 – 14k-14.5k	TGTGATGCCATGCGAAATGCTGGT	GGAACTCCACTACCTGGCGTGG	

SARS-CoV-2 – 20k-21.5k	AGCTCTTGGAGGTTCCGTGGCT	GGCTGTCCACCATGCGAAGTGT	
TRS-forward	CAAACCAACCAACTTTCGATCTCTTGTA		9
SARS-CoV-2 – S gene		TTGTCAGGGTAATAAACACCACGT	
SARS-CoV-2 – Orf8		CGCCGTCAGGACAAGCAAAAGC	
IL-6	TACATCCTCGACGGCATCTC	ACCAGGCAAGTCTCCTCATTG	
18S	GTAACCCGTTGAACCCCATT	CCATCCAATCGGTAGTAGCG	
TBP	ACGAACCACGGCACTGATTT	ACTTCACATCACAGCTCCCC	
SYNCRIP	ACAGAGGAAGTATGGAGGACCA	ATCTCAGTGCCAACAGAAGGC	

Viral recovery assay. Two sets of samples were prepared for the recovery assay, where cells at 80% confluency were infected with SARS-CoV-2 cryopreserved stocks at a 0.05 MOI for 2 h. Then, the inoculum was removed, and infected cells were incubated with the **MTDB-imi6**, **MTDB**, and **TDB-imi6** at 6 μM for 24 h, at 37 ºC and 5% CO_2. After 24 h, in one set of samples cells were harvested into lysis buffer for RT-qPCR analysis of viral growth. On the other set of samples, compounds in supernatant were removed, replaced by drug-free media and incubated for an additional 24 h, at 37 ºC and 5% CO_2. After 24 h of incubation (corresponding to 48 h timepoint) cells were harvested into lysis buffer and viral growth was be measured by RT-qPCR targeting the E gene and/or pseudoknot region. Percentage of viral recovery was normalised to vehicle treated samples.

Cytotoxicity assay. To determine if the compounds were toxic to cells, 1 x 10^4 VERO CCL-81 cells per well were seeded in 96-well plates. After 24 h, cells were incubated with increasing concentrations of **MTDB-imi6**, **MTDB** or **TDB-imi6** (ranging from 0.05 μM to 25 μM) for 24 h. The cellular viability was assessed using CellTiter Blue viability assay (Promega) in accordance with manufacturer's protocol.

Western Blot analysis. For *in vitro* experiments, samples were treated with vehicle (H_2O) or **MTDB-imi6** (6 mM) for 24 h. Cells were then lysed using whole cell lysis buffer (50 mM Tris-HCl pH = 8.0, 450 mM NaCl, 0.1% NP-40, 1 mM EDTA), supplemented with 1 mM DTT, protease inhibitors (Sigma), and phosphatase inhibitors (Sigma). For *in vivo* experiments, the whole left lung from mice was homogenized in 3 mL of DMEM and 750 µL was transferred to an equal volume of whole cell lysis buffer, supplemented as above. Protein concentrations were accessed using Bradford Assays (BioRad). Prior to loading the samples were supplemented with LDS Loading Buffer (Life technologies) and Sample Reducing Agent (Life Technologies). 40 µg of protein was separated on SDS-PAGE gels and blotted onto polyvinylidene difluoride (PVDF) membranes (GE Healthcare). Western Blot experiments were performed using the following antibodies: anti-beta actin (Abcam, ab8224), anti phospho-MAPKAPK-2 (Thr334) (27B7) (Cell Signalling, 3007), anti-Phospho-p38 MAPK (Thr180/Tyr182) (D3F9) XP® (Cell Signalling, 4511), goat anti-mouse IgG H&L (HRP) (Abcam, ab205719) and goat-anti rabbit HRP (Abcam, ab6721).

Antiviral activity in an animal model of SARS-CoV-2 infection. All animal experiments were conducted in the BSL-3 Facility in strict compliance with the relevant EU and national legislation and were approved by the Portuguese official veterinary department for welfare licensing – *Direção Geral de Alimentação e Veterinária* – (license number 01878/2021) and the Instituto de Medicina Molecular Animal Ethics Committee. Eight to twelve-week-old female specific pathogen-free hemizygous for Tg(K18-ACE2)2Prlmn mice (Strain B6.Cg-Tg(K18-ACE2)2Prlmn/J, C57BL/6 genetic background the Jackson laboratory strain 034860) were used in this study. Mice were intranasally infected with 1 x 10^4 PFU of SARS-CoV-2 in 50 μL of DMEM medium (supplemented with 2.5% FBS, 1% penicillin-streptomycin and 1% Glutamax). To investigate the in vivo efficacy of **MTDB-imi6**, mice were administrated intranasally 1 h pre-infection and 3 h post-infection either with vehicle (n=6), **MTDB-imi6** at 25 mg/kg (n=6), **MTDB** at 10 mg/kg (n=3) or **TDB-imi6** at 25 mg/kg (n=5). On day 5 post SARS-CoV-2 infection, animals were humanely euthanized and left lung was harvested for viral quantification by plaque assay. To further investigate the *in vivo* antiviral potential of **MTDB-imi6** proteins for western blot analysis were extracted, on day 3 and day 6

post-SARS-CoV-2 infection, from the lungs of mice that were administered with three doses of **MTDB-imi6** (10 mg/kg) or vehicle, at 1 h before infection and 1 and 2 days after infection.

3. Synthesis

Scheme 1 | Synthetic route to MTDB-imi6.

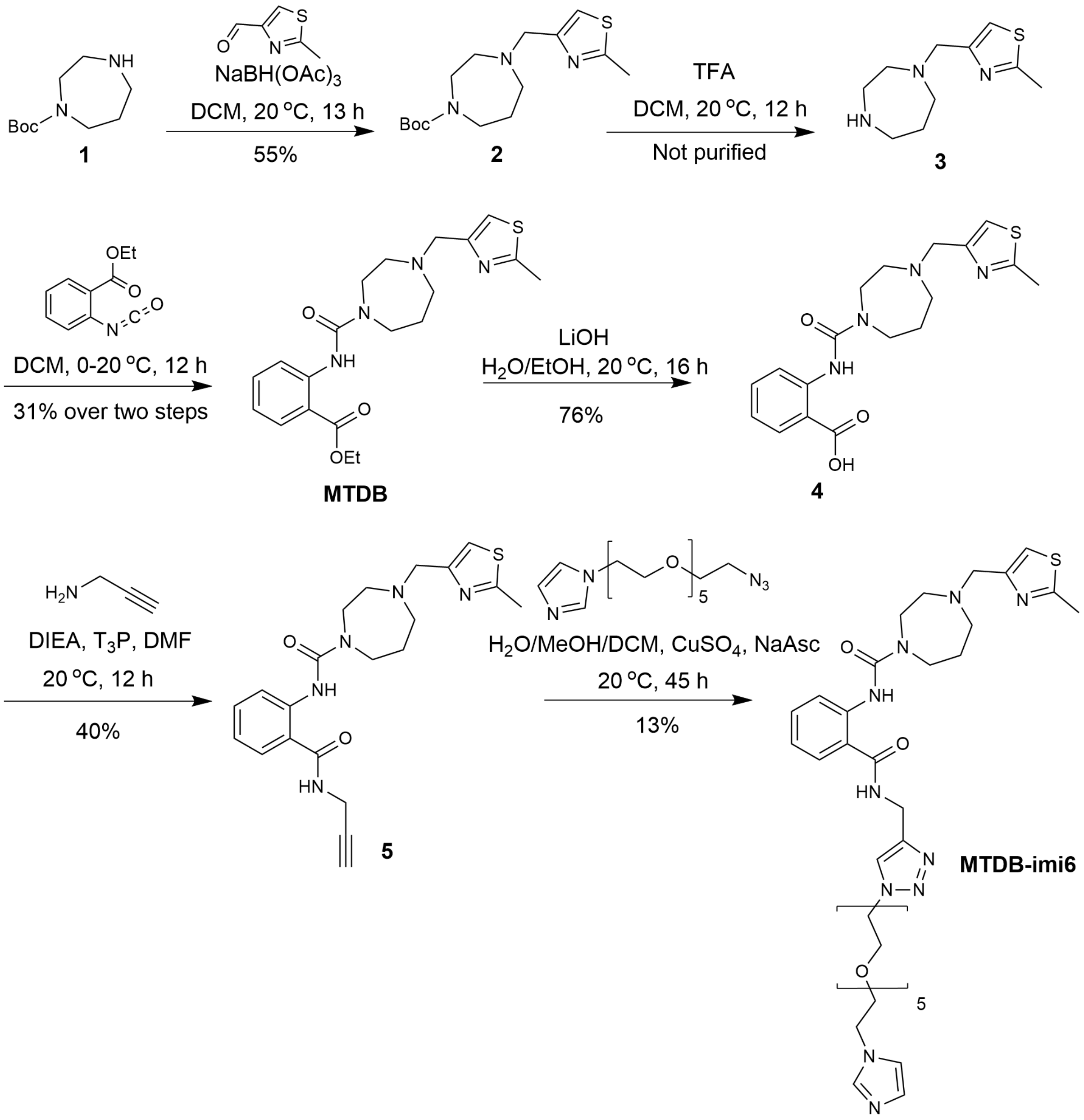

General procedure for preparation of Click-Degrader 1

Click-Degrader 1 was prepared as described in the literature[1].

General procedure for preparation of compound 2

2-methylthiazole-4-carbaldehyde (10.0 g, 78.6 mmol, 1.0 *eq*) in DCM (100 mL) was added to compound **1** (16.5 g, 82.6 mmol, 16.2 mL, 1.1 *eq*) in one portion at 25 ºC under N_2. The mixture was stirred at 25 ºC for 3 h. The mixture was added $NaBH(OAc)_3$ (25.0 g, 118 mmol, 1.5 *eq*) and stirred for 10 h. The residue was poured into water (50 mL) and stirred for 10 min. The aqueous phase was extracted with DCM (3 x 20 mL). The combined organic phase was dried with anhydrous Na_2SO_4 and filtered; the solvent was removed *in vacuo*. The product was purified via column chromatography (gradient, petroleum ether to petroleum ether/ethyl acetate **10**/1) to yield compound **2** (13.5 g, 43.4 mmol, 55% yield) as a yellow oil.

LC-MS [+ scan]: calculated m/z $C_{20}H_{26}N_3O_3S$ 388.2; observed 388.1.

General procedure for preparation of compound 3

TFA (40.0 g, 351 mmol, 26 mL, 8.4 *eq*) was added to compound **2** (13.0 g, 41.7 mmol, 1.0 *eq*) in DCM (130 mL) at 25 ºC under N_2. The mixture was stirred for 12 h. The solvent was removed *in vacuo* to give a TFA salt of compound **7** (23.0 g, crude) as a red oil.

LC-MS [+ scan]: calculated m/z $C_{10}H_{18}N_3S$ 212.1; observed 212.0.

General procedure for preparation of MTDB

To a mixture of compound **3** in DCM (200 mL) was added TEA (9.21 g, 91.0 mmol, 12.7 mL) at 20 ºC under N_2. Then ethyl 2-isocyanatobenzoate (8.70 g, 45.5 mmol) was added to the mixture at 0 °C. The mixture was stirred at 20 ºC for 12 h. The solvent was removed *in vacuo*. The residue was purified by column chromatography (gradient, petroleum ether/ethyl acetate 100/1 to 20/1) to give **MTDB** (5.62 g, 14.0 mmol, 31% yield over two steps) as an off-white solid.

^{1}H NMR (400 MHz, CD_3OD): δ 8.42 (br d, J = 8.4 Hz, 1 H), 8.08 (br d, J = 8.0 Hz, 1 H), 7.62 (s, 1 H), 7.52 - 7.59 (m, 1 H), 7.09 (br t, J = 7.6 Hz, 1 H), 4.34 - 4.46 (m, 4 H), 3.93 (br s, 2 H), 3.77 (br t, J = 6.0 Hz, 2 H), 3.49 (br s, 4 H), 3.28 - 3.31 (m, 1 H), 2.74 (s, 3 H), 2.31 (br d, J = 4.8 Hz, 2 H), 1.43 (t, J = 7.2 Hz, 3 H).

LC-MS [+ scan]: calculated m/z $C_{20}H_{27}N_4O_3S$ 403.2; observed 403.1.

General procedure for preparation of compound 4

To a mixture of compound **MTDB** (5.60 g, 13.9 mmol, 1.0 *eq*) in EtOH (120 mL) and H_2O (30 mL) was added LiOH monohydrate (2.34 g, 55.7 mmol, 4.0 *eq*) at 25 ºC under N_2. The mixture was stirred at 25 ºC for 12 h. The mixture was adjusted to pH = 6 with 1 M HCl and the aqueous phase was extracted with ethyl acetate (3 x 40 mL). Then the organic phase was dried with anhydrous Na_2SO_4, filtered and concentrate *in vacuo* to give compound **4** (2.60 g, 6.94 mmol) as a yellow solid.

LC-MS [+ scan]: calculated m/z $C_{18}H_{23}N_4O_3S$ 375.1; observed 375.1.

General procedure for preparation of compound 5

To a mixture of compound **4** (2.60 g, 6.94 mmol, 1.0 *eq*) and propargyl amine (1.15 g, 20.8 mmol, 1.33 mL, 3.0 *eq*) in DMF (200 mL) was added DIEA (4.49 g, 34.7 mmol, 6.0 mL, 5.00 *eq*) at 25 ºC under N_2. The mixture was added T_3P (4.42 g, 13.9 mmol, 4.1 mL, 2.0 *eq*) and stirred at 50 ºC for 12 h. The mixture was poured into water (200 mL) and the aqueous phase was extracted with ethyl acetate (3 x 70 mL). Then the organic phase was washed with brine (60 mL). The organic phase was dried with anhydrous Na_2SO_4, filtered and the solvent was removed *in vacuo*. The residue was purified by column chromatography (gradient, petroleum ether/ethyl acetate 100/1 to ethyl acetate) to yield **5** (1.20 g, 2.80 mmol, 40% yield).

^{1}H NMR (400 MHz, $CDCl_3$): δ 10.54 (br s, 1 H), 8.46 (d, J = 8.2 Hz, 1 H), 7.43 - 7.51 (m, 2 H), 7.11 - 7.11 (m, 1 H), 6.99 (q, J = 7.6 Hz, 2 H), 6.49 (br s, 1 H), 4.22 (dd, J = 5.2, 2.6 Hz, 2 H), 3.72 - 3.83 (m, 4 H), 3.64 - 3.69 (m, 2 H), 2.77 - 2.96 (m, 4 H), 2.72 (s, 3 H), 2.32 (t, J = 2.6 Hz, 1 H), 2.04 (br d, J = 15.2 Hz, 2 H).

LC-MS [+ scan]: calculated m/z $C_{21}H_{26}N_5O_2S$ 412.2; observed 412.0.

General procedure for preparation of MTDB-imi6

A mixture of Click-Degrader **1** (200 mg, 280 umol, 1.0 *eq*), compound **5** (115 mg, 280 μmol, 1.0 *eq*) and $CuSO_4$ (22.3 mg, 140 μmol, 21.5 μL, 0.5 *eq*) in DCM (5 mL), MeOH (5 mL) and H_2O (5 mL) were stirred at 20 ºC for 30 min. Then NaAsc (11.1 mg, 55.9 μmol, 0.2 *eq*) was added to the mixture and stirred at 20 ºC for 4.5 h. The mixture was diluted with H_2O (10 mL) and then extracted with DCM (3 x 10 mL). The combined organic phase was dried over anhydrous Na_2SO_4, filtered and concentrated *in vacuo*. The residue was purified by via HPLC (column: Phenomenex Gemini-NX 80 x 40 mm x 3 μm; mobile phase: [water (10 mM NH_4HCO_3) - ACN]; B%: 15% - 35%, 8 min) to give **MTDB-imi6** (28.0 mg, 34.9 μmol, 13% yield) as a light yellow oil.

^{1}H NMR (400 MHz, DMSO-d_6): δ 11.03 (s, 1 H), 9.29 (br t, J = 5.2 Hz, 1 H), 8.37 (d, J = 8.4 Hz, 1 H), 7.96 (s, 1 H), 7.74 (br d, J = 7.2 Hz, 1 H), 7.63 (br s, 1 H), 7.43 (t, J = 7.60 Hz, 1 H), 7.15 - 7.31 (m, 2 H), 6.99 (t, J = 7.6 Hz, 1 H), 6.89 (br s, 1 H), 4.46 - 4.53 (m, 4 H), 4.10 (br t, J = 5.2 Hz, 2 H), 3.77 - 3.81 (m, 2 H), 3.71 (br s, 2 H), 3.66 (br t, J = 5.07 Hz, 2 H), 3.44 - 3.57 (m, 20 H), 3.34 (s, 25 H), 2.73 (br s, 1 H), 2.62 (br s, 6 H), 1.84 (br s, 2 H).

Scheme 2 | Synthetic route to TDB-imi6.

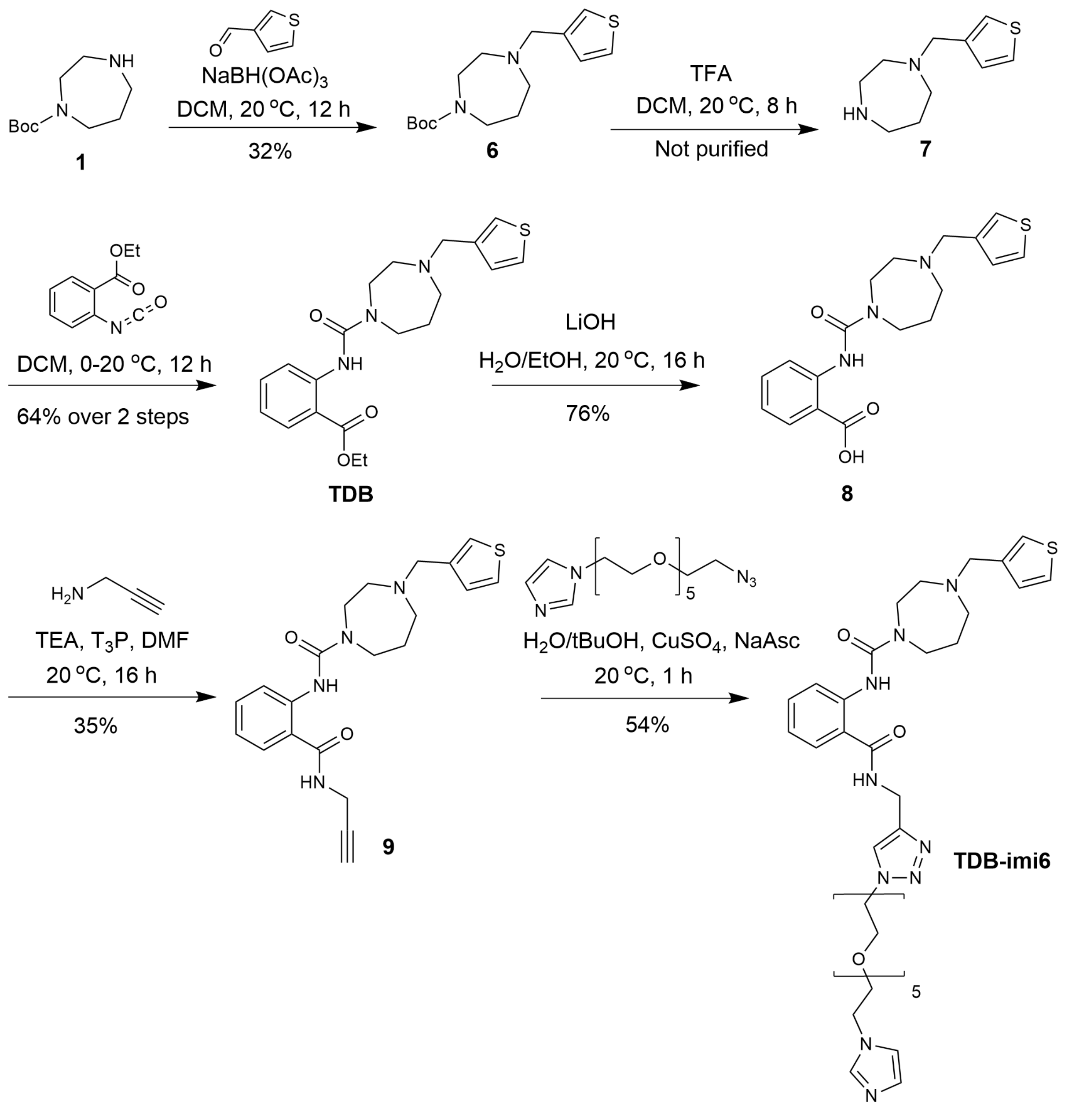

General procedure for preparation of compound 6

To thiophene-3-carbaldehyde (2.00 g, 17.8 mmol, 1.63 mL, 1.0 *eq*) in DCM (80 mL) was added compound **1** (3.93 g, 19.6 mmol, 3.8 mL, 1.1 *eq*) at 20 ºC under N_2. The mixture was stirred at 20 ºC for 3 h. Then $NaBH(OAc)_3$ (5.67 g, 26.8 mmol, 1.5 *eq*) was added to the mixture at 0 ºC and stirred at 20 ºC for 10 h. The reaction mixture was quenched by addition of water (60 mL) and extracted with DCM (2 x 20 mL). The combined organic layers were washed with brine (30 mL), dried over anhydrous

Na_2SO_4, filtered and concentrated *in vacuo*. The residue was purified by silica gel chromatography (gradient petroleum ether/ethyl acetate = 100/1 to 20/1) to give compound **6** (1.70 g, 5.73 mmol, 32% yield) as a red oil.

^{1}H NMR (400 MHz, $CDCl_3$): δ 7.21 - 7.31 (m, 1 H), 7.03 - 7.15 (m, 2 H), 3.64 (s, 2 H), 3.37 - 3.54 (m, 4 H), 2.55 - 2.68 (m, 4 H), 1.81 (br dd, J = 10.8, 4.9 Hz, 2 H), 1.40 - 1.52 (m, 9 H).

General procedure for preparation of compound 7

To a mixture of compound **6** (1.70 g, 5.73 mmol, 1.0 *eq*) in DCM (20 mL) was added TFA (6.16 g, 54.0 mmol, 4.0 mL, 9.4 *eq*) at 20 ºC under N_2. The mixture was stirred at 20 ºC for 8 h. The solvent was removed *in vacuo* to yield a TFA salt of compound **7** (3.00 g, crude) as a red oil which was used in the next step without further purification.

General procedure for preparation of TDB

To a mixture of compound **7** (10.0 g) in DCM (40 mL) was added TEA (6.53 g, 64.3 mmol, 9.0 mL) at 20 ºC under N_2. Then ethyl 2-isocyanatobenzoate (6.17 g, 32.2 mmol) was added to the mixture at 0 ºC. The mixture was stirred at 20 ºC for 12 h. The solvent was removed *in vacuo*. The residue was purified by column chromatography (gradient, Petroleum ether/Ethyl acetate = 100/1 to 20/1) to give **TDB** (4.70 g, 12.1 mmol, 64 % yield over two steps) as an off-white solid.

^{1}H NMR (400 MHz, $CDCl_3$): δ 10.59 (s, 1 H), 8.52 (d, J = 8.4 Hz, 1 H), 7.94 (dd, J = 8.0, 1.53 Hz, 1 H), 7.42 (t, J = 7.6 Hz, 1 H), 7.14 - 7.24 (m, 1 H), 6.96 - 7.07 (m, 2 H), 6.88 (t, J = 7.2 Hz, 1 H), 4.28 (q, J = 7.2 Hz, 2 H), 3.55 - 3.67 (m, 6 H), 2.70 (br s, 2 H), 2.53 - 2.65 (m, 2 H), 1.91 (br s, 2 H), 1.33 (t, J = 7.2 Hz, 3 H).

LC-MS [+ scan]: calculated m/z $C_{20}H_{26}N_3O_3S$ 388.2; observed 388.1.

General procedure for preparation of compound 8

To a mixture of compound **TDB** (100 mg, 0.26 mmol, 1.0 eq) in EtOH (1.2 mL) and H_2O (1.2 mL) was added LiOH monohydrate (65 g, 1.5 mmol, 6.0 eq) at 25 ºC under N_2. The mixture was stirred at 25 ºC for 16 h, after which additional LiOH monohydrate

(130 g, 3.1 mmol, 12.0 *eq*) was added. After 2 h, the mixture was adjusted to pH = 6 with 1 M HCl and the aqueous phase was extracted with DCM (3 x 10 mL). Then the organic phase was dried with anhydrous $MgSO_4$, filtered and concentrate *in vacuo* to give compound **8** (70 mg, 0.19 mmol, 76%) as a yellow oily solid.

1H NMR (400 MHz, CD_3OD): δ_H 8.41 (d, J = 8.5 Hz, 1 H), 8.08 (d, J = 8.5 Hz, 1 H), 7.76 (br s, 1 H), 7.64 (m, 1 H), 7.53 (t, J = 7.5 Hz, 1 H), 7.32 (d, J = 4.5 Hz, 1 H), 7.07 (t, J = 7.5 Hz, 1 H), 4.46 (br s, 2 H), 3.95 (m, 2 H), 3.75 (tr, J = 6.0 Hz, 2 H), 3.47 (m, 4 H), 2.34 (m, 2 H).

^{13}C NMR (100 MHz, CD_3OD) δ_C 170.9, 155.4, 142.7, 133.7, 131.2, 129.7, 129.3, 128.8, 127.6, 121.2, 119.0, 115.3, 55.2, 54.9, 53.3, 44.5, 40.2, 24.0.

HRMS [+ scan]: calculated m/z $C_{18}H_{22}N_3O_3S$ 360.1382; observed 360.1386.

General procedure for preparation of compound 9

To a mixture of compound **8** (70 mg, 195 μmol, 1.0 *eq*) and propargyl amine (11.2 mg, 200 μmol, 13 μL, 1.0 *eq*) in DMF (3 mL) was added TEA (75.5 mg, 74.6 μmol, 104 μL, 4.0 *eq*) at 25 °C under N_2. 50% T_3P in DMF (238 mg, 370 μmol, 1.9 *eq*) was added to the mixture and stirred at 25 °C for 16 h. The solvent was removed *in vacuo* and the title compound was purified on a column (gradient DCM to DCM:MeOH 9:1). To remove residual DMF, the compound was dissolved in DCM (10 mL) and washed with H_2O (10 mL) and 1% aqueous NaOH solution (10 mL). The organic phase was dried with anhydrous $MgSO_4$, the solvent was removed *in vacuo* to afford **9** (26 mg, 66 μmol, 34% yield, ratio of diastereomers 4:1).

^{1}H NMR (400 MHz, $CDCl_3$, reported for major diastereomer): δ 10.42 (br s, 1 H), 8.35 (d, *J* = 8.9 Hz, 1 H), 7.36 - 7.41 (m, 2 H), 7.26 (m, 1 H), 7.11 (m, 1 H), 7.06 (m, 1 H), 6.87 – 6.97 (m, 2 H), 6.49 (br s, 1 H), 4.16 (dd, *J* = 5.2, 2.5 Hz, 2 H), 3.59 - 3.71 (m, 6 H), 2.75 (br s, 2 H), 2.65 (t, J = 5.5 Hz, 2 H), 2.27 (t, *J* = 2.5 Hz, 1 H), 1.96 (br s, 2 H).

^{13}C NMR (100 MHz, $CDCl_3$) δ_C 169.4, 155.4, 141.5, 140.0, 132.5, 128.4, 126.8, 125.5, 122.7, 121.1, 120.9, 118.9, 79.2, 71.8, 57.4, 55.0, 46.0, 29.6.

HRMS [+ scan]: calculated m/z $C_{36}H_{52}N_9O_7S$ 397.1698; observed 397.1716.

General procedure for preparation of compound TDB-imi6

Compound **9** (9.9 mg, 25 µmol, 1.0 *eq*) was dissolved in a mixture of H_2O (1.7 mL) and tBuOH (0.8 mL). Aqueous $CuSO_4$ solution (250 µL, 100 mM, 25 µmol, 1.0 *eq*) was added, followed by aqueous NaAsc solution (1.3 mL, 100 mM, 130 µmol, 5.2 *eq*). The resulting cloudy yellow mixture was put under argon atmosphere; aqeous solution of Click-Degrader 1 (3.8 mL, 10 mM, 38 µmol, 1.5 *eq*) was then added. The reaction was stirred at room temperature for 1 h, after which they reation mixture turned clear yellow. The reaction was quenched with disodium EDTA dihydrate (9.3 mg, 25 µmol, 1 eq), the organic solvent was removed *in vacuo* and the mixture was purified via HPLC. The fractions containing the product were lyophilised, resulting in **TDB-imi6** as a yellow-brown oily solid (10.2 mg, 14 µmol, 54% yield).

HRMS [+ scan]: calculated m/z $C_{36}H_{52}N_9O_7S$ 754.3710; observed 754.3698.

Scheme 3 | Synthetic route to derivatized pyridostatins.

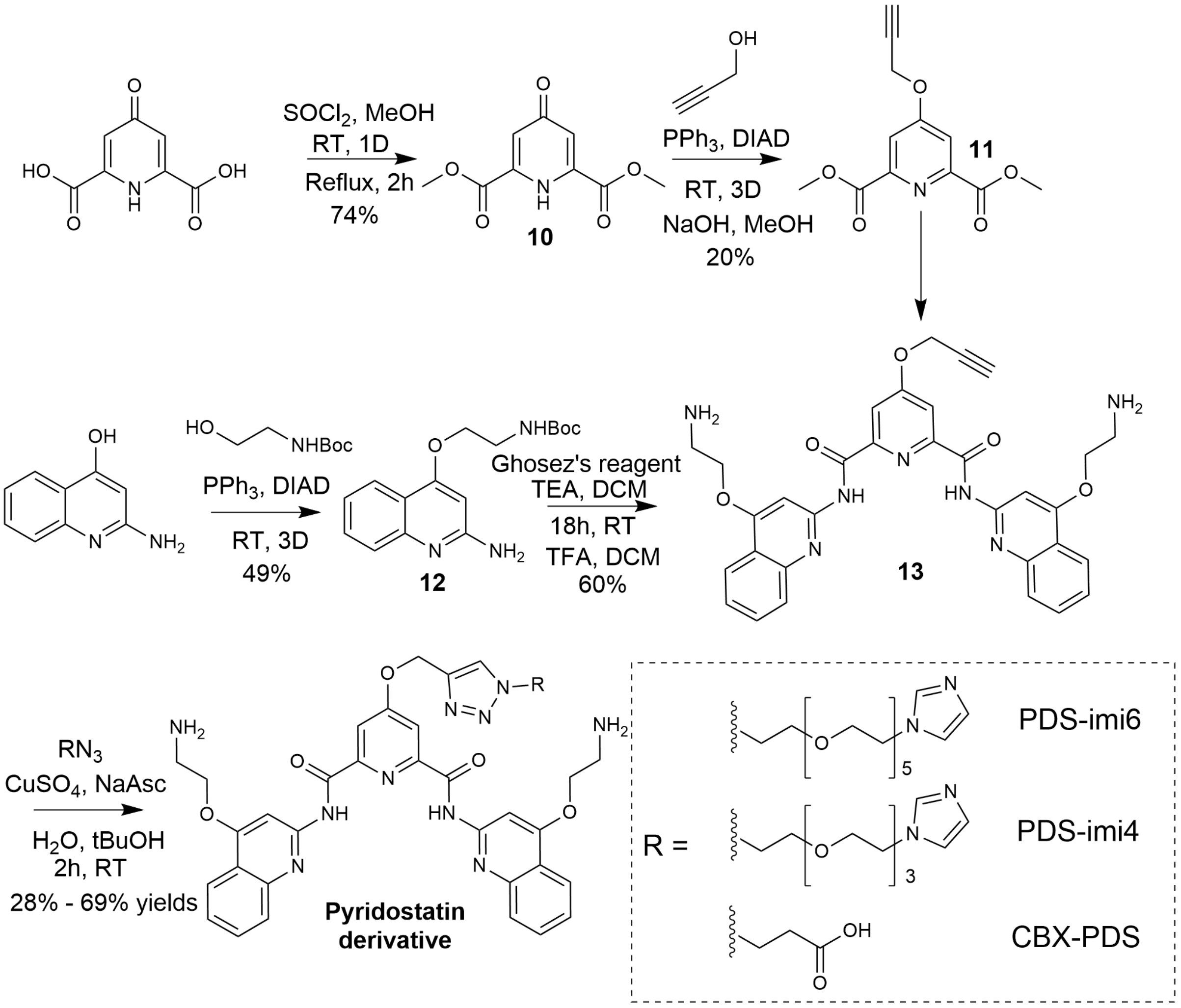

General procedure for preparation of compound 10

The procedures outlined for PDS derivative synthesis were adapted from route reported by di Antonio and colleagues[10]. Chelidamic acid hydrate (2.0 g, 11 mmol) was suspended in 20ml MeOH. $SOCl_2$ (500 µL, 6.9 mmol) was added dropwise to the suspension at -10 ºC. A white to brown colour change was observed. The solution was warmed to 25 ºC and stirred overnight. The brown solution was refluxed for 2 h and the solvent removed *in vacuo*. The brown crude product was then re-crystallised from EtOH, resulting in a beige solid chelidamic acid dimethyl ester **10** (864 mg, 3.9 mmol, 36%).

^{1}H NMR (400 MHz, DMSO) δ 11.77 (br s, 1H), 7.61 (s, 2H), 3.88 (s, 6H). ^{13}C NMR (101 MHz, DMSO) δ 165.97, 164.88, 149.37, 115.33, 52.68.

HRMS [+ scan] calculated for $C_9H_{10}NO_5$ ($[M+H]^+$) m/z: 212.0559, observed 212.0567.

General procedure for preparation of compound 11

Compound **10** (0.82 g, 3.8 mmol), propargyl alcohol (0.33 mL, 5.7 mmol) and polymer-bound triphenylphosphine (3.47 g, 1.5 mmol loading/g, 5.2 mmol) were suspended in 55 mL freshly distilled THF. The solution was degassed using freeze-pump-thaw cycling and cooled to 0 ºC, DIAD (1.0 mL, 5.1 mmol) was added dropwise under argon. The solution was warmed to 25 ºC and stirred for 3 days. The solution was filtered, and the solvent removed *in vacuo*. The dimethyl ester of the title compound was obtained via column chromatography (50% EtOAc, 50% petroleum ether) and immediately hydrolized. The intermediate product was dissolved in 50 mL MeOH and aqueous NaOH (0.33 g, 7.5 mmol in 50 mL of H_2O) solution was added. The solution was stirred for 5 min and hydrolysis was confirmed by TLC. The organic solvent was removed *in vacuo*. 5% formic acid was added to acidify followed by extraction with EtOAC (3×100mL). The organic layer was then dried with $MgSO_4$, filtered and the solvent removed *in vacuo*. This yielded an off-white solid propargylic chelidamic acid **2** (0.17 g, 0.77 mmol, 20%).

^{1}H NMR (500 MHz, CD_3OD) δ 7.93 (s, 2H), 5.02 (d, *J* = 2.4 Hz, 2H), 3.15 (t, *J* = 2.5 Hz, 1H). ^{13}C NMR (126 MHz, CD_3OD) δ 168.12, 166.98, 150.54, 150.43, 115.73, 78.97, 77.90, 77.88, 57.74, 57.68.

HRMS [+ scan] calculated for $C_{10}H_8NO_5$ ($[M+H]^+$) m/z: 222.0397, observed 222.0391.

General procedure for preparation of compound 12

2-aminoquinolinone (1.0 g, 6.2 mmol), N-boc ethanolamine (1.4 mL, 9.1 mmol) and triphenylphosphine (3.3 g, 13 mmol) were dissolved in 10 mL freshly distilled THF. The solution was degassed using freeze-pump-thaw cycling and cooled to 0 ºC, DIAD (1.8 mL, 9.2 mmol) was added dropwise under argon. The solution warmed to 25 ºC and stirred for 3 days. The solvent was then removed *in vacuo*. The product was purified via gradient column chromatography with 100% EtOAc to 90% EtOAc, 10% MeOH. The solvent was removed *in vacuo* to obtain an off-white solid **3** (552 mg, 1.82 mmol, 29%).

^1H NMR (500 MHz, $CDCl_3$) δ 7.98 (dd, *J* = 8.0, 1.0 Hz, 2H), 7.60 (dd, *J* = 8.4, 1.2 Hz, 2H), 7.55 (m, *J* = 8.5, 6.7, 1.6 Hz, 2H), 7.23 (m, *J* = 8.1, 6.6, 1.3 Hz, 2H), 6.04 (s, 1H), 5.01 (br s, 1H), 4.69 (br s, 2H), 4.18 (t, *J* = 5.1 Hz, 4H), 3.68 (q, *J* = 5.5 Hz, 4H), 1.46 (s, 9H).^{13}C NMR (126 MHz, $CDCl_3$) δ 162.32, 158.13, 155.90, 148.55, 130.25, 125.63, 121.97, 121.60, 117.52, 90.09, 79.83, 67.52, 39.82, 28.38.

HRMS [+ scan] calculated for $C_{16}H_{22}N_3O_3$ ($[M+H]^+$) m/z: 304.1661, observed 304.1649.

General procedure for preparation of compound 13 (Alkyne-pyridostatin)

Compound **11** (0.12 g, 0.54 mol) was dissolved in 1.2 mL DCM. Then Ghosez reagent (170 µL, 1.3 mmol) was added dropwise at 0 ºC. The orange solution was then stirred at 25 ºC for 2h. The chlorination was confirmed by TLC. Triethylamine (0.18 mL, 1.3 mmol) was added dropwise at 0 ºC. The solution was then stirred at 25 ºC for 1 h. Compound **12** (0.37 g, 1.2 mmol) was suspended in 1.2 mL DCM and then added dropwise to the mixture. The mixture turned brown-red and was stirred under argon overnight. The crude protected product **13** was precipitated from hot MeCN as a red solid. **13** was then dissolved in DCM. A 2:1 mixture of DCM:TFA was added to acidify the solution and remove the N-Boc protecting group. The solvent was removed *in vacuo* and the product was purified via HPLC (gradient 100% H_2O, 0.1% FA to 100% MeCN, 0.1% FA). Lyophilization afforded an off-white solid Alkyne-pyridostatin **14** (51 mg, 86 µmol, 16%).

HRMS [+ scan] calculated for $C_{32}H_{30}N_7O_5$ ($[M+H]^+$) m/z: 592.2308, observed 592.2327.

General procedure for preparation of derivatized pyridostatin

Alkyne-Pyridostatin **14** (15 mg, 25 µmol) was dissolved in 2.5 mL of a 2:1 mixture of H_2O: tBuOH. Solution of copper sulfate pentahydrate (250 µL, 100 mM, 25 µmol) was added followed by a solution of sodium ascorbate (1.3 mL, 100 mM, 130 µmol). The cloudy yellow solution was degassed and stirred for 10 min. Solution of an organic azide (3.8 mL ,10 mM) was then added. The solution was stirred under argon for 2 h. The organic solvent was removed *in vacuo*. Then the product purified via HPLC

(gradient 100% H_2O, 0.1% FA to 100% MeCN, 0.1% FA). The product was obtained as a white or an off-white solid.

PDS-Imi6. 48% yield (11.3 mg, 12 μmol). HRMS (ES) calculated for $C_{47}H_{52}N_{12}O_{10}$ ([M+H]+) m/z: 949.4321, found 949.4344.

PDS-Imi4. 69% yield (14.8 mg, 17 μmol). HRMS (ES) calculated for $C_{43}H_{49}N_{12}O_{8}$ ([M+H]+) m/z: 861.3796, found 861.376.

CBX-PDS. 28% yield (4.9 mg, 6.9 μmol). HRMS (ES) calculated for $C_{35}H_{34}N_{10}O_{7}$ ([M+H]+) m/z: 707.2690, found: 707.2684.

4. NMR Spectra

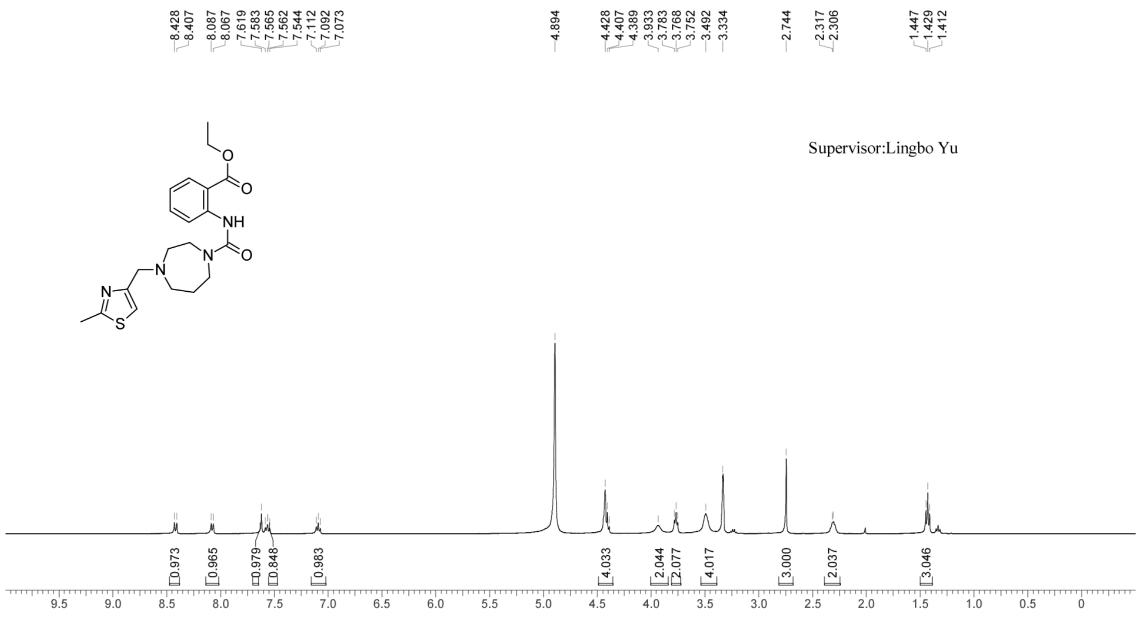

^{1}H NMR (400 MHz, CD_3OD) spectrum of **MTDB**.

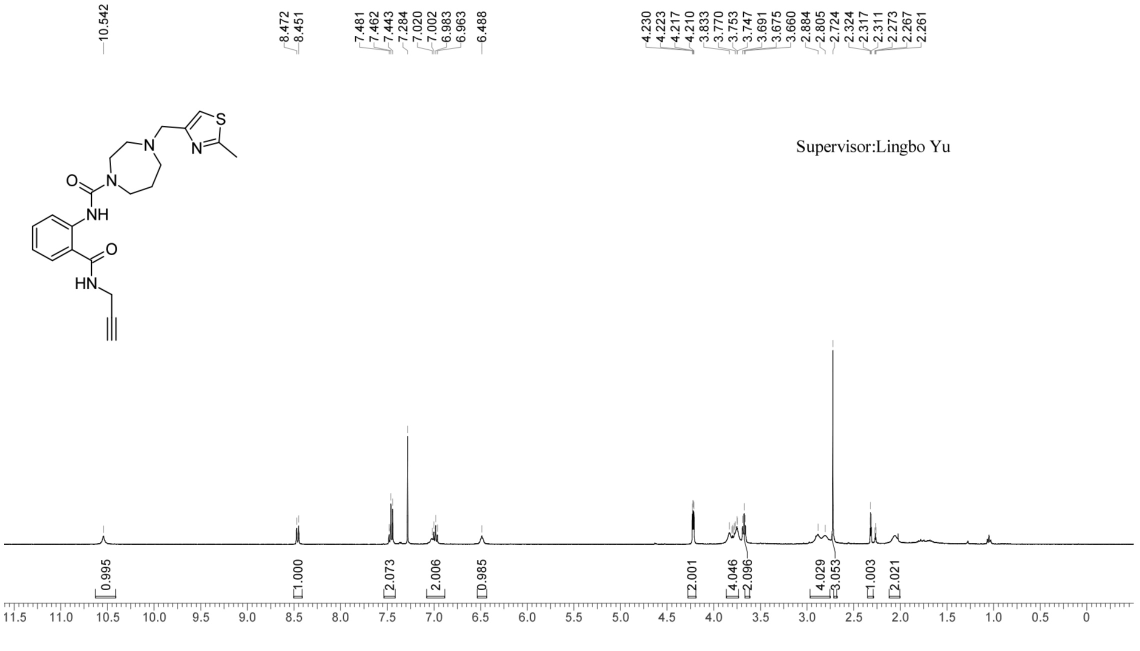

^{1}H NMR (400 MHz, $CDCl_3$) spectrum of **5**.

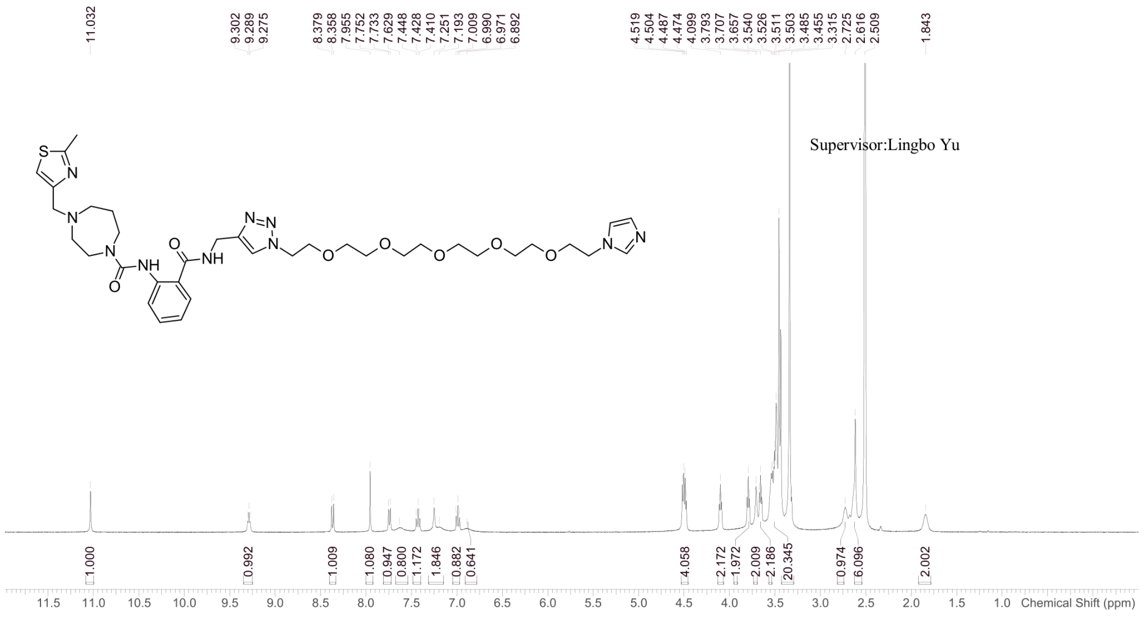

^{1}H NMR (400 MHz, *DMSO*-d$_6$) spectrum of **MTDB-imi6**.

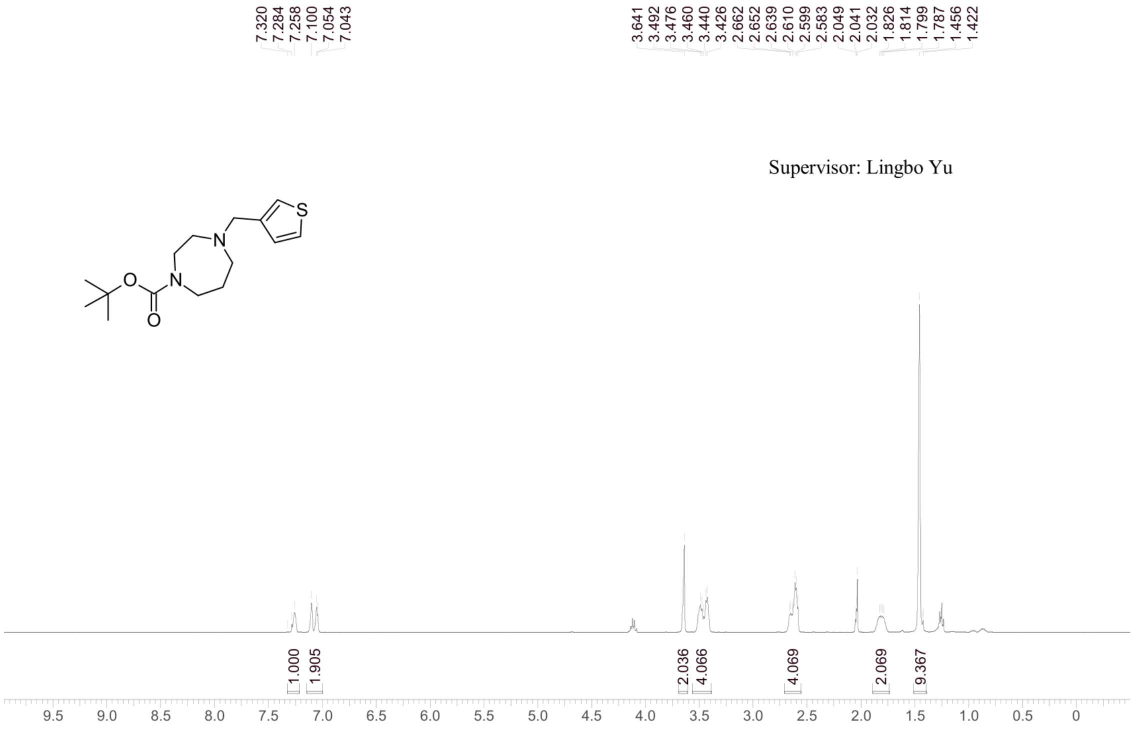

^{1}H NMR (400 MHz, $CDCl_3$) spectrum of **6**.

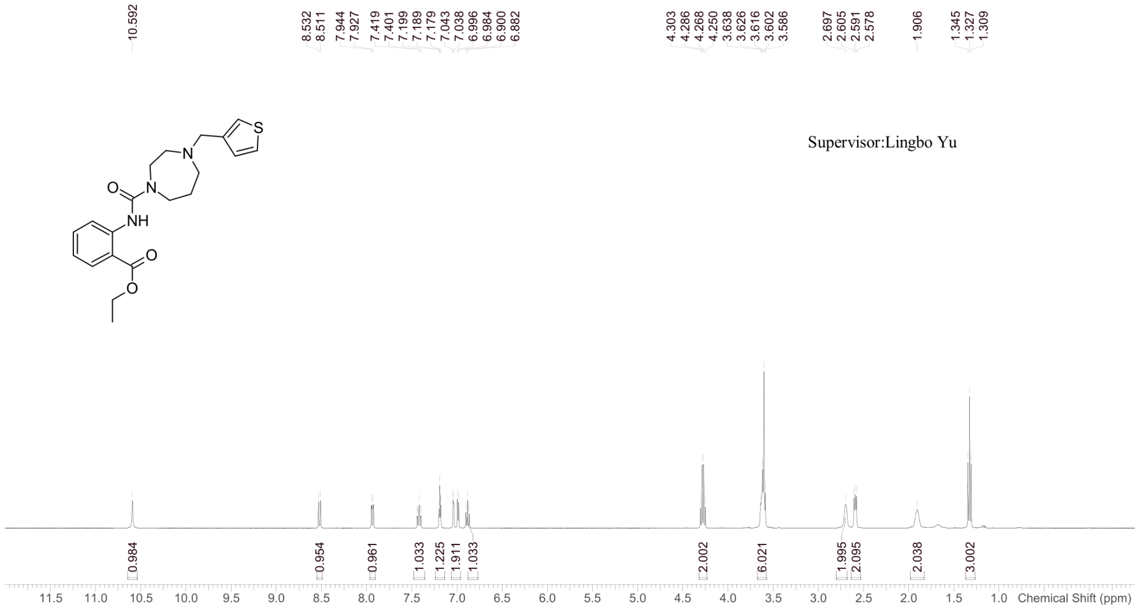

^{1}H NMR (400 MHz, $CDCl_3$) spectrum of **TDB**.

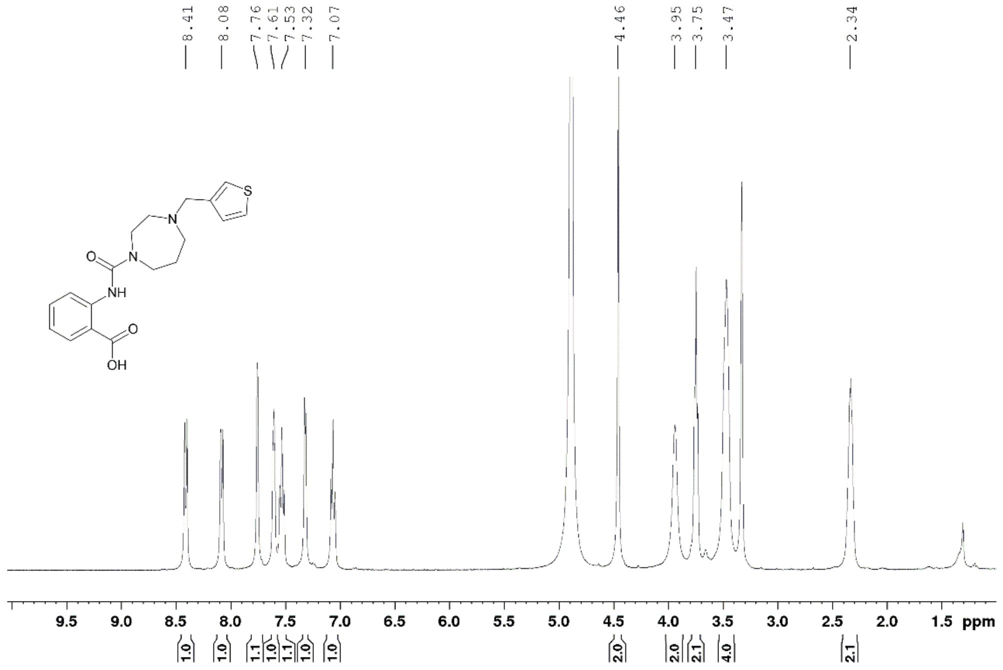

^{1}H NMR (400 MHz, CD_3OD) spectrum of **8**.

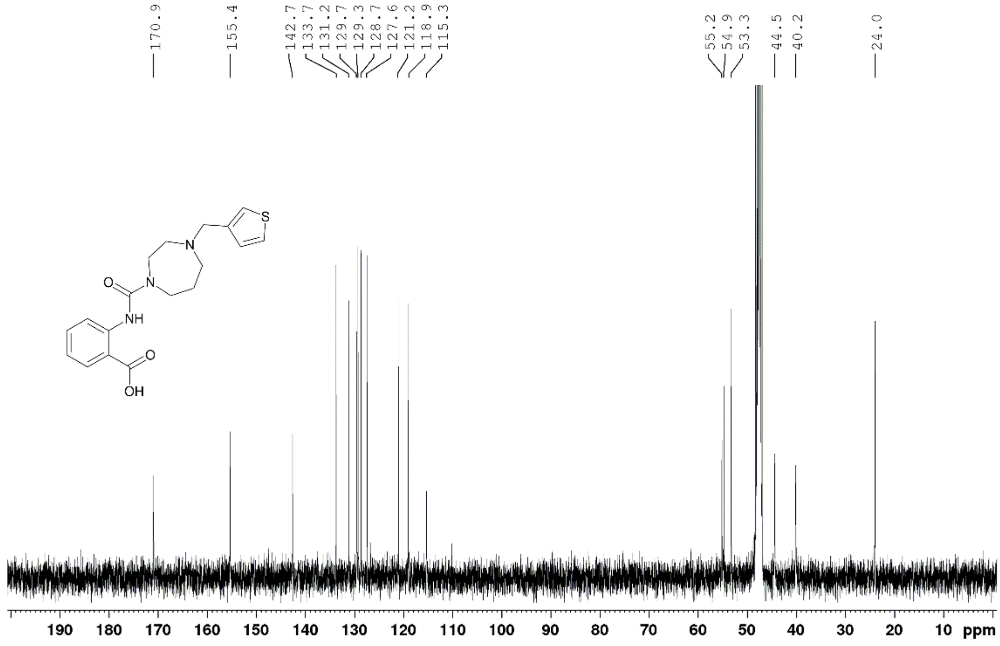

^{13}C NMR (100 MHz, CD_3OD) spectrum of **8**.

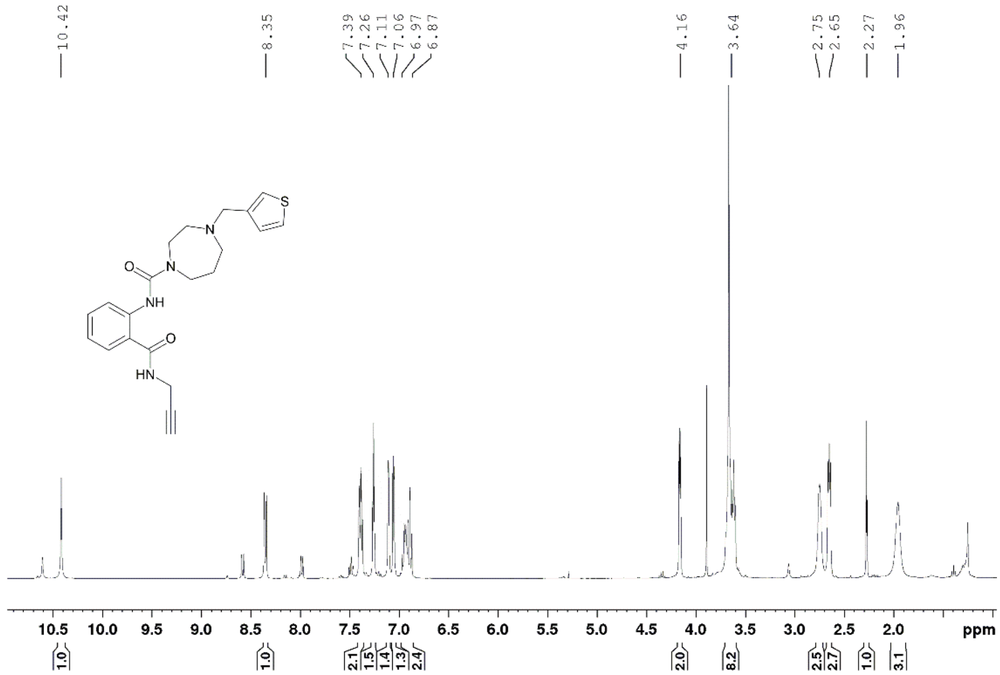

^{1}H NMR (400 MHz, $CDCl_3$) spectrum of **9**.

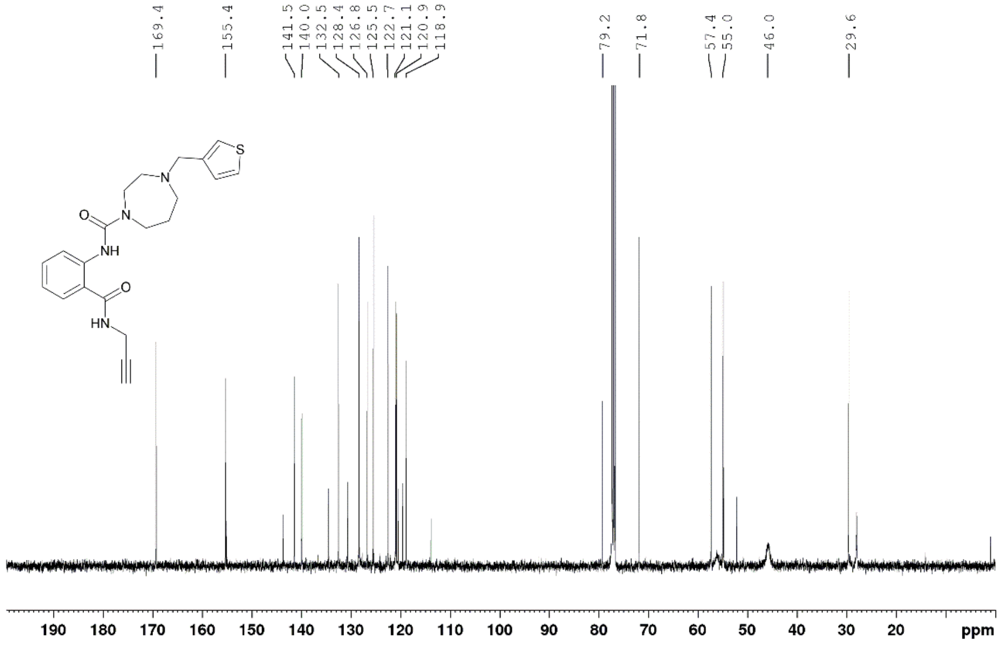

^{13}C NMR (100 MHz, $CDCl_3$) spectrum of **9**.

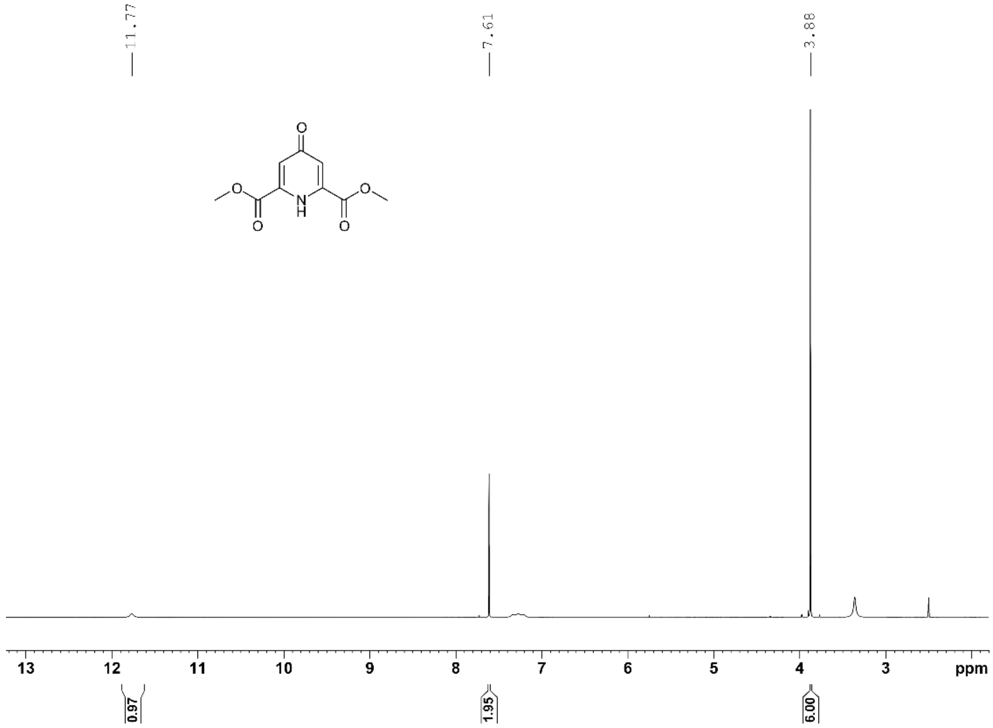

^{1}H NMR (100 MHz, DMSO) spectrum of **10**.

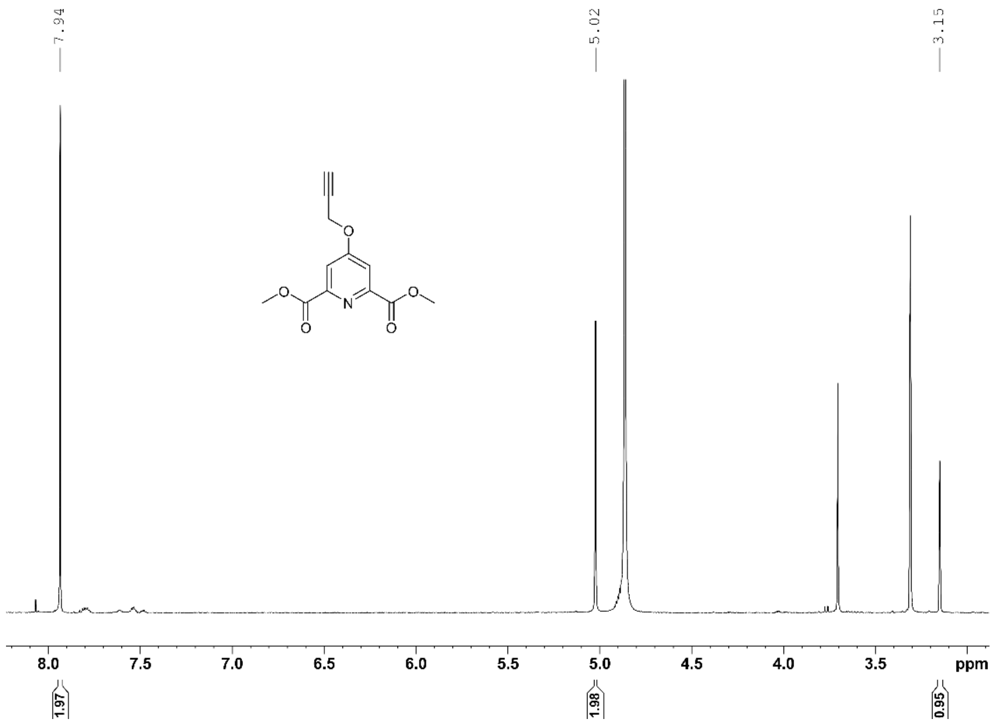

^{1}H NMR (100 MHz, CD_3OD) spectrum of **11**.

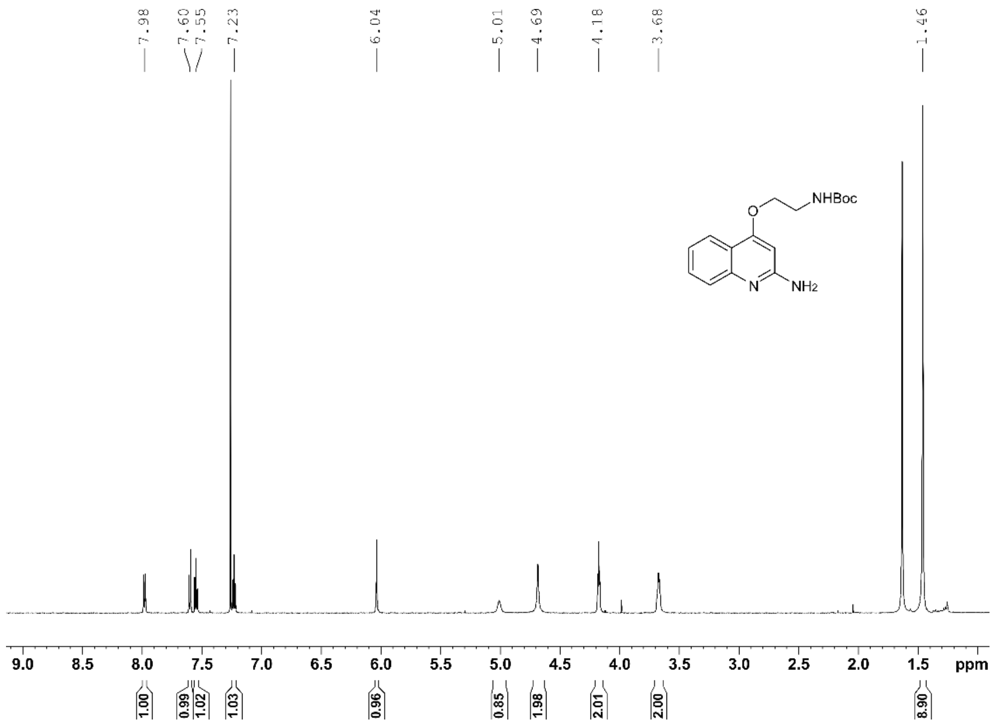

^{1}H NMR (100 MHz, $CDCl_3$) spectrum of **12**.

5. References

(1) Mikutis, S.; Gu, M. X.; Sendinc, E.; Hazemi, M. E.; Kiely-Collins, H.; Aspris, D.; Vassiliou, G. S.; Shi, Y.; Tzelepis, K.; Bernardes, G. J. L. meCLICK-Seq, a Substrate-Hijacking and RNA Degradation Strategy for the Study of RNA Methylation. *Acs Cent. Sci.* **2020**, *6* (12), 2196–2208.

(2) Case, D.A.; Belfon, K.; Ben-Shalom, I.Y.; Brozell, S.R.; Cerutti, D.S.; Cheatham, T.E.,III; Cruzeiro, V.W.D.; Darden, T.A.; Duke, R.E.; Giambasu, G.; Gilson, M.K.; Gohlke, H.; Goetz, A.W.; Harris, R.; Izadi, S.; Iz- mailov, S.A.; Kasavajhala, K.; Kovalenko, A.; Krasny, R.; Kurtzman, T.; Lee, T.S.; LeGrand, S.; Li, P.; Lin, C.; Liu, J.; Luchko, T.; Luo, R.; Man, V.; Merz, K.M.; Miao, Y.; Mikhailovskii, O.; Monard, G.; Nguyen, H.; Onufriev, A.; Pan, F.; Pantano, S.; Qi, R.; Roe, D.R.; Roitberg, A.; Sagui, C.; Schott-Verdugo S.; Shen, J.; Simmerling, C.L.; Skrynnikov, N.R.; Smith, J.; Swails, J.; Walker, R.C.; Wang, J.; Wilson, L.; Wolf, R.M.; Wu, X.; Xiong, Y.; Xue, Y.; York D.M.; Kollman P.A. (2020), AMBER 2020, University of California, San Francisco.

(3) Zgarbova, M.; Otyepka, M.; Sponer, J.; Mladek, A.; Banas, P.; Cheatham, T. E., 3rd; Jurecka, P. Refinement of the Cornell et al. Nucleic Acids Force Field Based on Reference Quantum Chemical Calculations of Glycosidic Torsion Profiles. *J. Chem. Theory. Comput.* **2011**, *7* (9), 2886–2902..

(4) Wang, J.; Wolf, R. M.; Caldwell, J. W.; Kollman, P. A.; Case, D. A. Development and testing of a general amber force field. *J. Comput. Chem.* **2004**, *25* (9), 1157–1174.

(5) Somovilla, V. J.; Bermejo, I. A.; Albuquerque, I. s. S.; Martinez-Saez, N.; Castro-López, J.; García-Martín, F.; Compañón, I.; Hinou, H.; Nishimura, S.-I.; Jiménez-Barbero, J. s. The use of fluoroproline in MUC1 antigen enables efficient detection of antibodies in patients with prostate cancer. *J. Am. Chem. Soc.* **2017**, *139* (50), 18255–18261.

(6) Li, H. Minimap2: pairwise alignment for nucleotide sequences. *Bioinformatics* **2018**, *34* (18), 3094–3100.

(7) Corman, V. M.; Landt, O.; Kaiser, M.; Molenkamp, R.; Meijer, A.; Chu, D. K.; Bleicker, T.; Brunink, S.; Schneider, J.; Schmidt, M. L.; et al. Detection of 2019 novel coronavirus (2019-nCoV) by real-time RT-PCR. *Euro Surveill* **2020**, *25* (3), 2000045.

(8) Lu, X.; Whitaker, B.; Sakthivel, S. K.; Kamili, S.; Rose, L. E.; Lowe, L.; Mohareb, E.; Elassal, E. M.; Al-sanouri, T.; Haddadin, A.; et al. Real-time reverse transcription-PCR assay panel for Middle East respiratory syndrome coronavirus. *J. Clin. Microbiol.* **2014**, *52* (1), 67–75.

(9) Kim, D.; Lee, J. Y.; Yang, J. S.; Kim, J. W.; Kim, V. N.; Chang, H. The Architecture of SARS-CoV-2 Transcriptome. *Cell* **2020**, *181* (4), 914–921 e910.

(10) Di Antonio, M.; Biffi, G.; Mariani, A.; Raiber, E. A.; Rodriguez, R.; Balasubramanian, S. Selective RNA Versus DNA G-Quadruplex Targeting by In Situ Click Chemistry. *Angew. Chem. Int. Ed.* **2012**, *51* (44), 11073–11078.
