# Supplementary material for: Proximity-Induced Nucleic Acid Degrader (PINAD) Approach to Targeted RNA Degradation Using Small Molecules
Source: ACS Cent Sci. 2023 Apr 26;9(5):892–904. doi: 10.1021/acscentsci.3c00015 (PMC10214512; doi:10.1021/acscentsci.3c00015)
Supplement: Supplementary file 2 — oc3c00015_si_002.pdf [file oc3c00015_si_002.pdf]

oc-2023-000153.R1

Name: Peer Review Information for "Proximity-Induced Nucleic Acid Degrader (PINAD) Approach to Targeted RNA Degradation Using Small Molecules"

First Round of Reviewer Comments

Reviewer: 1

Comments to the Author

Mikutis, Rebelo, and Yankova et al present a new method for targeted degradation of RNA transcripts called PINAD. In this approach, a hybrid molecule consisting of an imidazole-based degrader and an RNA-binding small molecule targeting moiety (PDS, MTDB, or TBD) is made. This is used then to show in vitro cutting activity on RNAs containing the RNA motifs that each of the targeting molecules bind to. Another in vitro experiment is performed to show cutting of the SARS-CoV-2 RNA near to the pseudoknot area where MTDB should have specificity. Finally, the authors move to cell and in vivo based assays to examine the effects of PINAD. Across various assays the authors show data that suggest cutting of the target RNAs and subsequent biological consequences therein.

Overall the concept here is interesting and the modularity of the technology seems useful, however the manuscript and data are presented in such a way that it makes it difficult to fully evaluate quality of the data. Specific examples are noted below, but missing figure panels, inconsistent legends, and poorly labeled data all contribute to this issue, throughout the paper.

Major points

Related to Figure 4:

1. Figure 4d is completely missing from this draft of the manuscript and the legend. The later parts of Figure 4's legend seem incongruent with the figure.
2. Earlier on in the figure, the legend also seems a bit inconsistent. It suggests A is only about K+ and B is only about Li+, but there are K+ and Li+ conditions in both A and B
3. Figure 4F is very confusing. The text would lead one to believe that the MTDB-deg cuts the SARS-CoV-2 RNA selectively. The text and legend also suggest that “native SARS-CoV-2 RNA” was extracted and analyzed. However the data in Figure 4F seem to show two major RNA bands which are likely the 18S rRNA and 28S rRNA. Since the gel's ladder is not annotated (another issue needing correcting) it is impossible to tell. Regardless, unless the authors isolated through some affinity purification SARS-CoV-2 viral genomic RNA, any RNA extracted from infected cells would larger be rRNA. Therefore, the data in Figure 4F seem to indicate that in vitro, the MTDB-deg is actually a non-specific RNA cutting conjugate.

4. It would be helpful to better explain the LC-MS based method for reading out the data in Figure 4. After reading the text, it is not clear to me what products were being analyzed and how an assessment of cutting was occurring with LC-MS (or why an RNA gel would not have sufficed).
5. The linker / degrader nomenclature could be tightened up a bit. Some of the names have numbers indicating the length of the linker and the type of active cutter (eg PDS-Imi6) while others have no numbers (eg MTDB-deg) and don't specify "imi" as the cutter. Making all the names consistent will help readers with understanding all the parameters that are changing across the various compounds that have been made in this work.

Related to Figure 5

1. Why are the flanking regions presented completely flat?
2. If you performed nanopore, would you not get the entire viral gRNA? Do you have reads that examine the whole length of the viral RNA in one view? How does this look +/- MTDB-deg?
3. Why do the authors think the two regions shown in Figure 5a and 5b have peaks in the center, while all the data in the Figure S2 are very flat (which would suggest full length reads across this region analyzed)?
4. How did the authors only sequence the SARS-CoV-2 genome? It would seem that the direct RNA kits from ONT would not be selective to the viral genomic RNA. What other RNAs were analyzed? Were there any off-target effects found?
5. If the authors want to claim "The above findings provide strong proof-of-principle that MTDB-deg is a functional and selective degrader of the SARS-CoV-2 pseudoknot and its direct RNA-RNA interactome" an analysis of RNAs in the same pool that are not the viral RNA but also do not get cut + MTDB-deg addition.

Figure 6 also has inconsistent naming schemes - for example the labels in 6C simply say "Deg6" rather than the whole conjugate name. Additionally, the compounds being used in 6A are not clearly labeled on the figure which make it difficult to read.

Figure 7: This figure was labeled too poorly labeled to interpret. Panels in A and B are similarly formatted but have different values. Neither the legend nor the text provides sufficient detail to explain the differences so I can't evaluate this data.

Figure 8c: There are two bands present in the phosho-p38 blot. It is unclear if both or one of them is meaning full. This is important to understand as changes in both are observed in various V or D conditions. Without knowing which bands are specific, it is difficult to interpret this data.

Other conceptual or experimental questions:

1. Is there an expression range within which PINAD works? Are there transcripts that are too highly or lowly expressed to be efficiently target?
2. How important do the authors think the Docking and MD simulations are? Will these need to be performed for every PINAD made? This could reduce the number of labs for which developing these reagents is possible.

3. The increase affinity seen with Deg addition to the MTDB and TBD binders is interesting and noted in the text. Do the authors think this is due to changes in the parent binder molecule or due to the linker + imidazole adding stabilizing effects in the overall binding constant of the PINAD? This is a slightly different question than is being addressed in Figure 3c, as I am curious about the conjugate, not simply the CD1 binding alone.
4. For the viability assays, how long after drug addition are the cells incubated with the compounds?

Reviewer: 2

Comments to the Author

Overall, this is a very nice piece of work. The following are some issues/questions that I suggest the authors consider addressing prior to publishing this paper.

Major Comments

• While the authors include a functionalized PDS ligand control that does not cleave RNA (cPDS) to demonstrate that RNA cleavage does not significantly affect the binding affinities measured, no such control is included for the pseudoknot binding ligands MTDB and TDB. Since the assay used in the affinity measurements of the two binding moieties are different, it should be noted that the increased affinity measured for MTDB-deg compared to MTDB could be due in part to probe cleavage.

• The agarose gel shown in Fig. 4e appears to show MTDB-deg causing total degradation of the SARS-CoV-2 RNA compared to the degrader negative control (MTDB). This result seems surprising given that the MTDB-deg molecule should specifically cut the genomic sequence proximal to the pseudoknot position which would likely result in somewhat more discrete bands (though this may be an issue of detection limits for the products in the gel shown and could be improved by loading more RNA onto the gel). The authors should at least demonstrate the substrate specificity of MTDB-deg by RNA gel by incubating MTDB-deg with an RNA that does not include the target pseudoknot with the expectation that degradation should not be seen.

O A gel should also be done with the RNA oligo substrates used for the other panels in Fig. 4 for further validation of the trends measured by LCMS. Especially since PDS-deg6 and TDB-deg show similar overall degradation values for their oligo substrates by LCMS, but TDB-deg failed to degrade the SARS-CoV-2 RNA when visualized by gel.

• The nanopore sequencing data presented in Fig. 5a and 5b show that the S gene is also affected by MTDB-deg treatment (possibly due to structure bringing it in close proximity to the pseudoknot region), but in Fig. 5c the authors do not probe this region (and others) by RT-qPCR then claim that “MTDB-deg affects the flanks of the pseudoknot area but not any other region of the full length genomic or sub-genomic SARS-CoV-2 RNA”. The authors should either correct this statement, so it is consistent with the Nanopore sequencing results (specifically that the S gene was also affected) or substantiate this claim with further RT-qPCR of the S gene.

• The authors recognize that PDS-deg6 likely affects other RNA molecules with G4 structures in cells but should also attempt to measure degradation for a panel of these G4-containing RNA by RT-qPCR or sequencing. No data apart from LCMS such as RT-qPCR, sequencing, or RNA gels demonstrates that PDS-deg6 effectively cleaves the SARS-CoV-2 genome or any G4-containing substrate which makes the interpretation the results shown in Fig. 6 difficult.

Minor Comments

Suggest a thorough proof-read. Some example issues are:

O Page 2 Line 57: “residues” should be “bases”

O Page 5 Line 33: “ribonuclease A” should be “ribonuclease L”

O Page 7 Line 32: Explanation for how docking aided MTDB-deg chemical design is not clear

O Page 8 Line 45: “residues” should be “nucleotides”

O Page 11 Line 56: “loose” should be “lose”

O Fig. 4 labeling skips 4d, therefore Fig. 4e should be relabeled 4d and 4f should be relabeled 4e

The abstract refers to CRISPR-Cas13 as a “nucleic acid-based” therapeutic agent, while this is partially true due to the gRNA requirement, drawbacks like poor “cellular uptake” and “stability” are not particularly appropriate to the CRISPR-Cas13 platform given the differences in therapeutically relevant delivery used for this technology compared to siRNA and ASOs.

Author's Response to Peer Review Comments:

Please see uploaded letter which contains a detailed point-by-point response to the comments of the reviewers.

Prof. Editor
ACS Central Science

March 9, 2023

We wish to submit the revised manuscript (Manuscript ID: oc-2023-000153) for publication as an Article in *ACS Central Science*. We are grateful to you and the reviewers for insightful comments and for the suggested improvements.

In this revised version, we have addressed the comments of the reviewers and we have added additional data where needed, or where it helped to clarify the points raised. Importantly, we have generated further data to support and illustrate the *in vitro* and *in vivo* specificity of the PINAD molecules.

These were extensive revisions and we are thankful for the time you granted us to perform these experiments and changes, which we believe have improved significantly the quality of our work and further demonstrate the novelty, potential and scope of the proximity-induced RNA degrader approach for targeted RNA degradation.

We hope that you will find our comments and changes useful. Please find in the next page a point-to-point response to each specific question raised (for clarity, comments are in dark and answers for reviewers are shown in blue). We have also highlighted these changes in supporting files, as requested.

Please take note that the figures in this letter are designed to most clearly answer the questions asked by the reviewers and have a different numbering scheme to the manuscript.

Non-scientific changes:

- We have added additional text and data to the SI and corrected the figure and page numbering accordingly.
- We have added the TOC Graphic and Synopsis in the manuscript accordingly.
- We have added one additional co-author, Max Robertson, who synthesised several G4 degraders.

I look forward to your reply.

Yours sincerely,

Gonçalo Bernardes, on behalf of all authors.

Formatting needs:

1. NAME DISCREPANCY: Please ensure that the following names match between P+ and the MS/SI files:
   - J. Pedro Simas (MS/SI) vs Simas, Pedro (P+)
   - Schneekloth JS Jr (MS/SI) vs Schneekloth, John (P+)

   ACTION: The names of the MS and SI are correct. I have now asked the authors to correct their names in P+.

2. TOC MISSING: Provide a TOC image per journal guidelines (3.25 in. × 1.75 in. (8.25 cm × 4.45 cm) ; on the last page of the Manuscript) with the heading "TOC Graphic" above the graphic. Make sure to designate the file as "Graphic for Manuscript."

   ACTION: A TOC graphic is now provided in the last page of the manuscript file.

3. SYNOPSIS MISSING: The synopsis should be no more than 200 characters (including spaces) and should reasonably correlate with the TOC graphic. The synopsis is intended to explain the importance of the article to a broader readership across the sciences. Please place your synopsis in the manuscript file after the TOC graphic.

   ACTION: A synopsis is now provided after the TOC graphic.

4. SI PG#S: The supporting information pages must be numbered consecutively, starting with page S1.

   ACTION: The SI is now numbered starting from S1.

Reviewer#1:

Recommendation: Reconsider after major revisions noted.
Quality of experimental data, technical rigor: Moderate
Significance to chemistry researchers in this and related fields: High
Broad interest to other researchers: Top 5%
Novelty: Top 5%
Is this research study suitable for media coverage or a First Reactions (a News & Views piece in the journal)?: No

Mikutis, Rebelo, and Yankova et al present a new method for targeted degradation of RNA transcripts called PINAD. In this approach, a hybrid molecule consisting of an imidazole-based degrader and an RNA-binding small molecule targeting moiety (PDS, MTDB, or TBD) is made. This is used then to show in vitro cutting activity on RNAs containing the RNA motifs that each of the targeting molecules bind to. Another in vitro experiment is performed to show cutting of the SARS-CoV-2 RNA near to the pseudoknot area where MTDB should have specificity. Finally, the authors move to cell and in vivo based assays to examine the effects of PINAD. Across various assays the authors show data that suggest cutting of the target RNAs and subsequent biological consequences therein.

ACTION: We thank the reviewer for the excellent suggestions, which we believe have strengthen the quality of the work.

Overall the concept here is interesting and the modularity of the technology seems useful, however the manuscript and data are presented in such a way that it makes it difficult to fully evaluate quality of the data. Specific examples are noted below, but missing figure panels, inconsistent legends, and poorly labeled data all contribute to this issue, throughout the paper.

ACTION: We agree with the reviewer that this approach is modular and thus may find key application in targeted RNA degradation.

Major points Related to Figure 4:

1. Figure 4d is completely missing from this draft of the manuscript and the legend. The later parts of Figure 4's legend seem incongruent with the figure.

ACTION: We apologize for this formatting error. Labels have been made consistent.

2. Earlier on in the figure, the legend also seems a bit inconsistent. It suggests A is only about K+ and B is only about Li+, but there are K+ and Li+ conditions in both A and B

ACTION: We apologize for improperly labelling this figure. Both the main text and the figure have now better reflect the fact that the two panels describe G4-competent and non-G4-competent oligonucleotides.

3. Figure 4F is very confusing. The text would lead one to believe that the MTDB-deg cuts the SARS-CoV-2 RNA selectively. The text and legend also suggest that "native SARS-CoV-2 RNA" was extracted and analyzed. However the data in Figure 4F seem to show two major RNA bands which are likely the 18S rRNA and 28S rRNA. Since the gel's ladder is not annotated (another issue needing correcting) it is impossible to tell. Regardless, unless the authors isolated through some affinity purification SARS-CoV-2 viral genomic RNA, any RNA extracted from infected cells would larger be rRNA. Therefore, the data in Figure 4F seem to indicate that in vitro, the MTDB-deg is actually a non-specific RNA cutting conjugate.

ACTION: We apologise for the lack of clarity regarding the origin of viral genomic RNA used to perform this experiment. Please note that we harvested the supernatant from *in vitro* cultures of cells infected with SARS-CoV-2 (virion RNA), but not infected cells. In the supernatant we expect to have mostly viral particles and cell contamination is minimal. Furthermore, we ultra-centrifuged the supernatant at 6000 g for 10 hours to concentrate the viral particles, before performing any RNA extraction. For clarity, we have now included this information in the methods section and the manuscript text. Moreover, the Nanopore results illustrated in Figure 5 and Figure S3 were originated by using viral RNA extracted following the above strategy and the sequencing QC showed 83% of the reads mapping to SARS-COV-2 genome (**Figure 5b**, shown below) which is considered computationally quite optimal.

Furthermore, as per reviewer's request, we are now providing ladder annotation for the gel shown in

Figure 4e. To further support and illustrate the *in vitro* and *in vivo* specificity of MTDB-deg we now provide: a) additional RNA gel experiment using total RNA extracted from a non-infected mammalian cell line (HEK293FT) were we confirm lack of cutting/degradation of non-viral RNA (**Figure 4h**, shown below), and b) RT-qPCR validation of *18S* transcript levels after dosing VERO-CCL-81 cells infected with SARS-CoV-2 and treated with 3 different doses of MTDB-imi6 (6.2, 12.5 and 25 μM), showing no differential effect on *18S* transcript levels (**Figure 5e**, shown below). In addition, the pseudoknot-specific effects shown in **Figure 5** and **Figure S3** solidify the specificity of MTDB-deg as an RNA cutting bifunctional molecule.

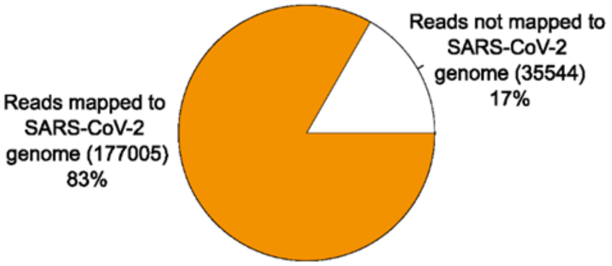

Figure 5b | Pie chart showing that the vast majority of Nanopore reads were mapped to the SARS-CoV-2 genome.

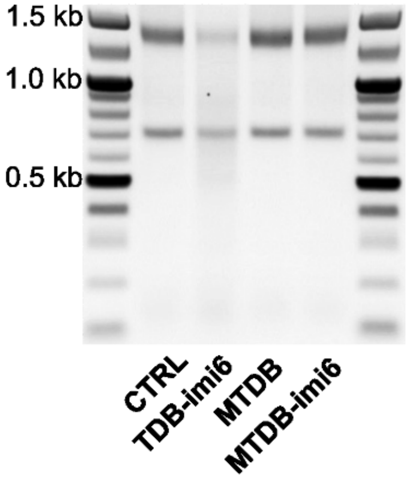

Figure 4h | Agarose gel shows no RNA degradation for MTDB-imi6 and some non-specific degradation for TDB-imi6, on RNA extracted from HEK293FT cells.

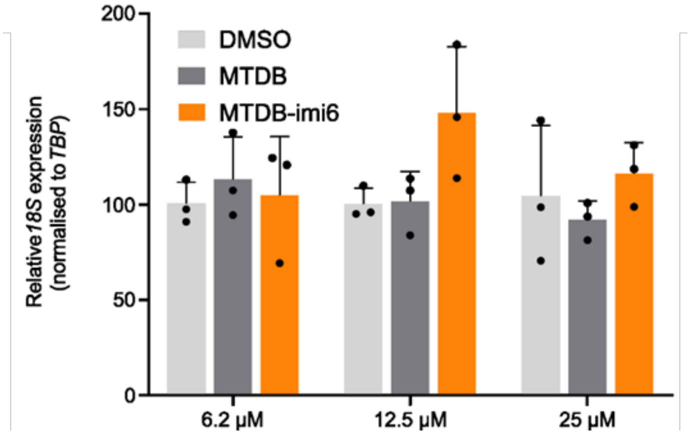

Figure 5e | qPCR validation shows that MTDB and MTDB-imi6 do not degrade or otherwise affect the abundance of 18S rRNA in the concentration range tested.

4. It would be helpful to better explain the LC-MS based method for reading out the data in Figure 4. After reading the text, it is not clear to me what products were being analyzed and how an assessment of cutting was occurring with LC-MS (or why an RNA gel would not have sufficed).

ACTION: We apologize for the lack of clarity regarding the LC-MS experiments. We have now added a brief description in the main text, a more detailed description of the method in the supplementary (section **LC-MS analysis of oligonucleotides**) and a new **Figure S2** to support it, attached below. Briefly, we first find several m/z values that correspond to the oligonucleotide (e.g., the pseudoknot) being analyzed and have a linear relationship between the signal and the injected concentration; then we analyze oligonucleotides that have been treated with one of the PINADs and use combination of the abovementioned m/z values to determine their remaining, non-degraded concentration. We also analyze the UV-Vis trace of the oligonucleotides which, in the example below, provide further evidence for degradation but due to noisiness of the data cannot be used quantitatively. As additional validation, we have also run fluorophore-tagged RNA gels which confirm that the MTDB-PINADs affect the pseudoknot but otherwise provides limited information about the degradation.

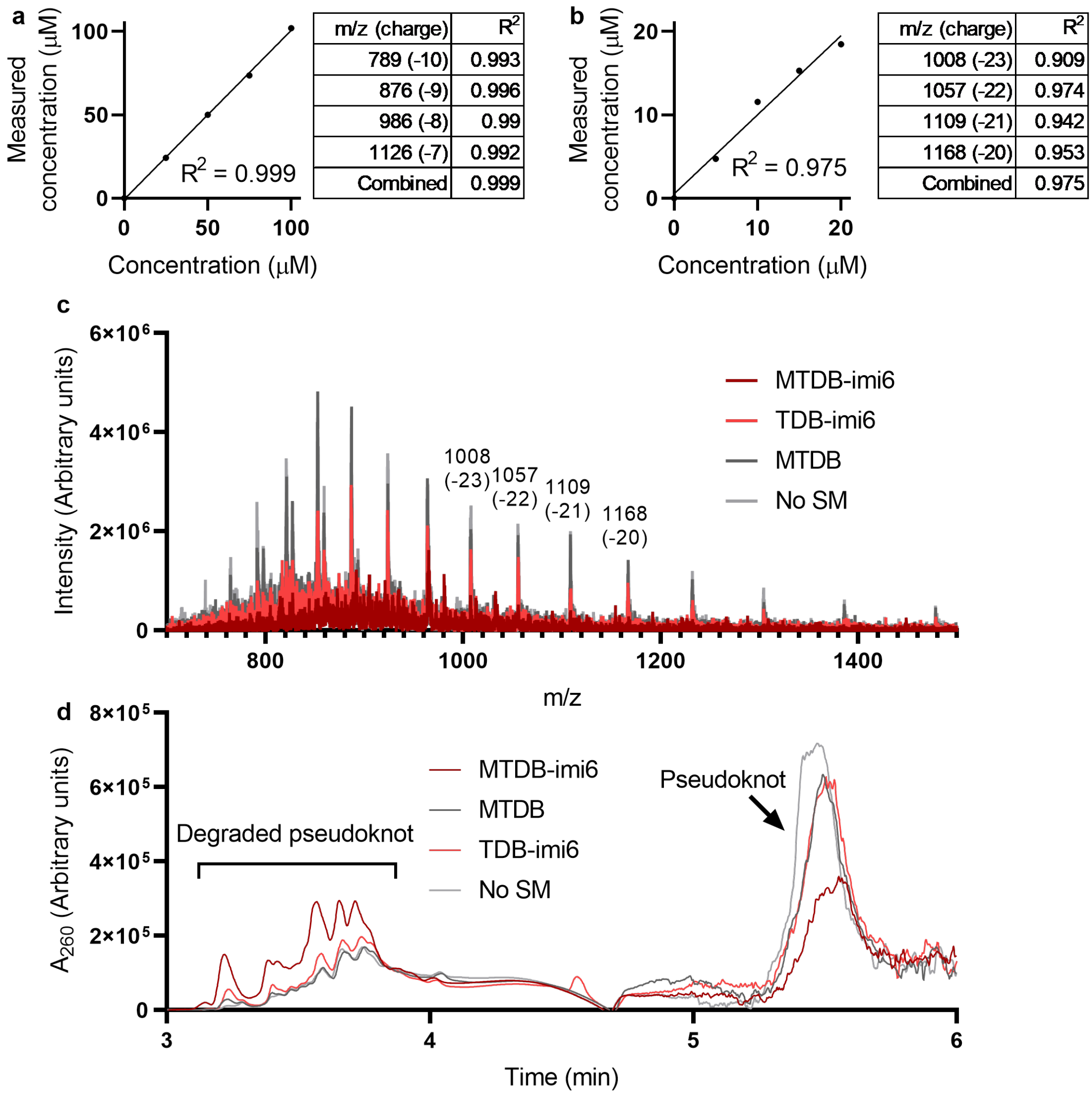

Fig. S2 | Degradation of RNA oligonucleotides can be analyzed using liquid chromatography-mass spectrometry (LCMS). (a) Calibration curve based on four combined m/z values and R^2 values for individual and combined masses for the rG4-forming NRAS oligonucleotide. (b) Calibration curve based on four combined m/z values and R^2 values for individual and combined masses for the pseudoknot oligonucleotide. (c) An example of ion series corresponding to the pseudoknot after a three-hour incubation with MTDB-imi6 and control molecules. m/z values used to calculate the concentration of the nucleotide are shows. Degradation of the pseudoknot leads to depletion of m/z signals. (d) An example of noise-

subtracted A_{260} chromatograms corresponding to the pseudoknot after a three-hour incubation with MTDB-deg6 and control molecules.

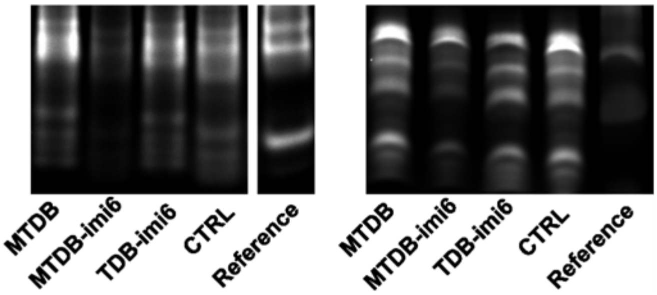

Figure 4d,e | PAGE gels show that MTDB-PINADs affect pseudoknot oligonucleotide. Non-denaturing and denaturing gel validation of pseudoknot degraders on a 5'FAM-tagged pseudoknot oligonucleotide. Double band for the pseudoknot might correspond to a pseudoknot monomer and dimer [see, *Nucleic Acids Res* **2013**, *41* (4), 2594–2608].

5. The linker / degrader nomenclature could be tightened up a bit. Some of the names have numbers indicating the length of the linker and the type of active cutter (eg PDS-Imi6) while others have no numbers (eg MTDB-deg) and don't specify "imi" as the cutter. Making all the names consistent will help readers with understanding all the parameters that are changing across the various compounds that have been made in this work.

ACTION: We thank the reviewers for this suggestion to make notation more consistent. We have now adapted PDS-imi6 type of notation for all the PINADs described in the text.

Related to Figure 5

1. Why are the flanking regions presented completely flat?

ACTION: In **Figure 5c** and **Figure S2** we are exclusively presenting the reads that mapped on the relevant regions, (either belonging to the full-length/unspliced or the subgenomic RNA, as described in the methods section) in order to highlight the presence or absence of on-target effects by MTDB-deg. To avoid confusion, we have now removed the curves with 0 values outside those regions (new **Figure 5c** and new **Figure S2**).

2. If you performed nanopore, would you not get the entire viral gRNA? Do you have reads that examine the whole length of the viral RNA in one view? How does this look +/- MTDB-deg?

ACTION: There are no reads in our dataset covering the entire SARS-COV-2 RNA which is anticipated as its full size is around 30kb. Considering the manufacturers guidelines, the current Nanopore direct RNA sequencing can cover RNA lengths of up to 18-21kb at the very best. However, our analysis is fully in line with previously published SARS-COV-2 studies using the same Nanopore platform (direct RNA seq). We have now added the relevant citations in the manuscript text.

3. Why do the authors think the two regions shown in Figure 5a and 5b have peaks in the center, while all the data in the Figure S2 are very flat (which would suggest full length reads across this region analyzed)?

ACTION: We thank the reviewer for this comment. As we described above, we harvested the supernatant from *in vitro* cultures of cells infected with SARS-CoV-2 (i.e. virion RNA). Therefore, the main reason for the presence of the peaks in **Figure 5a** and **5c** is the relative paucity of SARS-CoV-2 virion RNA on the Nanopore RNA platform possibly due to complex structural properties of those regions, as previously shown by Taiaroa *et al.* (see below relevant figure from Taiaroa *et al.*, bioRxiv 2020.03.05.976167; doi: https://doi.org/10.1101/2020.03.05.976167). As expected, the same was not observed when analysing the subgenomic RNAs, mainly due to the lack of paucity, higher coverage and the fact that Nanopore direct RNA sequencing is a 3'-biased technology.

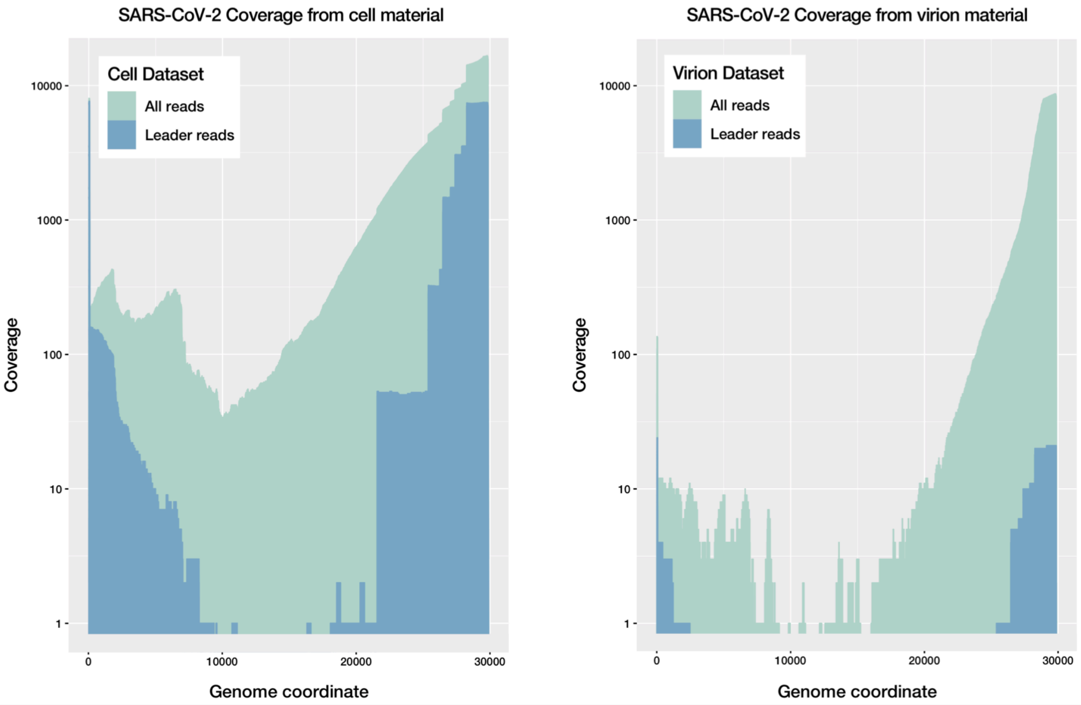

Rebuttal Figure 1 | Native RNA Nanopore sequence of the SARS-CoV-2 genome coverage for cell-culture and virion-derived material. a, Coverage of the SARS-CoV-2 genome for the cell-culture dataset, for all reads and for those predicted to be intact mRNA transcripts or 'leader reads', showing an abundance of such transcripts. **b**, Coverage of the SARS-CoV-2 genome for the virion-derived dataset, showing a relative paucity of intact mRNA transcripts. This is a supplementary figure illustrated in Taiaroa *et al, bioRxiv* 2020.03.05.976167; doi: https://doi.org/10.1101/2020.03.05.976167.

4. How did the authors only sequence the SARS-CoV-2 genome? It would seem that the direct RNA kits from ONT would not be selective to the viral genomic RNA. What other RNAs were analyzed? Were there any off-target effects found?

ACTION: We again apologise for the lack of clarity regarding the origin of viral genomic RNA used to perform this experiment, which we clarified already in point 3 related to **Figure 4**. In short, we harvested the supernatant from *in vitro* cultures of cells infected with SARS-CoV-2, but not infected cells. In the supernatant we expect to have mostly viral particles and cell contamination is minimal. Furthermore, we ultracentrifuged the supernatant at 6000 g for 10 hours to concentrate the viral particles, before performing any RNA extraction. We have now included this information in the methods and manuscript text.

By utilising Nanopore Direct RNA sequencing using RNA extracted as above, we had the ability map and quantify the full-length as well as the subgenomic RNA regions of SARS-COV-2 in isolation. We observed very good mapping quality with 83% of the reads mapping to SARS-COV-2 genome (new **Figure 5b**). That allowed us to show that MTDB-deg is cutting the unspliced/full-length and its RNA-RNA interactions (S region) but not any of the subgenomic RNAs as illustrated in **Figure 5a,c** and **Figure S2**. No off-target effects were found after either *in vitro* or *in vivo* treatment with MTDB-deg which is now well-supported by our existing and newly-provided results.

5. If the authors want to claim "The above findings provide strong proof-of-principle that MTDB-deg is a functional and selective degrader of the SARS-CoV-2 pseudoknot and its direct RNA-RNA interactome" an analysis of RNAs in the same pool that are not the viral RNA but also do not get cut + MTDB-deg addition.

In order to address this comment, we kindly ask the reviewer to consider our answers and new results provided to respond to the above/previous comment as well as the one we provide further above in response to their 3rd point related to **Figure 4**.

Figure 6 also has inconsistent naming schemes - for example the labels in 6C simply say "Deg6" rather

than the whole conjugate name. Additionally, the compounds being used in 6A are not clearly labeled on the figure which make it difficult to read.

ACTION: We apologize for this inconsistency. As per reviewer's suggestion, the figure will now say PDS-imi6.

Figure 7: This figure was labeled too poorly labeled to interpret. Panels in A and B are similarly formatted but have different values. Neither the legend nor the text provides sufficient detail to explain the differences so I can't evaluate this data.

ACTION: We apologize to the reviewer for poor labelling of panels A and B. Here, panel A corresponds to cells being treated an hour before the infection; B correspond to treatment one hour after the infection. This has now been clarified in the figure and emphasized in the figure caption.

Figure 8c: There are two bands present in the phosho-p38 blot. It is unclear if both or one of them is meaning full. This is important to understand as changes in both are observed in various V or D conditions. Without knowing which bands are specific, it is difficult to interpret this data.

Action: We thank the reviewer for this comment. According to the manufacturer of the antibody, the bottom band (size of 40 kDa) belongs to Phosho-p38 while the upper band belongs to non-specific signalling. We have now added additional labelling on the western-blots in **Figure 8c** so it is clearer for the readers. Therefore, we show that reduction of Phosho-p38 is exclusively decreasing upon *in vivo* treatment with MTDB-deg

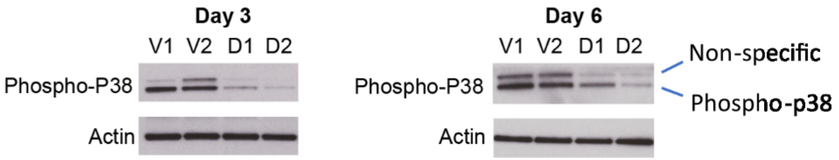

Figure 8c | Western blot analysis of phospho-p38 from lung extracts of transgenic K18-hACE2 mice treated with three doses of 10 mg/kg of vehicle (V1, V2) or MTDB-imi6 (D1, D2) at 1 h before infection and 1 and 2 days post-infection, with labelled phospho-p38 (n=2).

Other conceptual or experimental questions:
1. Is there an expression range within which PINAD works? Are there transcripts that are too highly or lowly expressed to be efficiently target?

ACTION: Most of this work demonstrates that PINADs function against high-abundance transcripts such as viral RNA, further work is needed to demonstrate their utility for targeted degradation of low-abundance transcripts. We have added these considerations to the conclusions section.

2. How important do the authors think the Docking and MD simulations are? Will these need to be performed for every PINAD made? This could reduce the number of labs for which developing these reagents is possible.

ACTION: Although performing MD calculations is not a requirement, nor is it necessary for every PINAD, they can help in the development of improved PINADs. For example, simulations can provide insight into the ideal size of the linker or the regions that the imidazole can reach to be a more efficient degrader. In the present study, we used both docking and MD simulations to gain a deeper understanding of the interactions between our designed PINADs and the pseudoknot at the atomic level. Our results show that the triazole in **MTDB-deg** and the oxygen atoms of the linker form transient hydrogen bonds that increase the binding energy compared to the parent compound, as observed experimentally. The flexible linker in **MTDB-deg** also allows the imidazole to interact with different regions of the RNA, resulting in more efficient degradation of the nucleic acid. These simulations also explain why replacing the ester in **MTDB** with an amide group has no negative effect on binding, as the CONH forms hydrogen bonds with the RNA.

3. The increase affinity seen with Deg addition to the MTDB and TBD binders is interesting and noted in the text. Do the authors think this is due to changes in the parent binder molecule or due to the linker + imidazole adding stabilizing effects in the overall binding constant of the PINAD? This is a slightly different question than is being addressed in Figure 3c, as I am curious about the conjugate, not simply the CD1 binding alone.

ACTION: We thank the referee for pointing out this interesting point. As we have indicated in previous response, our MD simulations indicate that, in the case of **MTDB-deg**, the triazole and the oxygen atoms of the linker form transient hydrogen bonds with the RNA molecule, leading to a potential increase in binding energy compared to the parent compound. This has been clearly explained in the updated manuscript.

4. For the viability assays, how long after drug addition are the cells incubated with the compounds?

ACTION: In the viability assays cells were incubated with the compounds for 24 hours. We thank the reviewer for noting that this information was missing in the manuscript, and we have now included it in the results section and methods.

Reviewer#2:
Recommendation: Reconsider after major revisions noted.
Quality of experimental data, technical rigor: High
Significance to chemistry researchers in this and related fields: Top 5%
Broad interest to other researchers: Top 5%
Novelty: Top 5%
Is this research study suitable for media coverage or a First Reactions (a News & Views piece in the journal)?: Yes

Overall, this is a very nice piece of work.

ACTION: We thank the reviewer for the excellent comments and suggestions about our work.

The following are some issues/questions that I suggest the authors consider addressing prior to publishing this paper.

Major Comments

- While the authors include a functionalized PDS ligand control that does not cleave RNA (cPDS) to demonstrate that RNA cleavage does not significantly affect the binding affinities measured, no such control is included for the pseudoknot binding ligands MTDB and TDB. Since the assay used in the affinity measurements of the two binding moieties are different, it should be noted that the increased affinity measured for MTDB-deg compared to MTDB could be due in part to probe cleavage.

ACTION: We thank the reviewer for this observation. A comment about this possibility has been included in the text; we would also like to emphasize that our computational modelling shows the linker can also form additional interactions which might have some influence on the K_D, as was observed.

- The agarose gel shown in Fig. 4e appears to show MTDB-deg causing total degradation of the SARS-CoV-2 RNA compared to the degrader negative control (MTDB). This result seems surprising given that the MTDB-deg molecule should specifically cut the genomic sequence proximal to the pseudoknot position which would likely result in somewhat more discrete bands (though this may be an issue of detection limits for the products in the gel shown and could be improved by loading more RNA onto the gel). The authors should at least demonstrate the substrate specificity of MTDB-deg by RNA gel by incubating MTDB-deg with an RNA that does not include the target pseudoknot with the expectation that degradation should not be seen.

ACTION: We thank the reviewer for this constructive comment. We are now providing additional supporting results which highlight the *in vitro* and *in vivo* specificity of MTDB-deg: a) RNA gel experiment using total RNA extracted from a non-infected mammalian cell line (HEK293FT) were we confirm lack of cutting/degradation of non-viral RNA, predominantly rRNA (**Fig. 4h**, shown above), and b) RT-qPCR validation of *18S* transcript levels after dosing VERO-CCL-81 cells infected with SARS-CoV-2 and treated with 3 different doses of MTDB-deg (6.2, 12.5 and 25 μM), showing no differential effect on *18S* transcript levels (Fig. 5e, shown above). In addition, the pseudoknot-specific effects shown in **Figure 5** and **Figure S3** solidify the substrate specificity of MTDB-deg in an unbiased fashion.

o A gel should also be done with the RNA oligo substrates used for the other panels in Fig. 4 for further validation of the trends measured by LCMS. Especially since PDS-deg6 and TDB-deg show similar overall degradation values for their oligo substrates by LCMS, but TDB-deg failed to degrade the SARS-CoV-2 RNA when visualized by gel.

ACTION: We thank the reviewer for the suggestion to analyze RNA degradation via gels. For this aim, we utilized 5'FAM-labelled pseudoknot oligonucleotides. We observed consistent pseudoknot depletion with MTDB-imi6 but less consistent effect with TDB-imi6 (Fig. 4d, e, shown below). As further evidence for the degradation, we now show chromatograms corresponding to the pseudoknot LC-MS experiments (**Fig. S2d**, shown above). They have a clear degradation pattern, especially with MTDB-imi6, as the LC-MS assay is fluorophore-free thus a more direct measure of degradation.

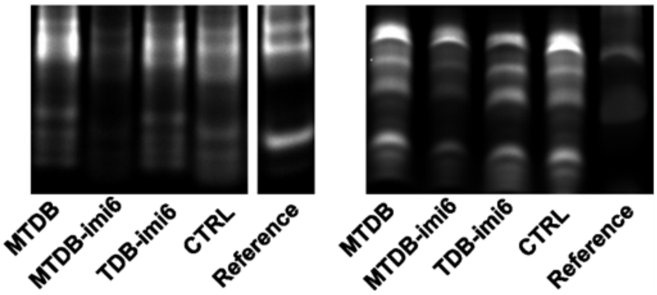

Fig. 4d,e | PAGE gels show that MTDB-PINADs affect pseudoknot oligonucleotide. Non-denaturing and denaturing gel validation of pseudoknot degraders on a 5'FAM-tagged pseudoknot oligonucleotide. Double band for the pseudoknot might correspond to a pseudoknot monomer and dimer [see, *Nucleic Acids Res* **2013**, *41* (4), 2594–2608].

- The nanopore sequencing data presented in Fig. 5a and 5b show that the S gene is also affected by MTDB-deg treatment (possibly due to structure bringing it in close proximity to the pseudoknot region), but in Fig. 5c the authors do not probe this region (and others) by RT-qPCR then claim that "MTDB-deg affects the flanks of the pseudoknot area but not any other region of the full length genomic or sub-genomic SARS-CoV-2 RNA". The authors should either correct this statement, so it is consistent with the Nanopore sequencing results (specifically that the S gene was also affected) or substantiate this claim with further RT-qPCR of the S gene.

ACTION: We agree with the reviewer. We have amended that sentence in the text in order to be less confusing and in line with our unbiased findings in **Figure 5** on the observed effects of MTDB-deg on pseudoknot and its RNA-RNA interactions.

- The authors recognize that PDS-deg6 likely affects other RNA molecules with G4 structures in cells but should also attempt to measure degradation for a panel of these G4-containing RNA by RT-qPCR or sequencing. No data apart from LCMS such as RT-qPCR, sequencing, or RNA gels demonstrates that PDS-deg6 effectively cleaves the SARS-CoV-2 genome or any G4-containing substrate which makes the interpretation the results shown in Fig. 6 difficult.

ACTION: As a response to this comment, we now provide new RT-qPCR results (**Fig. 6e**, shown below) illustrating effects on the known G4-containing RNA *SYNCRIP* in MOLM-13 cells, showing significant effect on this RNA (loss) only when PDS-deg4 or PDS-deg6 was used as a treatment in culture. Interestingly, an opposite effect was observed with the G4 binder, possible a result of stabilisation of the G4 structure.

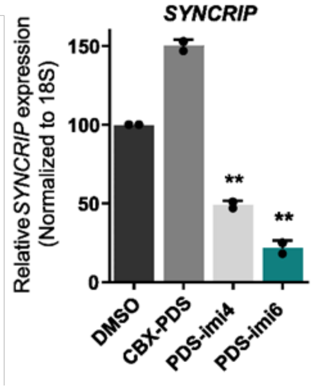

Fig. 6e | RT-qPCR validation of degradation specificity in a cellular system using MOLM-13 cells treated with the indicated doses of CBX-PDS, PDS-deg4, PDS-deg6 or vehicle for 24 h (n=2).

Minor Comments
Suggest a thorough proof-read. Some example issues are:

- Page 2 Line 57: “residues” should be “bases”
- Page 5 Line 33: “ribonuclease A” should be “ribonuclease L”
- Page 7 Line 32: Explanation for how docking aided MTDB-deg chemical design is not clear
- Page 8 Line 45: “residues” should be “nucleotides”
- Page 11 Line 56: “loose” should be “lose”
- Fig. 4 labeling skips 4d, therefore Fig. 4e should be relabeled 4d and 4f should be relabeled 4e

ACTION: We thank the reviewer for noticing all these typos. They have been corrected and as per suggestion, a thorough proof-read was done.

The abstract refers to CRISPR-Cas13 as a “nucleic acid-based” therapeutic agent, while this is partially true due to the gRNA requirement, drawbacks like poor “cellular uptake” and “stability” are not particularly appropriate to the CRISPR-Cas13 platform given the differences in therapeutically relevant delivery used for this technology compared to siRNA and ASOs.

ACTION: We agree with the reviewer that referring to CRISPR-Cas13 as a nucleic acid therapeutic is not appropriate. CRISPR-Cas13 has now been removed from this comparison in the abstract.

oc-2023-000153.R2

Name: Peer Review Information for "Proximity-Induced Nucleic Acid Degrader (PINAD) Approach to Targeted RNA Degradation Using Small Molecules"

Second Round of Reviewer Comments

Reviewer: 1

Comments to the Author

The authors have made all the requested edits and the new data + text have both answered my questions and clarified the data for future readers. I believe the current manuscript better highlights the utility of the PINAD method.

Reviewer: 2

Comments to the Author

Thank you for addressing most of our concerns. Congrats!

Author's Response to Peer Review Comments:

Dear Editor,

We are delighted to read that our work has now been provisionally accepted.

I have uploaded clean files as requested and added the SI paragraph as well as synopsis label. We do not require caption permissions. We only used one graphic for the response letter, but this is not used either in the manuscript or SI.

We wanted to thank you and the reviewers for the excellent comments which helped us providing further support to our claims and improving the manuscript.

With my best wishes, Gonçalo (on behalf of all authors)
